# Systematic Study of Solid-State Fluorescence and Molecular Packing of Methoxy-*trans*-Stilbene Derivatives, Exploration of Weak Intermolecular Interactions Based on Hirshfeld Surface Analysis

**DOI:** 10.3390/ijms24087200

**Published:** 2023-04-13

**Authors:** Natalia Piekuś-Słomka, Magdalena Małecka, Marcin Wierzchowski, Bogumiła Kupcewicz

**Affiliations:** 1 Department of Inorganic and Analytical Chemistry, Nicolaus Copernicus University in Toruń, Ludwik Rydygier Collegium Medicum in Bydgoszcz, Jurasza 2, 85-089 Bydgoszcz, Poland; 2 Department of Physical Chemistry, Faculty of Chemistry, University of Łódź, Pomorska 163/165, 90-236 Łódź, Poland; magdalena.malecka@chemia.uni.lodz.pl; 3 Department of Chemical Technology of Drugs, Poznań University of Medical Sciences, Fredry 10, 61-701 Poznań, Poland; mwierzch@ump.edu.pl

**Keywords:** solid-state fluorescence, *trans*-stilbene, Hirshfeld surface, crystal lattice

## Abstract

In recent years, fluorescent compounds that emit efficiently in the solid state have become particularly interesting, especially those that are easily prepared and inexpensive. Hence, exploring the photophysical properties of stilbene derivatives, supported by a detailed analysis of molecular packing obtained from single-crystal X-ray diffraction data, is a relevant area of research. A complete understanding of the interactions to determine the molecular packing in the crystal lattice and their effect on the material’s physicochemical properties is essential to tune various properties effectively. In the present study, we examined a series of methoxy-*trans*-stilbene analogs with substitution pattern-dependent fluorescence lifetimes between 0.82 and 3.46 ns and a moderate-to-high fluorescence quantum yield of 0.07–0.69. The relationships between the solid-state fluorescence properties and the structure of studied compounds based on X-ray analysis were investigated. As a result, the QSPR model was developed using PLSR (Partial Least Squares Regression). Decomposition of the Hirshfeld surfaces (calculated based on the arrangement of molecules in the crystal lattice) revealed the various types of weak intermolecular interactions that occurred in the crystal lattice. The obtained data, in combination with global reactivity descriptors calculated using HOMO and LUMO energy values, were used as explanatory variables. The developed model was characterized by good validation metrics (RMSE_CAL_ = 0.017, RMSE_CV_ = 0.029, R^2^_CAL_ = 0.989, and R^2^_CV_ = 0.968) and indicated that the solid-state fluorescence quantum yield of methoxy-*trans*-stilbene derivatives was mainly dependent on weak intermolecular C…C contacts corresponding to π-π stacking and C…O/O…C interactions. To a lesser extent and inversely proportional, the fluorescence quantum yield was affected by the interactions of the type O…H/H…O and H…H and the electrophilicity of the molecule.

## 1. Introduction

Stilbene derivatives are considered interesting compounds that transcend various fields due to their biological [1] and photophysical properties. The skeleton of stilbene consists of two phenyl rings bonded by an ethylene bridge. The presence of unsaturated bond results in two diastereoisomeric forms, *E*-1,2-diphenylethylene (*trans*-configuration) and *Z*-1,2-diphenylethylene (*cis*-configuration), but the *trans*-isomer is more common and exhibits a more stable disposition [2]. Additionally, their π-conjugated structure possesses high fluorescence activity; hence they are commonly used as the core structure in synthesizing many fluorescence materials related to biomedicine, fine chemistry, and material science. According to reports, such compounds’ main applications are organic light-emitting diodes, fluorescent probes for medical applications, chemical sensors, and solar cells [3].

Despite many published studies concerning fluorescence in a solid state, a complete and comprehensive understanding still needs to be discovered due to its complexity. Studies conducted in the solid state allow for determining the structural influence of their photophysical properties. Moreover, solvent interactions or concentration effects do not affect the observations. The efficiency of solid-state fluorescence depends on the combination of nonradiative intramolecular deactivation processes and the arrangement of molecules in the crystal lattice, where the net effect causes either quenching or enhancement of the fluorescence quantum yield compared to that of the solution [4,5,6,7]. Among the factors resulting from the molecular arrangement in the crystal lattice, which influences the fluorescent properties, the occurrence of hydrogen bonds and π-π stacking interactions can be detected [8]. However, the literature is uncertain if the presence of these factors has a positive or negative effect on properties such as fluorescence quantum yield or fluorescence lifetime [9,10,11,12,13]. These occurrences are promoted by numerous complex mechanisms, such as aggregation-induced emission (AIE) or aggregation caused-quenching (ACQ), that can co-occur.

Understanding the nature of the interactions that determine the packing of molecules in the crystal lattice and their effect on the physicochemical properties of the materials is essential to achieve the tuning of various properties. No experimental studies have been performed on the solid-state fluorescence of methoxy-*trans*-stilbene derivatives. Therefore, a systematic examination of the effects of molecular orientation on their solid-state photophysical properties must be conducted. Herein, we employed very homogeneous compounds to investigate hindering aspects that could affect the identification and interpretation of the relationships between fluorescent properties and the structure of compounds.

## 2. Results and Discussion

### 2.1. Spectroscopic Properties Measurements and Calculations

Three-dimensional excitation and emission spectra (EEM) were collected for all methoxy-*trans*-stilbene derivatives (exemplary top view of EEM spectra shown in Figure S1, Supplementary file). The wavelengths of the maximum absorption and emission were determined and are summarized together with calculated Stokes shifts in Table 1. The classical two-dimensional emission spectra of all compounds excited by maximum absorption wavelength were also recorded (Figure S2, Supplementary file).

A key parameter for comparison of fluorescent compounds is the photoluminescence (fluorescence) quantum yield (Φ_PL_), a direct measure of the efficiency of converting absorbed light into emitted light. The results of the quantum yield measurements of the tested methoxy-*trans*-stilbene derivatives are presented in Table 2. The lowest Φ_PL_ was determined for compound **MTS-3** (0.07) and the highest for compound **MTS-4** (0.69). The average Φ_PL_ in the tested group of compounds amounted to 0.39. This indicates the great potential of the tested molecules as fluorophores and the vast possibilities of using them in optoelectronics.

The fluorescence lifetime (τ) is the time the fluorophore spends in the excited state before emitting a photon and returning to the ground state. For seven methoxy-*trans*-stilbene derivatives, the analysis was performed based on the one-exponential function matching method. The remaining compounds were analyzed using a two-exponential function. For these molecules, considering the fractional amplitude contribution, the average τ was calculated (Table 2). The compounds with the lowest τ were **MTS-6** and **MTS-9** (0.83 ns and 0.82 ns, respectively). In addition, **MTS-6** showed one of the lowest Φ_PL_ (0.10). On the other hand, **MTS-5** is a compound with the longest τ (3.46 ns) and one of the highest Φ_PL_ (0.64). Radiative (k_r_) and non-radiative (k_nr_) rate constants in Table 2 were calculated based on experimentally measured quantum yields and fluorescence lifetimes using the following equations:

(1) $$k_{r}=\frac{\Phi_{PL}}{\tau }$$

(2) $$k_{nr}=\frac{1-\Phi_{PL}}{\tau }$$

**Figure 1:**
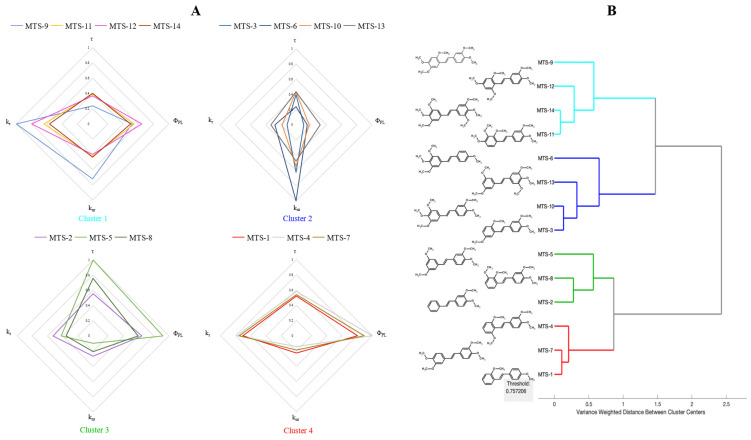
(**A**) Radar charts of fluorescent properties distribution of *trans*-stilbene derivatives (values normalized to 1). (**B**) Cluster analysis result—dendrogram based on solid-state fluorescence properties of studied compounds. Abbreviations: τ, fluorescence lifetime; Φ_PL_, fluorescence quantum yield; k_r_, rate constants of radiative decay; k_nr_, rate constants of non-radiative decay.

In the next step, the studied methoxy-*trans*-stilbene derivatives were quantitatively compared regarding fluorescent properties obtained from measurements and calculations. The results are presented in radar charts (Figure 1A). Due to differences in the analyzed variables’ values, a transformation was performed by dividing each observation by the highest occurring value (after transformation, values of variables ranged from 0 to 1, keeping the relative standard deviations unchanged). Figure 1 shows that although the tested compounds are very similar (the same diphenyl skeleton with an ethene bridge and one type of substituent), they differ significantly in photophysical properties. A cluster analysis was performed to identify compounds with the most similar fluorescence properties. As a clustering method, Ward’s minimum variance algorithm was used. The Euclidean distance was taken as the measure of distance (Figure 1B).

The most homogeneous group (cluster 4) comprises **MTS-1**, **MTS-4**, and **MTS-7**. These compounds present high Φ_PL_, which small values of k_nr_ can explain. Fluorescence lifetimes in this group of compounds took average values. One of the lowest Φ_PL_ values was measured for molecules from cluster 2 (**MTS-3**, **MTS-6**, **MTS-10**, and **MTS-13**). The fluorescence lifetimes of these *trans*-stilbene derivatives were comparable to those from cluster 1, but the k_r_ values were significantly lower. Cluster 1 (**MTS-9**, **MTS-11**, **MTS-12**, and **MTS-14**) consists of molecules with short τ and higher k_nr_, compared to compounds in clusters 3 and 4. The values of k_r_ were varied, while the Φ_PL_ was close to the average values. Cluster 3 is made by **MTS-2**, **MTS-5**, and **MTS-8,** characterized by a lower Φ_PL_ than cluster 4 (except **MTS-5**) but longer τ.

Despite the high structural homogeneity, the tested compounds showed surprisingly different photophysical properties. The analysis indicates that the observed differences in studied properties cannot be easily related to the number of methoxy substituents or their positions in the diphenyl skeleton.

### 2.2. X-ray Structural Studies

Available literature data [13,14,15] indicate a possible relationship between the arrangement of molecules in the crystal lattice and their photophysical properties. X-ray crystallography was performed to understand the effects of the methoxy substitution pattern on the solid-state fluorescence properties. Crystal structures of 8 out of 14 tested *trans*-stilbene derivatives were previously published [16,17,18,19,20] and were taken from the CCDC database. For the remaining six compounds (**MTS-3**, **MTS-8**, **MTS-9**, **MTS-10**, **MTS-13**, and **MTS-14**), attempts were made to obtain high-quality single crystals suitable for X-ray structural studies. Only for **MTS-3**, the conducted measurements did not bring the intended results. The crystal structures of five compounds have been solved and refined. Crystallographic data, and experimental and refinement details are listed in Table 3, while bond lengths, bond angles, and torsion angles are entered into Tables S2–S16 (Supplementary file). Table S17 (Supplementary file) summarizes the characteristics of the crystal lattice of compounds taken from the database and newly researched.

**Figure 2:**
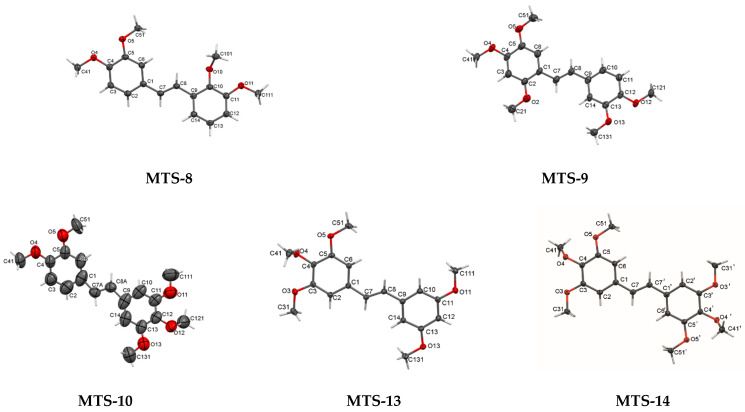
Perspective views of compounds: **MTS-8**, **MTS-9**, **MTS-10**, **MTS-13**, and **MTS-14** with displacement ellipsoids drawn at the 50% probability level.

Perspective views of molecules **MTS-8**, **MTS-9**, **MTS-10**, **MTS-13**, and **MTS-14** are shown in Figure 2. The main molecule consists of two benzene rings (with different located methoxy substituents) connected by a chain of two sp^2^-carbon atoms. All molecular structures are in *trans* arrangement with double C7=C8 central bond (torsion angle C1-C7-C8-C9 nearly half full angle, Tables S4, S7, S10, S13 and S16, Supplementary file). The double bond between C7 and C8 for compound **MTS-10** is disordered over 2 positions with site occupancy ratio of 56%:44% for the A and B parts. In the molecular structure of **MTS-14**, the double bond between C7 and C8 can be indicated as the center of symmetry. The observed lengths of double bonds for **MTS-8**, **MTS-9**, **MTS-13,** and **MTS-14** (ethene bridge, C7-C8 or C7-C7a for **MTS-14**) are longer (1.33 Å–1.34 Å) than the theoretical length (1.32 Å). Moreover, the lengths of bonds between the ethene bridge and benzene rings (C7-C1 and C8-C9) in compounds **MTS-8**, **MTS-9**, **MTS-13,** and **MTS-14** are shorter (1.46–1.48 Å) than the theoretical lengths (1.51Å) indicating the formation of a weak conjugated π-electron system. The presented observations are consistent with those obtained for the other studied *trans*-stilbene derivatives that have been previously published [16,17,18,19,20].

For those 5 molecules, whose crystal structures are described for the first time, 2 benzene rings are in different orientations with a dihedral angle between 2 planes through the rings in the range of 0.00° (**MTS-14**)—24.02° (**MTS-8**) (Table S18, Supplementary file). Exploration the mutual arrangement of the benzene rings in the whole group of the studied *trans*-stilbene derivatives, three types of disposition can be distinguished: co-planar, slightly nonplanar, and nonplanar (twisted). Symmetrical arrangement of substituents relative to the center of the molecule (**MTS-7** and **MTS-14**) determines the parallel arrangement of planes (dihedral angle between two planes 0.00°). In contrast, unsymmetrical molecules with three methoxy groups substituted on adjacent carbons (**MTS-6**, **MTS-10**, **MTS-11**, and **MTS-13**) (especially in the vicinity of the ethene bridge, **MTS-11**) have a nonplanar crystal structure (dihedral angle between two planes: 12.21°, 20.85°, 53.53°, 10.69°, respectively).

The arrangement of the methoxy groups relative to the aromatic rings is also influenced by the presence of substituents at adjacent carbons and the proximity of the ethene bridge (Table S19, Supplementary file). The methoxy groups in *meta* and *para* positions tend to be co-planar with the attached ring. The exception to this is the presence of three methoxy groups at adjacent carbons such as in **MTS-6**, **MTS-10**, **MTS-11**, **MTS-13**, and **MTS-14**. For these compounds, the middle substituent is particularly out-of-plane. In turn, compounds with the methoxy substituent in the *ortho* position (**MTS-1**, **MTS-4**, **MTS-8**, **MTS-9**, and **MTS-12**), have arrangement dependent on the presence of the methoxy group on the adjacent carbon (*meta* position). The absence of such a substituent (**MTS-1**, **MTS-4**, **MTS-9**, **MTS-11**, and **MTS-12**) results in an almost co-planar arrangement. On the other hand, the methoxy groups in *ortho* position in **MTS-8** and **MTS-11** are nearly perpendicular, because of the steric hindrance (substituent in *meta* position).

#### 2.2.1. The Crystal Structure Arrangement

**Figure 3:**
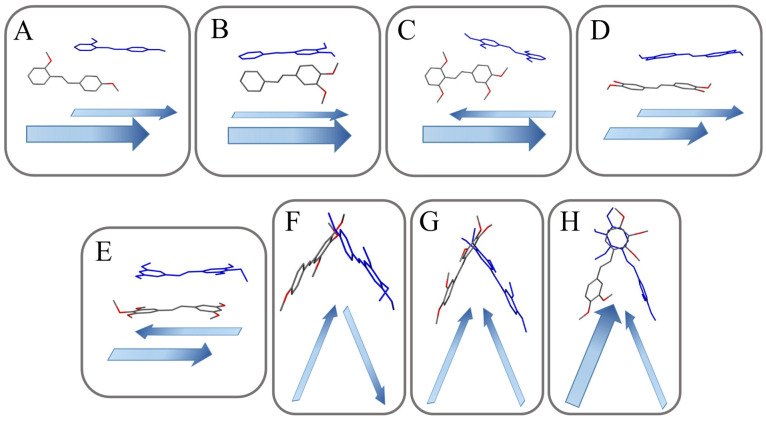
Schematic structural patterns in the investigated stilbene structure. (**A**), slipped perpendicular head to head; (**B**), non-slipped perpendicular head to head; (**C**), slipped perpendicular head to tail; (**D**), slipped parallel head to head; (**E**), slipped parallel head to tail; (**F**), herringbone head to tail; (**G**), herringbone head to head; (**H**), herringbone head to head with inter-twisted.

**Figure 4:**
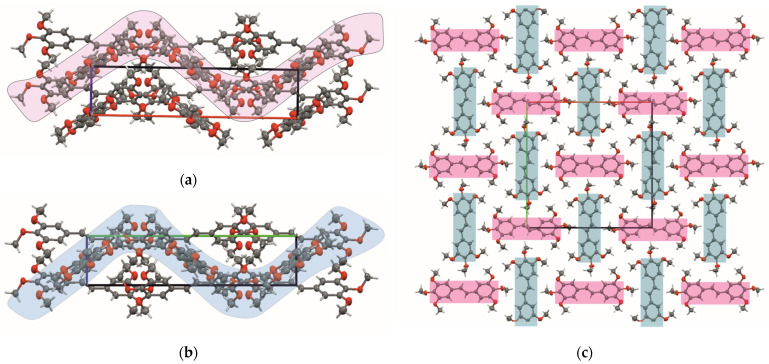
Scheme of molecules arrangement in the crystal structure of **MTS-14:** (**a**) view along *a* axis, (**b**) view along *b* axis, and (**c**) top view of the shits parallel to (001) plane.

Figure 3 and Figure 4 present the types of crystal packing for all 13 compounds.

The arrangement could be categorized as slipped perpendicular head to head (**MTS-1**, Figure 3A), non-slipped perpendicular head to head (**MTS-2** and **MTS-8**, Figure 3B), slipped perpendicular head to tail (**MTS-4** and **MTS-5**, Figure 3C), slipped parallel head to head (**MTS-7**, **MTS-9** and **MTS-12**, Figure 3D), slipped parallel head to tail (**MTS-13**, Figure 3E), herringbone head to tail (**MTS-6**, Figure 3F), herringbone head to head (**MTS-10**, Figure 3G), and herringbone head to head with inter-twisted molecule (**MTS-11**, Figure 3H). In turn, a completely different arrangement of molecules is present for the crystal structure of **MTS-14** (Figure 4), which could be described as thread and warp in the fabric. Herringbone arrangement was observed for compounds with the highest dihedral angle between planes formed by the aromatic rings.

**Figure 5:**
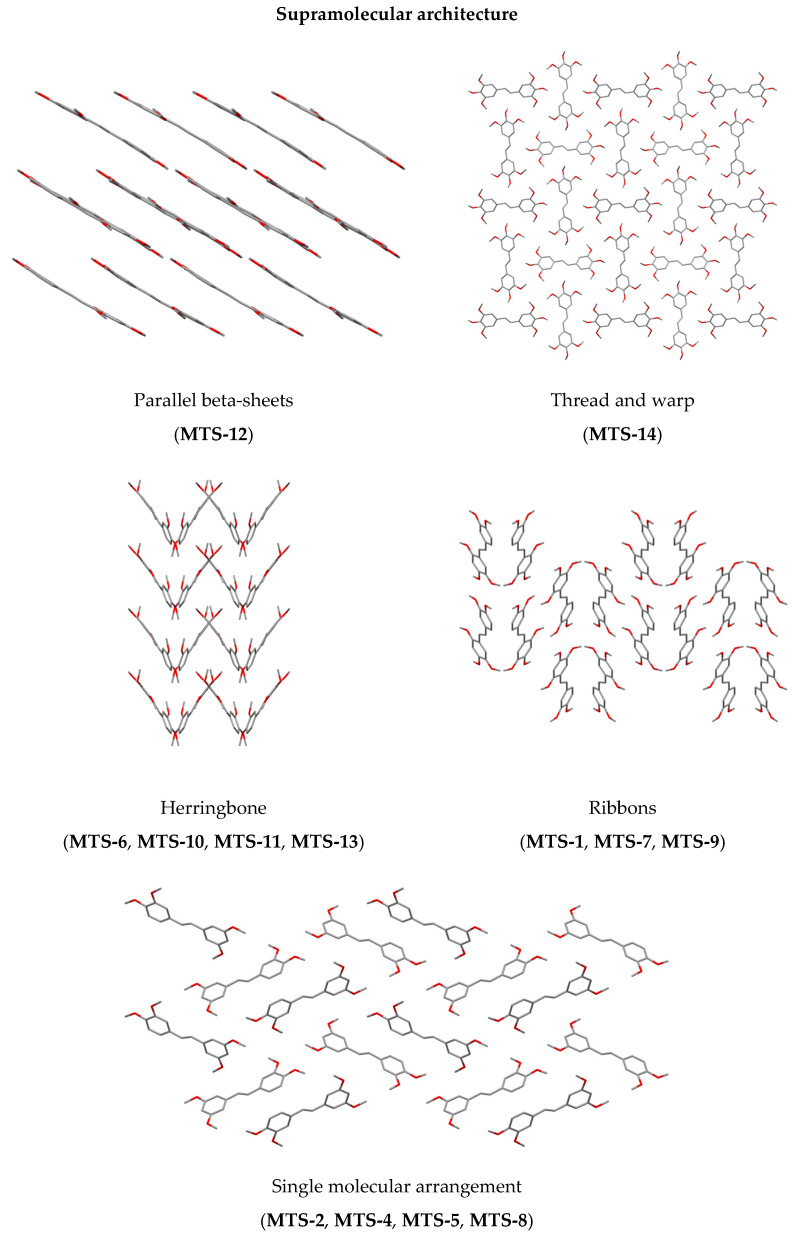
Supramolecular architecture of studied *trans*-stilbene derivatives.

Supramolecular architecture of studied compounds can be categorized into five types (Figure 5). It can be seen that the methoxy substituent in out-of-plane arrangement favors the herringbone system.

Table 4 summarizes intramolecular (Intra) and intermolecular hydrogen bonds occurring in the crystal lattice of stilbene derivatives. Such interactions were not found for compounds **MTS-2**, **MTS-5**, **MTS-6**, and **MTS-13**. Among the tested compounds, the only possible type of hydrogen bonds is C–H···O. The arrangement of -OCH_3_ substituents in adjacent positions or positions 2 or 6 of the phenyl ring (closest to the ethene bridge) favors the formation of intramolecular hydrogen bonds, which occur in as many as 8 out of 13 compounds tested.

**Figure 6:**
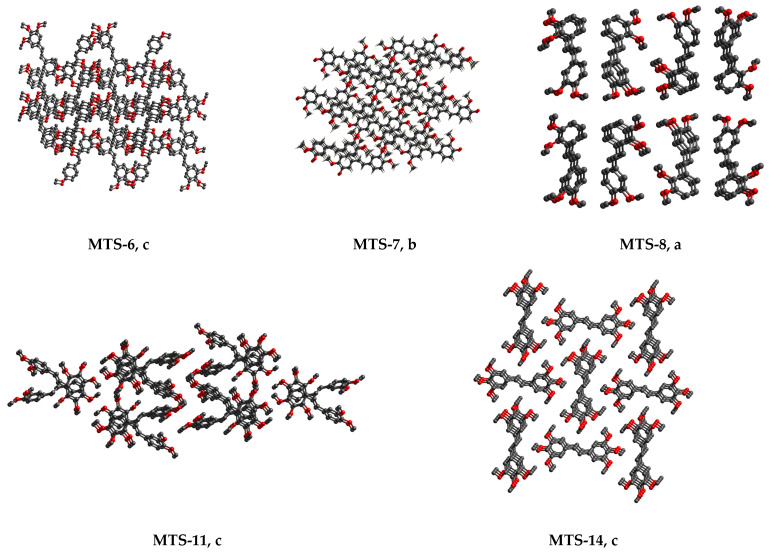
Packing molecules (hydrogen atoms are omitted for clarity) in the crystal lattice of selected *trans*-stilbene derivatives (**MTS-6**, **MTS-7**, **MTS-8**, **MTS-11**, and **MTS-14)**, for which staking interactions (π…π) are observed: a, view along *a* axis; b, view along *b* axis; c, top view of the shifts parallel to 001 plane.

The molecules **MTS-6**, **MTS-7**, **MTS-8**, **MTS-11**, and **MTS-14** in their supramolecular structure are arranged in sheets lying one above the other. Consequently, staking interactions (π…π) occur between molecular layers, which correspond with C…C weak intermolecular interactions in the crystal structure of these compounds (Figure 6) [21]. This will be further discussed in Section 2.2.2. Hirshfeld surface analysis.

#### 2.2.2. Hirshfeld Surface Analysis

Based on the exact spatial structure of molecules, it is possible to identify weak intermolecular interactions. Hirshfeld surface analysis is an effective way to discern intermolecular interactions in the crystal lattice and visualize them. Different colors representing short or long contacts and color intensity indicate the relative strength of the interactions.

**Figure 7:**
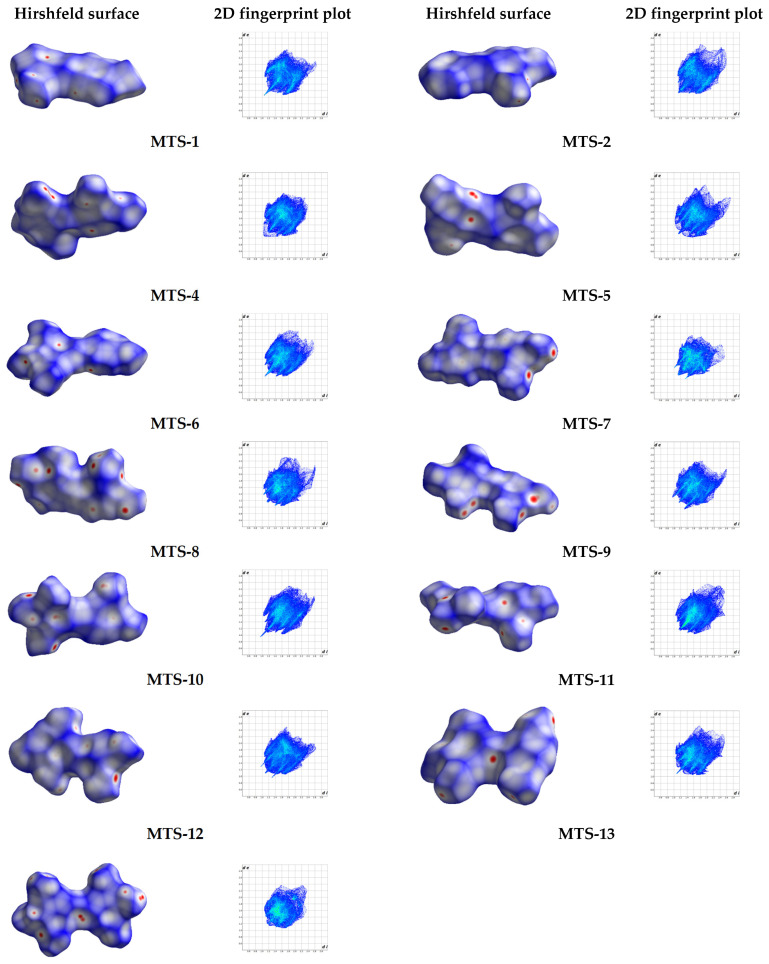
Hirshfeld surfaces mapped with d_norm_ and corresponding 2D fingerprint print of studied compounds.

Figure 7 shows the Hirshfeld surfaces of 13 methoxy-*trans*-stilbene derivatives mapped with d_norm_ (−0.26 to 1.48 Å). Following the calculated Hirshfeld surfaces, 2D fingerprint plots were generated (Figure 7 and Figure S3, Supplementary file). These molecular fingerprints provide qualitative and quantitative information about close intermolecular contacts (Table 5). The most frequent type of contacts in the structure of the tested compounds corresponds to H…H interactions, which account for about 50% of the Hirshfeld surface. C-H…π interactions are visible as characteristic “wings” assigned to the C…H/H…C contacts. In turn, the O…H/H…O interactions form a pair of so-called spikes, the length of which is related (inversely proportional) to the distance of the atoms forming this type of contact. Analysis of the tested compounds’ structural patterns (Figure 3) and the contribution of different intermolecular interactions (Table 5) allows for noticing some relationships. In the crystal lattice of compounds with the arrangement type non-slipped perpendicular head to head (**MTS-2** and **MTS-8**), no contacts for C…O/O…C and O…O were observed. On the other hand, for the head to tail arrangement, both slipped perpendicular (**MTS-4** and **MTS-5**) and slipped parallel (**MTS-13**), favor the occurrence of C…O/O…C interactions. No O…O contacts were identified for *trans*-stilbene derivatives with the herringbone arrangement (**MTS-6**, **MTS-10**, and **MTS-11**). Furthermore, in the crystal lattice of slipped parallel head to head compounds (**MTS-7**, **MTS-9**, and **MTS-12**) the highest contribution of O…O interactions was observed among the tested molecules. Moreover, **MTS-11** with the packing arrangement head to head with inter-twist, and **MTS-14** with packing arrangement thread and warp in the fabric possess the highest contribution of C…C contacts [21].

Comparing the percentage of C…C interactions in the Hirshfeld surface with the motifs of the molecules’ arrangement, it is worth noting that for the compounds **MTS-6**, **MTS-7**, **MTS-8**, **MTS-11**, and **MTS-14,** the percentage of C…C interactions can be distinguished (1.6–2.8%) to be larger than the others. Moreover, exactly these *trans*-stilbene derivatives are arranged in sheets lying one above the other, as shown in Figure 6.

**Figure 8:**
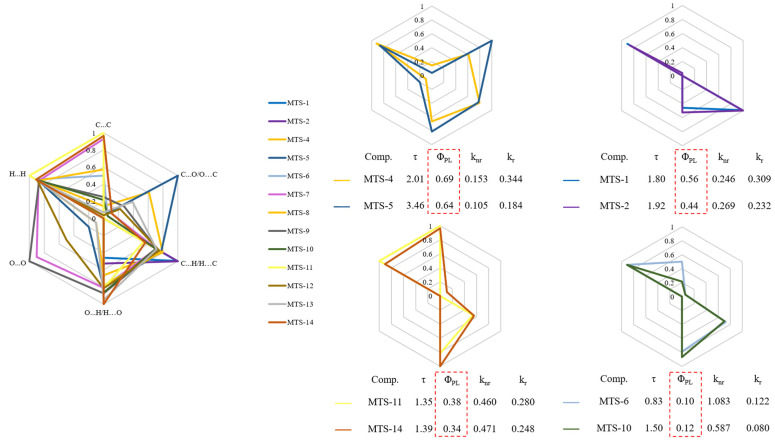
Radar charts plotted based on relative contributions of various intermolecular contacts to the Hirshfeld surface area of studied *trans*-stilbene derivatives (values normalized to 1).

Aiming to visualize the differences between the tested compounds regarding weak intermolecular interactions, the data obtained from the Hirshfeld surface decomposition were plotted on a radar chart (Figure 8). As before, the transformation of the variables to the 0–1 range was performed. In addition, Figure 8 contains fragments of Table 2.

Pairs of compounds with similar contributions to different intermolecular interactions can be identified among the studied molecules. Compounds with a comparable contribution of various types of weak intermolecular contacts were characterized by similar photophysical properties, especially the Φ_PL_. Moreover, for compounds with the highest Φ_PL,_ a much more significant contribution of C…O/O…C type of interactions was observed. On the other hand, the O…O contacts seem to have little influence on the fluorescent properties of the tested *trans*-stilbene derivatives. The analysis demonstrated a relationship between the weak intermolecular interactions occurring in the crystal lattice and the Φ_PL_. However, this relationship was not clear and demanded further investigation.

### 2.3. QSPR Model

Despite many published studies, quantitative understanding or even prediction of fluorescence properties still needs to be improved, mainly due to the mutual interplay between intramolecular and intermolecular factors affecting these properties [8]. For the mentioned reason, attempts have been made to construct the QSPR calibration model (using the PLSR method) based on data from Hirshfeld surface analysis. As additional variables, sums and ratios of various types of weak intermolecular contacts ((O…H/H…O) + (H…H), (C…O/O…C) + (C…C), (H…H) + (C…H/H…C), (C…H/H…C) + (O…H/H…O), (C…H/H…C)/(O…H/H…O), (C…H/H…C)/(H…H), (H…H)/(O…H/H…O)) were used. Moreover, the energy values of the HOMO and LUMO orbitals and the global reactivity descriptors calculated based on them (energy gap, hardness, softness, chemical potential, ionization energy, electronegativity, electron affinity, and electrophilicity) have been added to the set of explanatory variables [22]. The substantial QSPR model, with very good validation metrics, was developed for fluorescence quantum yield. Calibration models for other solid-state fluorescence parameters characterizing methoxy-*trans*-stilbene derivatives (fluorescence lifetime, radiative, and non-radiative rate constants) were also constructed. However, these models had unsatisfactory validation parameters.

**Figure 9:**
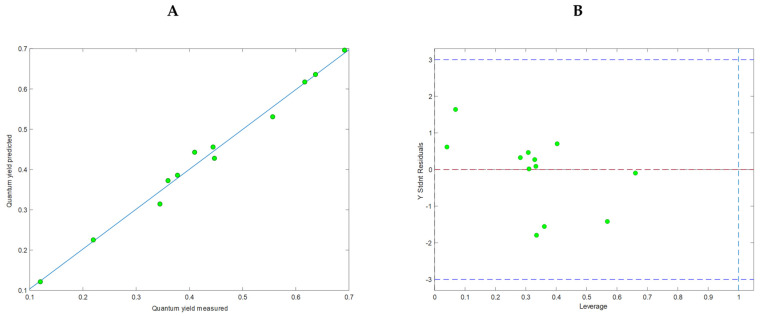
Characteristics of the calculated QSPR model. (**A**) Relationships between measured and calculated values of fluorescence quantum yield using the model. (**B**) Williams plot with a designated applicability domain.

Figure 9A presents the relationship between the measured values of Φ_PL_ and those calculated by the model. The model, constructed with 4 latent variables (LV), had good validation metrics: RMSE_CAL_ = 0.017; RMSE_CV_ = 0.029; R^2^_CAL_ = 0.989, R^2^_CV_ = 0.968 without overfitting and can be described by the following equation:

***ϕ***_***PL***_= 0.979 · **C…C** + 0.859 · **C…O/O…C** − 0.486 · **(O…H/H…O)** + **H…H** − 0.492 · ***ω*** + 0.401 · **(C…H/H…C)/(O…H/H…O)** + 0.030 · **(C…H/H…C)/H…H** (3)

Compound MTS-6 was detected as an outlier and removed from further QSPR analysis.

The permutation test (Y randomization) confirmed the model’s good quality (all three tests: Wilcoxon, Sign Test, and Rand *t*-test, were passed, *p* < 0.05). Moreover, the Williams plot (Figure 9B) pointed out that all molecules were within the applicability domain; thus, the model was statistically acceptable.

**Figure 10:**
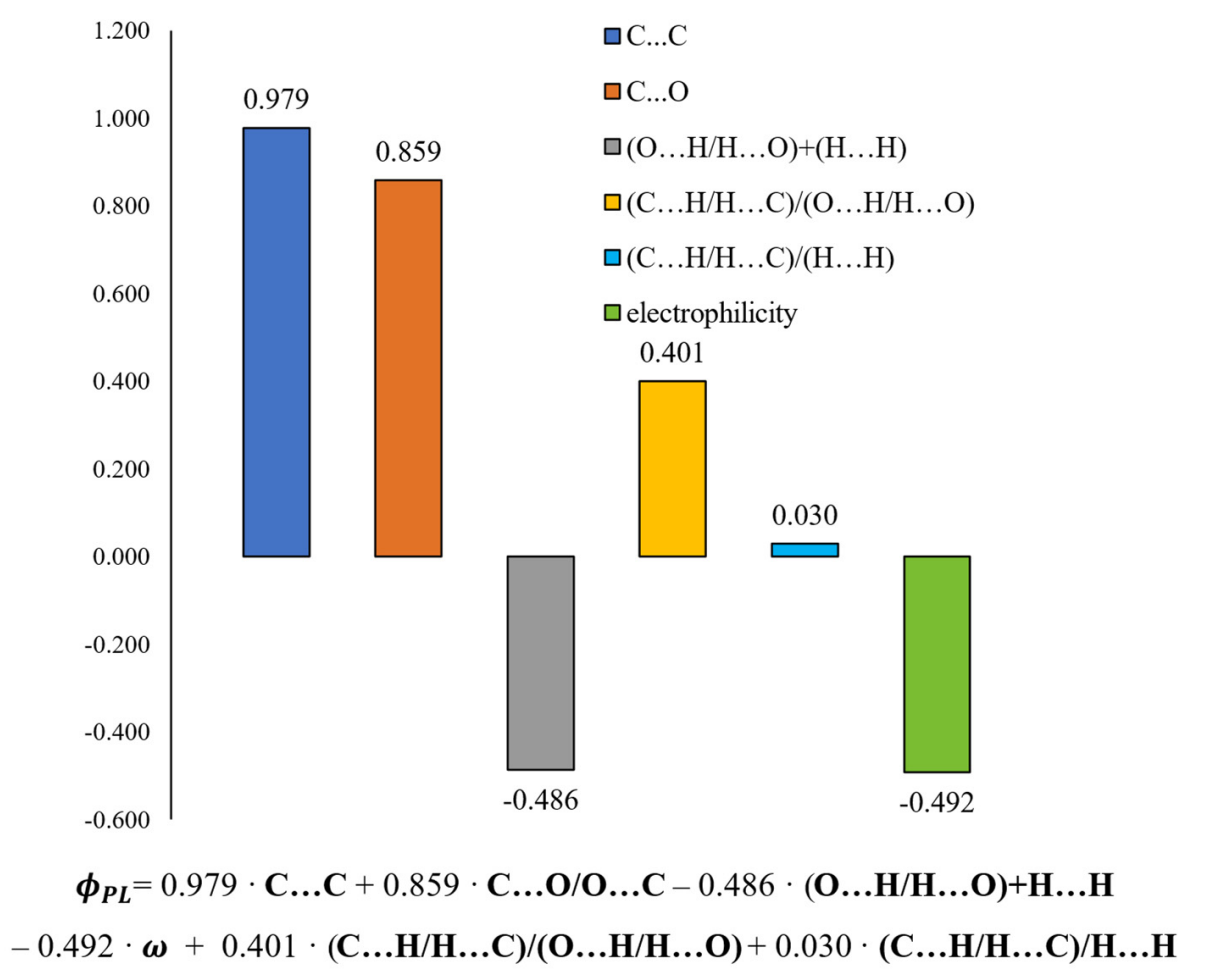
Values of regression coefficients of the calculated QSPR model and its equation.

The calibration model was built based on six variables. The values of the regression coefficients and their positive or negative impact on the predicted variable are presented in Figure 10. A linear equation could describe the relationship between the structure of methoxy-*trans*-stilbene derivatives and their Φ_PL_, also shown in Figure 10.

**Figure 11:**
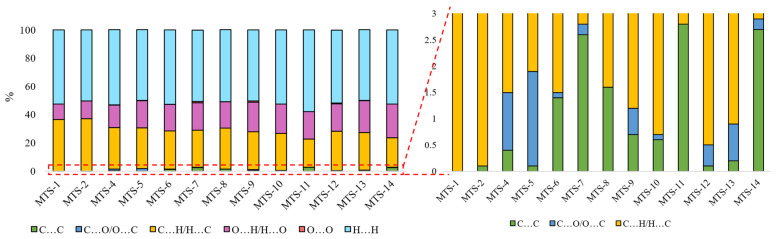
Bar graphs present the percentage share of weak intermolecular interactions occurring in the crystal lattice of *trans*-stilbene derivatives.

**Figure 12:**
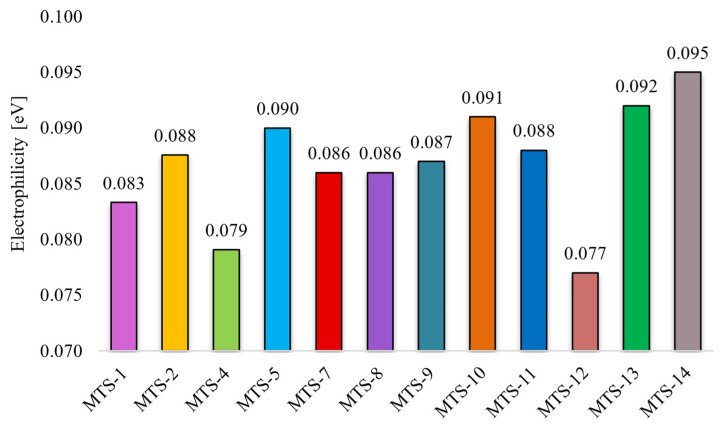
Electrophilicity of selected *trans*-stilbene derivatives.

Intermolecular interactions of the C…C and C…O/O…C types have the greatest and positive impact on the dependent variable (Φ_PL_). Although contacts of this type have a small share in all occurring interactions (ranges 0–1.8% and 0–2.8%, respectively, Figure 11), they are mainly responsible for explaining the variance of the solid-state fluorescence quantum yield of methoxy-*trans*-stilbene derivatives. The compounds with the largest contributions of C…C contacts (green in Figure 11) are **MTS-7**, **MTS-11**, and **MTS-14**. The fluorescence quantum yield of these compounds is, respectively, 0.62, 0.38, and 0.34, i.e., in the case of the last 2, it is below the average Φ_PL_ in the entire group. This was because, in the crystal lattice of these molecules, there was a high level of H…H and O…H/H…O contacts (blue and pink colors in Figure 11), the sum of which is a component of the developed QSPR model with a negative regression coefficient (−0.486 • (O…H/H…O) + (H…H)). Apart from the compound mentioned above (**MTS-7**), the derivatives with the highest Φ_PL_ were **MTS-4** and **MTS-5** (respectively, 0.69 and 0.64). This can be explained by the highest amounts of C…O/O…C interactions in the crystal lattice of these molecules (blue color in Figure 11); the regression coefficient of this variable is 0.859. In addition, the **MTS-4** compound has one of the lowest electrophilicity values. Since the regression coefficient of electrophilicity has a negative sign (−0.492 • ω), the lower ω values give the higher Φ_PL_. Electrophilicity values for compounds analyzed by QSPR are shown in Figure 12.

**Figure 13:**
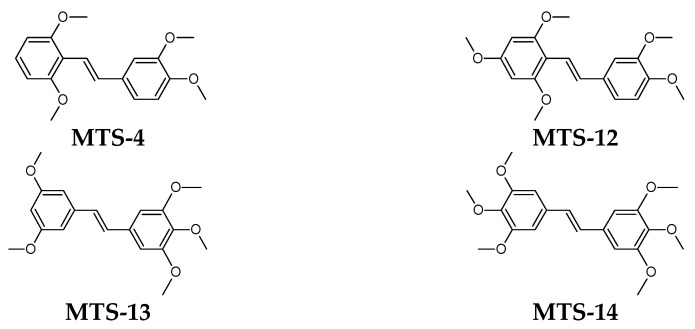
Structural formulas of selected *trans*-stilbene derivatives.

According to [23], a lower electrophilicity value results in an easier transition of the molecule to an excited state. This rule is called the minimum electrophilicity principle (MEP). A molecule with low electrophilicity should have a high value of chemical potential and a high chemical hardness [24]. Facilitating the transition of a molecule to an excited state creates a potentially more significant chance of returning to the ground state by radiative transitions. Therefore, it can be assumed that low electrophilicity may contribute to increased fluorescence quantum yield. This hypothesis is confirmed by the QSPR model obtained in this study, where the regression coefficient for electrophilicity has a negative sign (–0.492 • ω). Compounds with the highest electrophilicity (**MTS-13** and **MTS-14)** are also compounds with one of the lowest Φ_PL_ (0.22 and 0.34). Analyzing the positions of methoxy substituents in the two compounds (**MTS-4** and **MTS-12**) with the lowest and the two (**MTS-13** and **MTS-14**) with the highest electrophilicity, some trends can be noticed. Compounds **MTS-4** and **MTS-12** have substituents in one of the phenyls rings in positions 2 and 6, which is very close to the ethene bridge. This arrangement of -OCH_3_ groups favors the formation of intramolecular hydrogen bonds involving the ethene bridge (see Table 4). In turn, compounds **MTS-13** and **MTS-14** lack methoxy groups in the *ortho* positions and have a symmetrical arrangement of substituents along the stilbene skeleton (Figure 13), which results in high electrophilicity.

Interesting observations were obtained from the analysis of the influence of independent variables on the predicted quantum yield of compound **MTS-1** and, to a lesser extent, **MTS-2**. In the case of both compounds, there are almost no contacts of type C…C and C…O/O…C, which have a positive impact on the quantum yield, while experimentally determined Φ_PL_ values, especially for **MTS-1**, are high—0.56 (for compound **MTS-2** it is 0.44). These derivatives are characterized by extremely high values of the interaction ratios (C…H/H…C)/(O…H/H…O) and (C…H/H…C)/(H…H), which have a positive effect on the Φ_PL_. In addition, the sums of O…H/H…O and H…H interactions are the smallest for these molecules among all compounds, which has a beneficial effect on the Φ_PL_ due to the negative regression coefficient of this component of the equation. A common feature of both compounds, and a potential explanation of the described observation, may be the presence of only two methoxy substituents in these molecules.

**Figure 14:**
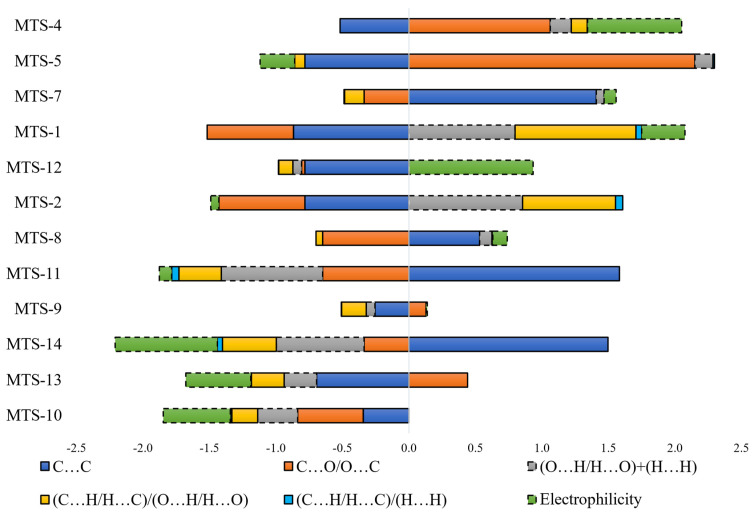
Contribution of individual components of the regression equation on the predicted fluorescence quantum yield (autoscaled values). The hatched bar border indicates variables with a negative regression coefficient.

In quantitative structure–properties analysis, the value of the modeled variable (fluorescence quantum yield) depends on the combination of independent variables included in the model equation and their regression coefficients. Figure 14 presents stacked bars constructed with preprocessed (autoscaled) variables and regression coefficients. The sub-bars give the contribution of individual variables to the value of fluorescence quantum yield and, more precisely, the autoscaled value. The relationship between raw and autoscaled data is presented in Figure S4 (Supplementary file) to decode autoscaled values of fluorescence quantum yield.

The analysis of Figure 14 allows for summarizing the results of the QSPR study. Despite theoretically similar possibilities to the occurrence of C…C intermolecular contacts (number of carbon atoms in the whole group of compounds in the range of 16–20), the value positively influencing the Φ_PL_ (blue bar on the right side of *y*-axis, **MTS-7**, **MTS-8**, **MTS-11**, **MTS-14**) is observed only for compounds which are arranged in sheets lying one above the other (which results in π…π stacking interactions). Such interactions and hydrogen bonds are often indicated as factors that quench the fluorescence quantum yield [10,13,25]. In the group of methoxy-*trans*-stilbene derivatives, the average measured Φ_PL_ of compounds with hydrogen bonds in the crystal lattice is higher (0.44) than the others (0.29). The amount and arrangement of hydrogen bonding affects the rigidity of molecules in the crystal lattice, which can improve the Φ_PL_ by suppressing the vibrational relaxation of excitons [26]. A review of Figure 14 for the effect of C…O/O….C intermolecular interactions indicates that the presence of methoxy substituents on the adjacent carbon atoms is unfavorable for Φ_PL_ (orange bar to the left side of the *y*-axis, **MTS-8**, **MTS-10**, and **MTS-11**). Similar conclusions can be drawn from the study of the component of the equation calculated as the sum of the O…H/H…O and H…H contacts (grey bar on the left side of the *y*-axis). In addition, this variable indicates that a low number of methoxy groups (high grey bar to the right side of the *y*-axis for **MTS-1** and **MTS-2**) is conducive to high Φ_PL_. Exploration of Figure 14 (high green bar on the left side of the *y*-axis, **MTS-14**, and the right side for **MTS-4** and **MTS-12**) together with Figure 12 demonstrates the distal arrangement of the substituents of both phenyl rings in the molecule, resulting in high electrophilicity as being unfavorable for Φ_PL_. Finally, it confirms that methoxy substituents in positions 2 and 6 promote high solid-state quantum yields of *trans*-stilbene derivatives.

## 3. Materials and Methods

**Figure 15:**
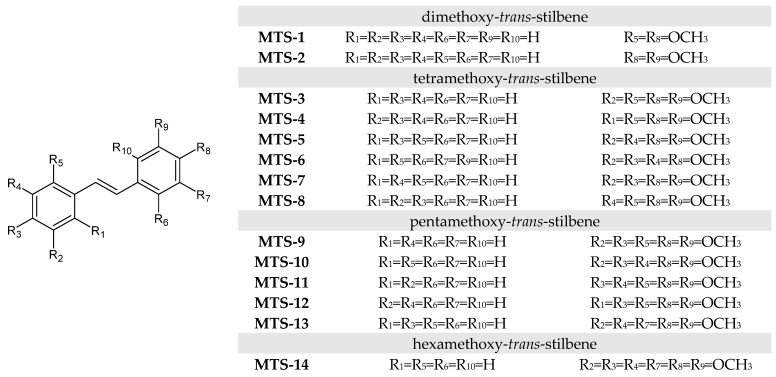
Structure of methoxy-*trans*-stilbenes used in this study.

The subject of the study was 14 methoxy-*trans*-stilbene derivatives **MTS-1–MTS-14** (Figure 15) obtained according to the procedure described in [19,27].

The two-step synthesis started with converting the proper methoxybenzyl chloride to diethyl phosphonates via the Michaelis–Arbuzov reaction (triethyl phosphite, solvent-free, 24 h, 140 °C). In the next step, obtained products were transformed in a Horner–Wadsworth–Emmons reaction with proper aromatic aldehydes (sodium methoxide, dimethylformamide, 24 h, room temperature, next 100 °C for 1.5 h, inert gas—N_2_) into 14 investigated *trans*-polymethoxystilbene derivatives. The Horner–Wadsworth–Emmons reaction is a selective way to possess *trans* isomers. Any traces of *cis* isomers during the realization of the synthetic procedure were not observed. Detailed descriptions of syntheses can be found in the Supplementary file. The structures of synthesized products **MTS-1–MTS-14** were confirmed using NMR and mass spectra.

**Figure 16:**
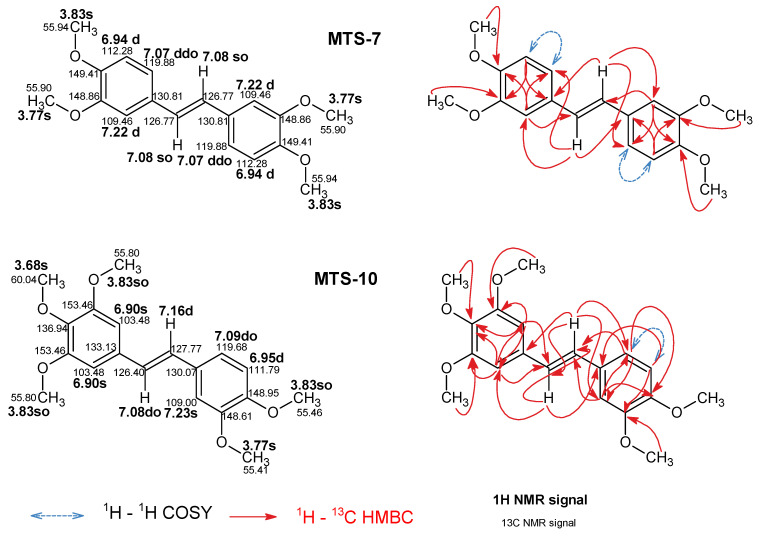
Assigned ^1^H and ^13^C NMR spectra signals according to 2D experiments (^1^H-^1^H COSY, ^1^H-^13^C HSQC, and ^1^H-^13^C HMBC). The abbreviations s, d, dd, and o indicate singlet, doublet, doublet of doublets, and overlaid, respectively. Chemical shifts are expressed as (ppm) in DMSO-*d*_6_.

Complete signal assignments for ^1^H NMR and ^13^C NMR for the studied *trans*-stilbenes, except for **MTS-7** and **MTS-10,** are presented in previous works [19,20]. Figure 16 shows previously unassigned **MTS-7** and **MTS-10** signals of ^1^H NMR and ^13^C NMR spectra recorded in DMSO-*d*_6_. Two-dimensional NMR techniques such as ^1^H-^1^H COSY (Correlation SpectroscopY), ^1^H-^13^C HSQC (Heteronuclear Single Quantum Coherence), and ^1^H-^13^C HMBC (Heteronuclear Multiple-Bond Correlation spectroscopy) were used to assign observed signals to appropriate atoms in the molecules (Supplementary file). The multiplicity of ^1^H NMR signals of vinylene group -HC=CH- strongly depends on the symmetry of the molecule. For example, **MTS-10** signals reveal 2 doublets at 7.16 ppm and 7.08 ppm with, typical for the *trans* vinylene group, the coupling constant ^3^*J*_H-H_ 16.3 Hz. In the case of **MTS-7** vinylene protons in ^1^H, NMR spectra represent a singlet at 7.08 ppm with integration of 2H. ^13^C NMR signals for **MTS-10** are 126.40 ppm and 127.77 ppm. The signal of vinylene carbons for **MTS-7** is observed at 126.77 ppm. Proton signals H2, H5, and H6 of aromatic 3,4-dimethoxybenzene groups in **MTS-7** and **MTS-10** revealed high similarity. For **MTS-7,** the signals mentioned above are observed as a doublet at 7.22 ppm, a doublet at 6.95 ppm, and a doublet of doublets at 7.07 ppm. The analogous signals for **MTS-10** are regarded as a singlet at 7.23 ppm, a doublet at 6.95 ppm, and a doublet at 7.09 ppm. The H2 and H6 protons of a 3,4,5-trimethoxyphenyl fragment of **MTS-10** represent a singlet at 6.90 ppm. The ^1^H NMR methoxy group signals are observed as singlets between 3.68 and 3.83 ppm. The corresponding ^13^C NMR signal is observed in the 55.41 ppm to 60.04 ppm range.

### 3.1. Spectroscopic Measurements

Solid-state fluorescence spectra as excitation–emission matrix (EEM) were recorded on a Shimadzu spectrofluorometer RF-5301 equipped with the solid sample holder. Measurements were conducted at excitation wavelengths 290–390 nm, and emission was recorded at 400–485 nm (the width of both slits was 1.5 nm). Based on the obtained three-dimensional spectra, the wavelengths at which the absorption maximum occurs were determined. This information was used to collect classical, two-dimensional emission spectra (excitation was carried out using the wavelength at which the absorption maximum occurs). The Panorama Pro software [28] was used to measure and analyze the data.

Photoluminescence quantum yield was measured using an FLS980 spectrofluorometer (Edinburgh Instruments) equipped with an integrating sphere. The Spectralon (Labsphere, Inc., North Sutton, NH, USA) light scattering standard was used as a measurement reference. Depending on the properties of the compound, different analysis parameters were used. The wavelength of the excitation ranged from 330 to 390 nm. The beginning of the emission measurement range was between 320 and 370 nm, while its end for all compounds was 700 nm. The width of the excitation gap was 8.0 nm (except for compound **MTS-7** for which it was 7.0 nm), and the emission gap was 0.1 nm (except for compounds **MTS-2** and **MTS-8**, for which it was 0.2 nm). The step was 0.2 nm for all analyses, and the counting time was 0.3 s. Quantum yield calculations were performed using software (F980) provided by Edinburgh Instruments.

The fluorescence lifetime measurements were carried out using an FLS980 spectrofluorometer (Edinburgh Instruments) with a pulsed diode laser. A 360 nm diode laser was used as an excitation source. Depending on the sample, the emission wavelength was in the range of 395–445 nm. The excitation slit was 0.03 nm wide, while the emission slit was 0.3–2.0 nm. Fluorescence lifetime measurements were analyzed using Edinburgh Instruments software based on a one- or two-exponential fitting method without deconvolution.

### 3.2. Computational Methods

Semi-empirical method AM1 in a vacuum was employed to obtain the optimized geometries for studied compounds. The theoretical calculations of the molecular orbitals, HOMO and LUMO energies and, based on them, global reactivity descriptors (energy gap, ionization energy, electron affinity, hardness, softness, electronegativity, chemical potential, electrophilicity [22]) were performed in the Gaussian09 package [29] in PL-Grid infrastructure [30]. The quantitative analysis of the relationship between the crystal structure of compounds and their fluorescence properties was performed with the use of Partial Least Squares Regression (PLSR), using the PLS-Toolbox 7.5 [31] in Matlab software version R2020b [32]. Before performing the calculations, autoscaling of variables (independent and dependent variable) was performed, while their selection was based on the analysis of the VIP (variable importance in projection plot). Internal validation was performed using the “leave one out” method. Hierarchical cluster analysis was performed with STATISTICA v.13.3 software [33].

### 3.3. Crystallographic Data

The crystal structures of compounds **MTS-1**, **MTS-2**, **MTS-4**, **MTS-5**, **MTS-6**, **MTS-7**, **MTS-11**, and **MTS-12** were obtained from the Cambridge Crystallographic Data Centre (CCDC) and were published in [16,17,18,19,20]. Slow evaporation (SV) or vapor diffusion (VD) methods were used for creating single crystals of the other *trans*-stilbene derivatives (**MTS-3**, **MTS-8**, **MTS-9**, **MTS-10**, **MTS-13**, and **MTS-14**), using the solvents methanol (**MTS-8**, SV), a mixture of ethanol and methanol (**MTS-9**, **MTS-10**, SV), isopropanol (**MTS-14**, SV), and the mixture of toluene and ethanol (**MTS-13**, VP) (Sigma-Aldrich). For the **MTS-3**, obtaining a single crystal of sufficient quality for X-ray structural studies was impossible.

X-ray data for compounds **MTS-8**, **MTS-9**, **MTS-10**, **MTS-13**, and **MTS-14** were measured from single crystal using an XtaLAB Synergy, Dualflex diffractometer with an Hypix detector. The temperature of measurements was 100K for **MTS-8** and **MTS-14**, 150K for **MTS-9** and **MTS-13,** and 293K for **MTS-10**. In all cases, the multiscan absorption correction was performed (CrysAlisPro 1.171.41.119a (Rigaku Oxford Diffraction, 2021). All structures were solved by direct methods using SHELXT [34] and further refined on *F*^2^ using SHELXL-2014/7 [35]. All non-hydrogen atoms were refined anisotropically. The atoms C7 and C8 for compound **MTS-10** are disordered and refined with an occupancy ratio of 56%:44% for the A and B parts. The positions of hydrogen atoms were calculated from known geometry (C-H bond lengths at 0.95 and 0.98 Å for aromatic CH and methyl CH_3_ atoms, respectively) and treated as riding where the isotropic thermal parameters of these hydrogen atoms were fixed as *U*_iso_(H) = 1.5*U*_eq_(C) for H atoms from a methyl group and *U*_iso_(H) = 1.2*U*_eq_(C) for all remaining atoms. PLATON [36] and MERCURY [37] were used to identify molecular geometries and hydrogen-bond patterns. Basic experimental details and crystallographic data are presented in Table 3. Further crystallographic information for the structures reported in this paper may be obtained free of charge on application to CCDC, 12 Union Road, Cambridge CG21, EZ, UK [fax: (44) 1223-336-033; e-mail: deposit@ccdc.cam.ac.uk] on quoting the depository numbers: CCDC 2179049 (**MTS-8**), 2179050 (**MTS-9**), 2179054 (**MTS-10**), 2179056 (**MTS-13**), and 2179055 (**MTS-14**).

### 3.4. Hirshfeld Surface Analysis

Crystal Explorer v. 21.3 [38] software was used to generate the Hirshfeld surfaces [39] based on the data from X-ray studies. Hydrogen atom bond lengths were normalized to standard neutron values (C−H = 1.083 Å, O−H = 0.983 Å) to ensure internal consistency, which is essential in structures’ comparisons [40]. The normalized contact distance (d_norm_) is based on both d_e_ (the distance from a point on the surface to the nearest atom outside the surface) and d_i_ (the distance from a point on the surface to the nearest atom inside the surface) and van der Waals radii of the atom, given by Equation 4, enables the identification of the regions of particular importance to intermolecular interactions [41]. The negative value of d_norm_ (red color) or positive value of d_norm_ (blue color) occurs when intermolecular contacts are shorter or longer than van der Waals separations.

(4) $$d_{norm}=\frac{d_{i}-r_{i}^{vdW}}{r_{i}^{vdW}}+\frac{d_{e}-r_{e}^{vdW}}{r_{e}^{vdW}}$$

## 4. Conclusions

In summary, the group of 14 methoxy-*trans*-stilbene derivatives was studied to gain insight into the quantitative structure–solid-state fluorescence properties relationship. Five molecules have been characterized by X-ray crystallography. The crystallographic data for other compounds were from CCDC. Fluorescent properties in the solid state were reported for all tested compounds. Interestingly, a slight change in the substituents pattern of compounds significantly affects their molecular packing and photophysical behavior. To determine the quantitative relationships between solid-state fluorescence quantum yield (Φ_PL_) and molecular structure, a calibration model was built using the PLSR algorithm. The model’s acceptable validation parameters indicate the successful application of weak intermolecular interactions, quantified using Hirshfeld surface analysis, to modeling the Φ_PL_ suggests their predictive potential. The interpretation of the model-building variables (different types of weak contacts and electrophilicity) demonstrates a beneficial effect on the observed Φ_PL_ of π…π stacking interactions, the occurrence of hydrogen bonds, and the *ortho* position of methoxy substituents.

## Figures and Tables

**Table 1:** Characteristics of three-dimensional excitation and emission spectra of studied compounds.

Compound	Maximum Absorption Wavelength $\lambda_{max,exc}\;[\mathrm{nm}]$	Maximum Emission Wavelength, $\lambda_{max,em}\;[\mathrm{nm}]$	Stokes Shift [cm^−1^]
**MTS-1**	375	450	4444
**MTS-2**	367	409	2798
**MTS-3**	389	445	3235
**MTS-4**	375	422	2970
**MTS-5**	376	442	3971
**MTS-6**	367	394, 412	1867, 2976
**MTS-7**	384	435, 455	3053, 4064
**MTS-8**	374	414	2583
**MTS-9**	419	455	1888
**MTS-10**	373	419	2943
**MTS-11**	358	387, 420	2093, 4123
**MTS-12**	383	432, 458	2961, 4276
**MTS-13**	367	395	1931
**MTS-14**	367	392, 413	1738, 3035

**Table 2:** Fluorescence lifetime (τ), quantum yield (Φ_PL_), and rate constants of radiative (k_r_) and non-radiative decay (k_nr_) of compounds **MTS-1–MTS-14** in the solid-state.

Compound	Fluorescence Lifetime ^1^ [ns] (Fractional Amplitudes [%])		Average Fluorescence Lifetime (τ) [ns]	Fluorescence Quantum Yield ^2^ (Φ_PL_)	k_nr_·10^8^ [s^−1^]	k_r_·10^8^ [s^−1^]
	τ_1_	τ_2_				
**MTS-1**	1.8 (100%)	–	1.80	0.56	2.46	3.09
**MTS-2**	1.92 (100%)	–	1.92	0.44	2.89	2.32
**MTS-3**	1.37 (100%)	–	1.37	0.07	6.78	0.52
**MTS-4**	2.01 (100%)	–	2.01	0.69	1.53	3.44
**MTS-5**	2.68 (49%)	4.20 (51%)	3.46	0.64	1.05	1.84
**MTS-6**	0.83 (100%)	–	0.83	0.10	10.83	1.22
**MTS-7**	1.06 (37%)	2.34 (63%)	1.87	0.62	2.05	3.31
**MTS-8**	2.62 (100%)	–	2.62	0.41	2.24	1.56
**MTS-9**	0.34 (63%)	1.63 (37%)	0.82	0.36	7.80	4.39
**MTS-10**	0.88 (65%)	2.64 (35%)	1.50	0.12	5.87	0.80
**MTS-11**	1.12 (74%)	2.01 (26%)	1.35	0.38	4.60	2.80
**MTS-12**	1.04 (70%)	1.82 (30%)	1.27	0.45	4.34	3.51
**MTS-13**	1.51 (100%)	–	1.51	0.22	5.17	1.45
**MTS-14**	1.39 (100%)	–	1.39	0.34	4.71	2.48

^1^ Details on the measurement precision are in the Supplementary file Table S1. ^2^ The error of the quantum yield determination method was 5%.

**Table 3:** Crystallographic data, and experimental and refinement details.

Compound	MTS-8	MTS-9	MTS-10	MTS-13	MTS-14
ID CCDC	2179049	2179050	2179054	2179056	2179055
Chemical formula	C_18_H_20_O_4_	C_19_H_22_O_5_	C_19_H_22_O_5_	C_19_H_22_O_5_	C_20_H_24_O_6_
Formula weight [g/mol]	300.349	330.375	330.375	330.375	360.401
Crystal system	orthorhombic	monoclinic	orthorhombic	monoclinic	tetragonal
Space group	P bca	P 2_1_/c	P ca2_1_	P 2_1_/c	P 4_2_/n
*a*, *b*, *c* [Å]	5.2197(2)	10.5321(6)	14.3780(5)	11.2909(4)	19.7692(5)
	23.4997(10)	19.5400(13)	8.0758(4)	10.8019(3)	19.7692(5)
	25.0186(13)	8.3901(6)	15.0617(7)	14.4398(4)	4.5558(2)
*α*, *β*, *γ* [°]	90.00	90.00	90.00	90.00	90.00
	90.0	103.754(7)	90.00	108.017(3)	90.00
	90.00	90.00	90.00	90.00	90.00
V [Å^3^]	3068.82	1677.15	1748.87	1674.77	1780.5
Z	8	4	4	4	4
R_1_	4.30	8.01	5.41	3.93	3.41

**Table 4:** Hydrogen bonds occurring in the crystal lattice of studied *trans*-stilbene derivatives.

Compound	Type	Donor–H···Acceptor	d(D–H)	d(H···A)	d(D···A)	<D–H···A
**MTS-1**	Intra	C7–H7···O17	0.94(2)	2.32(2)	2.725(2)	105(2)
**MTS-4**	Intra	C7–H3···O2	0.96(2)	2.29(2)	2.711(2)	106(2)
	Intra	C8–H4···O1	0.97(2)	2.22(2)	2.808(2)	119(2)
**MTS-7**		C8–H4···O1 ^i^	0.97(2)	2.56(2)	3.319(2)	136(2)
		C8–H4···O2 ^i^	0.97(2)	2.50(2)	3.427(2)	160(2)
**MTS-8**		C51–H5A···O10 ^ii^	0.98	2.54	3.484(2)	162
	Intra	C101–H10C···O11	0.98	2.34	2.946(2)	119
		C111–H11C···O5 ^iii^	0.98	2.54	3.509(2)	168
		C12–H12···O4 ^iii^	0.95	2.60	3.526(2)	166
**MTS-9**		C21–H2C···O2 ^iv^	0.96	2.49	3.369(4)	153
		C51–H5A···O12 ^v^	0.96	2.41	3.312(6)	157
	Intra	C7–H7···O2	0.93	2.37	2.723(4)	102
		C41–H41B···O13 ^vi^	0.96	2.48	3.436(4)	172
**MTS-10**		C41–H4A···O12 ^vii^	0.96	2.58	3.320(11)	135
	Intra	C121–H12C···O11	0.96	2.35	2.930(10)	119
**MTS-11**	Intra	C8–H7···O3	0.96	2.51	3.090(3)	119
	Intra	C9–H10···O2	0.96	2.55	3.117(3)	118
**MTS-12**	Intra	C3–H1···O4	0.93	2.27	2.692(3)	107
	Intra	C9–H3···O1	0.93	2.23	2.857(3)	124
		C18–H17···O3 ^viii^	0.96	2.40	3.344(3)	169
**MTS-14**	Intra	C41–H4C···O5	0.98	2.56	3.087(2)	114

Symmetry codes: (i): −x, −y, −z; (ii): 1/2 + x, 3/2 − y, 1 − z, (iii): 1/2 − x, 1/2 + y, z; (iv): 1 − x, 1 − y, 2 − z; (v): −1+x, 3/2 − y, 1/2 + z; (vi): −1 + x,y,1 + z; (vii): 1/2 − x,−1 + y,1/2+z; (viii): −1 + x,y,−1 + z.

**Table 5:** Relative contributions in the percentage of various intermolecular contacts to the Hirshfeld surface area of studied *trans*-stilbene derivatives.

Compound	C…C	C…O/O…C	C…H/H…C	O…H/H…O	O…O	H…H
**MTS-1**	0	0	36.7	10.9	0	52.4
**MTS-2**	0.1	0	37.1	12.5	0	50.3
**MTS-4**	0.4	1.1	29.5	15.8	0.1	53.2
**MTS-5**	0.1	1.8	28.8	19.3	0.2	49.9
**MTS-6**	1.4	0.1	27.0	18.8	0	52.7
**MTS-7**	2.6	0.2	26.3	19.3	0.9	50.6
**MTS-8**	1.6	0	28.9	18.7	0	50.9
**MTS-9**	0.7	0.5	26.8	20.8	1.0	50.2
**MTS-10**	0.6	0.1	26.2	20.6	0	52.5
**MTS-11**	2.8	0	20.0	19.4	0	57.8
**MTS-12**	0.1	0.4	27.9	19.4	0.5	51.6
**MTS-13**	0.2	0.7	26.5	22.6	0.1	50.0
**MTS-14**	2.7	0.2	20.8	23.8	0	52.5

## Data Availability

Not applicable.

## Supplementary Materials

The following supporting information can be downloaded at https://www.mdpi.com/article/10.3390/ijms24087200/s1.
