# Supplementary material for: Systematic Study of Solid-State Fluorescence and Molecular Packing of Methoxy-trans-Stilbene Derivatives, Exploration of Weak Intermolecular Interactions Based on Hirshfeld Surface Analysis
Source: Int J Mol Sci. 2023 Apr 13;24(8):7200. doi: 10.3390/ijms24087200 (PMC10139126; doi:10.3390/ijms24087200)

checkCIF/PLATON report

Structure factors have been supplied for datablock(s) S12_deposit_new

THIS REPORT IS FOR GUIDANCE ONLY. IF USED AS PART OF A REVIEW PROCEDURE FOR PUBLICATION, IT SHOULD NOT REPLACE THE EXPERTISE OF AN EXPERIENCED CRYSTALLOGRAPHIC REFEREE.

No syntax errors found. CIF dictionary Interpreting this report

Datablock: S12_deposit_new

```
Bond precision:   C-C = 0.0048 A          Wavelength=1.54184

Cell:               a=14.3780(5)      b=8.0758(4)      c=15.0617(7)
                    alpha=90          beta=90          gamma=90
Temperature:        293 K

                 Calculated                    Reported
Volume           1748.87(13)                   1748.87(13)
Space group      P c a 21                      P c a 21
Hall group       P 2c -2ac                     P 2c -2ac
Moiety formula   C19 H22 O5                    ?
Sum formula      C19 H22 O5                    C19 H22 O5
Mr               330.37                        330.36
Dx,g cm-3        1.255                         1.255
Z                4                             4
Mu (mm-1)        0.742                         0.742
F000             704.0                         704.0
F000'            706.28
h,k,lmax         17,9,18                       17,9,17
Nref             3148[ 1641]                   2629
Tmin,Tmax        0.930,0.954
Tmin'            0.930

Correction method= Not given

Data completeness= 1.60/0.84       Theta(max)= 67.458

                                                            wR2(reflections)=
R(reflections)= 0.0543( 1979)                               0.1565( 2629)

S = 0.866                 Npar= 225
```

The following ALERTS were generated. Each ALERT has the format
test-name_ALERT_alert-type_alert-level.
Click on the hyperlinks for more details of the test.

Alert level C

```
STRVA01_ALERT_4_C           Flack parameter is too small
           From the CIF: _refine_ls_abs_structure_Flack   -0.250
           From the CIF: _refine_ls_abs_structure_Flack_su    0.150
PLAT052_ALERT_1_C Info on Absorption Correction Method   Not Given     Please Do !
PLAT089_ALERT_3_C Poor Data / Parameter Ratio (Zmax < 18) ........       7.29 Note
PLAT241_ALERT_2_C High   'MainMol' Ueq as Compared to Neighbors of         C1 Check
PLAT241_ALERT_2_C High   'MainMol' Ueq as Compared to Neighbors of         C9 Check
PLAT260_ALERT_2_C Large Average Ueq of Residue Including        O4      0.108 Check
PLAT340_ALERT_3_C Low Bond Precision on  C-C Bonds ..............    0.00483 Ang.
PLAT906_ALERT_3_C Large K Value in the Analysis of Variance ......      3.589 Check
```

Alert level G

```
PLAT003_ALERT_2_G Number of Uiso or Uij Restrained non-H Atoms ...          16 Report
PLAT178_ALERT_4_G The CIF-Embedded .res File Contains SIMU Records           4 Report
PLAT186_ALERT_4_G The CIF-Embedded .res File Contains ISOR Records           4 Report
PLAT188_ALERT_3_G A Non-default SIMU Restraint Value has been used      0.0050 Report
PLAT188_ALERT_3_G A Non-default SIMU Restraint Value has been used      0.0050 Report
PLAT188_ALERT_3_G A Non-default SIMU Restraint Value has been used      0.0050 Report
PLAT188_ALERT_3_G A Non-default SIMU Restraint Value has been used      0.0050 Report
PLAT199_ALERT_1_G Reported _cell_measurement_temperature ..... (K)         293 Check
PLAT200_ALERT_1_G Reported   _diffrn_ambient_temperature ..... (K)         293 Check
PLAT301_ALERT_3_G Main Residue  Disorder .............(Resd  1  )           8% Note
PLAT860_ALERT_3_G Number of Least-Squares Restraints ............          181 Note
PLAT883_ALERT_1_G No Info/Value for _atom_sites_solution_primary .     Please Do !
PLAT915_ALERT_3_G No Flack x Check Done: Low Friedel Pair Coverage          66 %
PLAT941_ALERT_3_G Average HKL Measurement Multiplicity ..........          3.9 Low
PLAT961_ALERT_5_G Dataset Contains no Negative Intensities .......     Please Check
PLAT965_ALERT_2_G The SHELXL WEIGHT Optimisation has not Converged     Please Check
PLAT967_ALERT_5_G Note: Two-Theta Cutoff Value in Embedded .res ..       135.0 Degree
PLAT978_ALERT_2_G Number C-C Bonds with Positive Residual Density.           4 Info
```

0 **ALERT level A** = Most likely a serious problem - resolve or explain
0 **ALERT level B** = A potentially serious problem, consider carefully
8 **ALERT level C** = Check. Ensure it is not caused by an omission or oversight
18 **ALERT level G** = General information/check it is not something unexpected

4 ALERT type 1 CIF construction/syntax error, inconsistent or missing data
6 ALERT type 2 Indicator that the structure model may be wrong or deficient
11 ALERT type 3 Indicator that the structure quality may be low
3 ALERT type 4 Improvement, methodology, query or suggestion
2 ALERT type 5 Informative message, check

It is advisable to attempt to resolve as many as possible of the alerts in all categories. Often the minor alerts point to easily fixed oversights, errors and omissions in your CIF or refinement strategy, so attention to these fine details can be worthwhile. In order to resolve some of the more serious problems it may be necessary to carry out additional measurements or structure refinements. However, the purpose of your study may justify the reported deviations and the more serious of these should normally be commented upon in the discussion or experimental section of a paper or in the "special_details" fields of the CIF. checkCIF was carefully designed to identify outliers and unusual parameters, but every test has its limitations and alerts that are not important in a particular case may appear. Conversely, the absence of alerts does not guarantee there are no aspects of the results needing attention. It is up to the individual to critically assess their own results and, if necessary, seek expert advice.

Publication of your CIF in IUCr journals

A basic structural check has been run on your CIF. These basic checks will be run on all CIFs submitted for publication in IUCr journals (*Acta Crystallographica*, *Journal of Applied Crystallography*, *Journal of Synchrotron Radiation*); however, if you intend to submit to *Acta Crystallographica Section C* or *E* or *IUCrData*, you should make sure that full publication checks are run on the final version of your CIF prior to submission.

Publication of your CIF in other journals

Please refer to the *Notes for Authors* of the relevant journal for any special instructions relating to CIF submission.

PLATON version of 28/11/2022; check.def file version of 28/11/2022

Datablock S12_deposit_new - ellipsoid plot

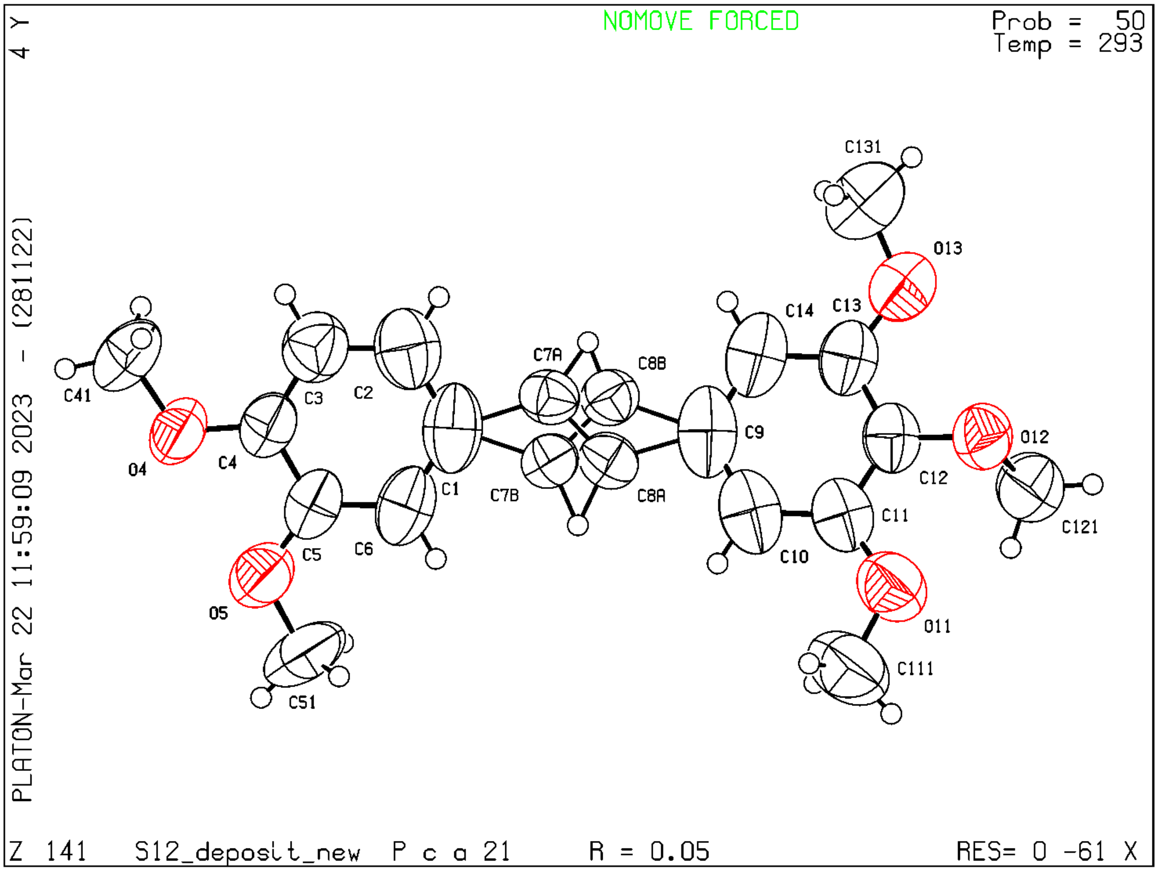

Supplement: Supplementary file 1 [file ijms-24-07200-s001.zip › checkcif_MTS10.pdf]
