# Supplementary material for: Systematic Study of Solid-State Fluorescence and Molecular Packing of Methoxy-trans-Stilbene Derivatives, Exploration of Weak Intermolecular Interactions Based on Hirshfeld Surface Analysis
Source: Int J Mol Sci. 2023 Apr 13;24(8):7200. doi: 10.3390/ijms24087200 (PMC10139126; doi:10.3390/ijms24087200)

checkCIF/PLATON report

Structure factors have been supplied for datablock(s) B9_S9_exp63_deposit_new

THIS REPORT IS FOR GUIDANCE ONLY. IF USED AS PART OF A REVIEW PROCEDURE FOR PUBLICATION, IT SHOULD NOT REPLACE THE EXPERTISE OF AN EXPERIENCED CRYSTALLOGRAPHIC REFEREE.

No syntax errors found. CIF dictionary Interpreting this report

Datablock: B9_S9_exp63_deposit_new

Bond precision:	C-C = 0.0044 A		Wavelength=0.71073
Cell:	a=10.5321(6)	b=19.5400(13)	c=8.3901(6)
	alpha=90	beta=103.754(7)	gamma=90
Temperature:	150 K		

	Calculated	Reported
Volume	1677.2(2)	1677.1(2)
Space group	P 21/c	P 21/c
Hall group	-P 2ybc	-P 2ybc
Moiety formula	C19 H22 O5	?
Sum formula	C19 H22 O5	C19 H22 O5
Mr	330.37	330.36
Dx,g cm-3	1.308	1.308
Z	4	4
Mu (mm-1)	0.094	0.094
F000	704.0	704.0
F000'	704.39	
h,k,lmax	13,24,10	13,24,10
Nref	3477	3476
Tmin,Tmax	0.997,0.998	0.722,1.000
Tmin'	0.997	

Correction method= # Reported T Limits: Tmin=0.722 Tmax=1.000
AbsCorr = MULTI-SCAN

Data completeness= 1.000 Theta(max)= 26.497

R(reflections)= 0.0808(2442) wR2(reflections)= 0.2061(3476)

S = 1.115 Npar= 222

The following ALERTS were generated. Each ALERT has the format

test-name_ALERT_alert-type_alert-level.

Click on the hyperlinks for more details of the test.

Alert level C

```
PLAT340_ALERT_3_C Low Bond Precision on  C-C Bonds ..............     0.0044 Ang.
PLAT906_ALERT_3_C Large K Value in the Analysis of Variance ......     29.868 Check
PLAT906_ALERT_3_C Large K Value in the Analysis of Variance ......      4.883 Check
PLAT906_ALERT_3_C Large K Value in the Analysis of Variance ......      2.164 Check
```

Alert level G

```
PLAT883_ALERT_1_G No Info/Value for _atom_sites_solution_primary .     Please Do !
PLAT910_ALERT_3_G Missing # of FCF Reflection(s) Below Theta(Min).          2 Note
PLAT941_ALERT_3_G Average HKL Measurement Multiplicity ...........        3.2 Low
PLAT965_ALERT_2_G The SHELXL WEIGHT Optimisation has not Converged     Please Check
PLAT967_ALERT_5_G Note: Two-Theta Cutoff Value in Embedded .res ..       53.0 Degree
PLAT978_ALERT_2_G Number C-C Bonds with Positive Residual Density.          7 Info
```

0 **ALERT level A** = Most likely a serious problem - resolve or explain
0 **ALERT level B** = A potentially serious problem, consider carefully
4 **ALERT level C** = Check. Ensure it is not caused by an omission or oversight
6 **ALERT level G** = General information/check it is not something unexpected

1 ALERT type 1 CIF construction/syntax error, inconsistent or missing data
2 ALERT type 2 Indicator that the structure model may be wrong or deficient
6 ALERT type 3 Indicator that the structure quality may be low
0 ALERT type 4 Improvement, methodology, query or suggestion
1 ALERT type 5 Informative message, check

It is advisable to attempt to resolve as many as possible of the alerts in all categories. Often the minor alerts point to easily fixed oversights, errors and omissions in your CIF or refinement strategy, so attention to these fine details can be worthwhile. In order to resolve some of the more serious problems it may be necessary to carry out additional measurements or structure refinements. However, the purpose of your study may justify the reported deviations and the more serious of these should normally be commented upon in the discussion or experimental section of a paper or in the "special_details" fields of the CIF. checkCIF was carefully designed to identify outliers and unusual parameters, but every test has its limitations and alerts that are not important in a particular case may appear. Conversely, the absence of alerts does not guarantee there are no aspects of the results needing attention. It is up to the individual to critically assess their own results and, if necessary, seek expert advice.

Publication of your CIF in IUCr journals

A basic structural check has been run on your CIF. These basic checks will be run on all CIFs submitted for publication in IUCr journals (*Acta Crystallographica*, *Journal of Applied Crystallography*, *Journal of Synchrotron Radiation*); however, if you intend to submit to *Acta Crystallographica Section C* or *E* or *IUCrData*, you should make sure that full publication checks are run on the final version of your CIF prior to submission.

Publication of your CIF in other journals

Please refer to the *Notes for Authors* of the relevant journal for any special instructions relating to CIF submission.

PLATON version of 28/11/2022; check.def file version of 28/11/2022

Datablock B9_S9_exp63_deposit_new - ellipsoid plot

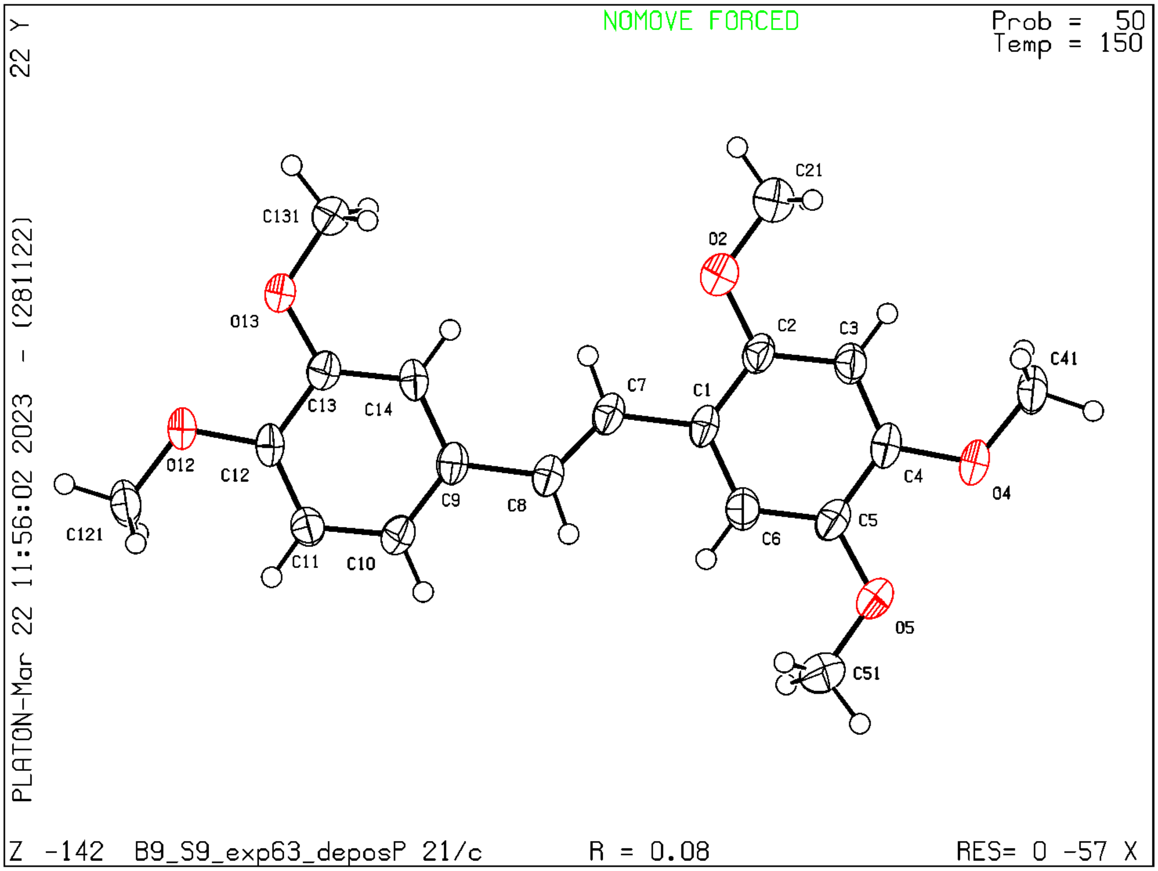

Supplement: Supplementary file 1 [file ijms-24-07200-s001.zip › checkcif_MTS9.pdf]
