# Supplementary material for: Systematic Study of Solid-State Fluorescence and Molecular Packing of Methoxy-trans-Stilbene Derivatives, Exploration of Weak Intermolecular Interactions Based on Hirshfeld Surface Analysis
Source: Int J Mol Sci. 2023 Apr 13;24(8):7200. doi: 10.3390/ijms24087200 (PMC10139126; doi:10.3390/ijms24087200)
Supplement: Supplementary file 1 [file ijms-24-07200-s001.zip › Supplementary file.pdf]

Supplementary material

Systematic study of solid-state fluorescence and molecular packing of methoxy *trans*-stilbene derivatives. Exploration of weak intermolecular interactions based on Hirshfeld surface analysis.

Natalia Piekuś-Słomka [1,*], Magdalena Małecka [2], Marcin Wierzchowski [3], Bogumiła Kupcewicz [1,*]

[1] Department of Inorganic and Analytical Chemistry, Nicolaus Copernicus University in Toruń, Ludwik Rydygier Collegium Medicum in Bydgoszcz, Jurasza 2, Bydgoszcz, Poland; kizchemanal@cm.umk.pl
[2] Department of Physical Chemistry, Faculty of Chemistry, University of Łódź, Pomorska 163/165, Łódź, Poland; magdalena.malecka@chemia.uni.lodz.pl
[3] Department of Chemical Technology of Drugs, Poznań University of Medical Sciences, Fredry 10, Poznań, Poland; mwierzch@ump.edu.pl

* Correspondence: natalia.piekus@cm.umk.pl (N.P.-S.); kupcewicz@cm.umk.pl (B.K.)

1. Supplementary Figures

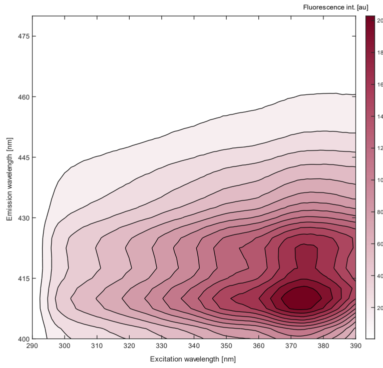

MTS-4

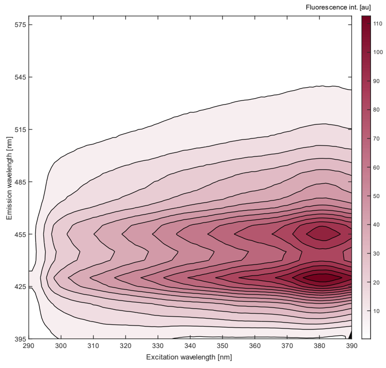

MTS-12

Figure S1. Exemplary top view of three-dimensional solid-state excitation–emission fluorescence spectra of **MTS-4** and **MTS-12**.

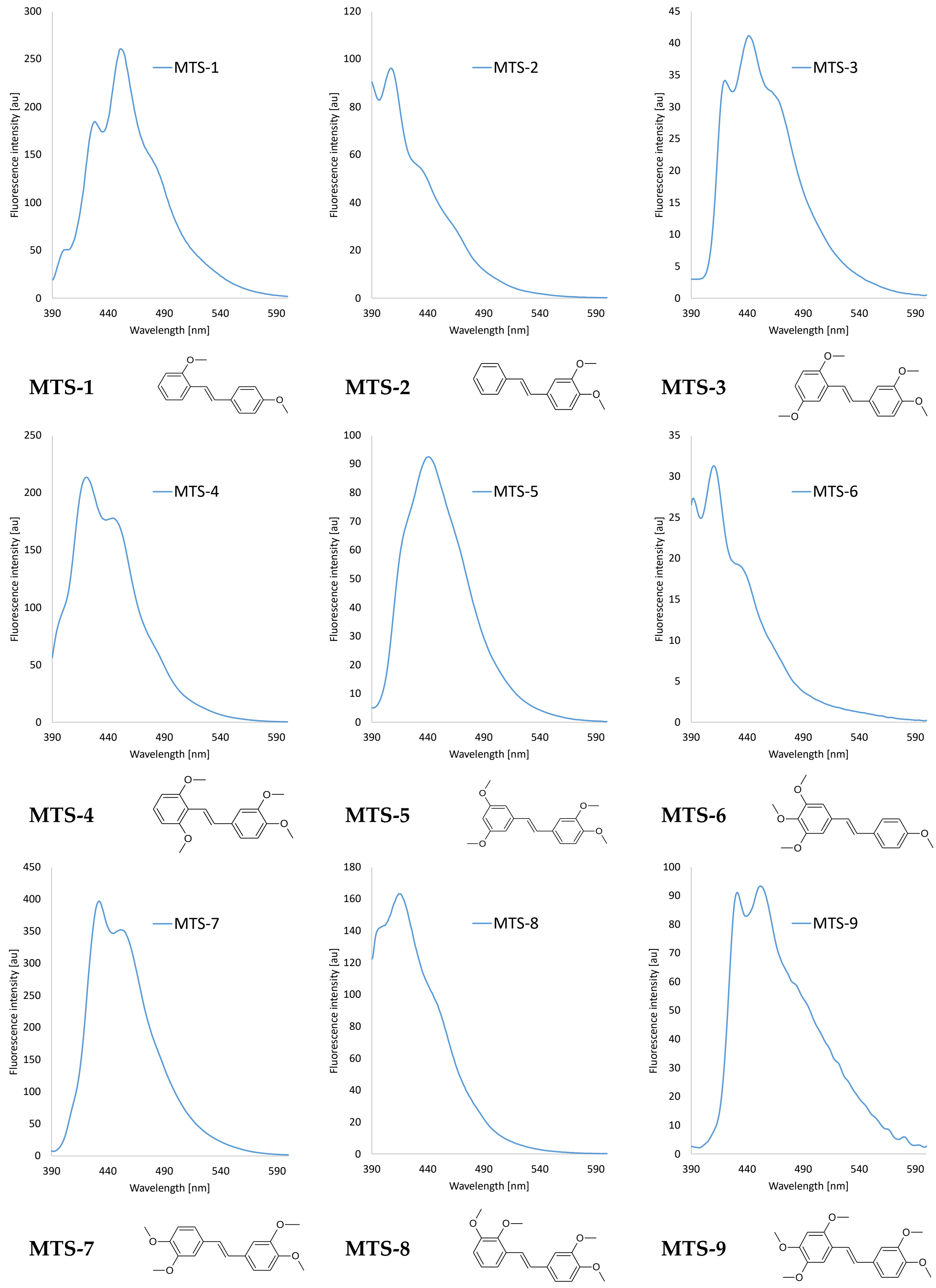

Figure S2. Solid-state fluorescence emission spectra of studied compounds (excited by maximum absorption wavelength).

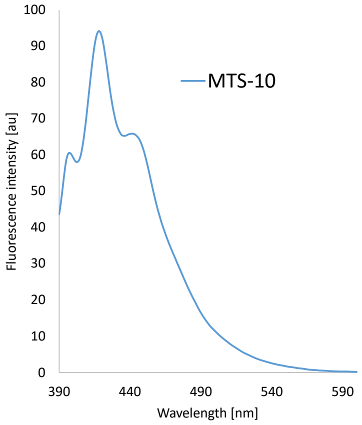

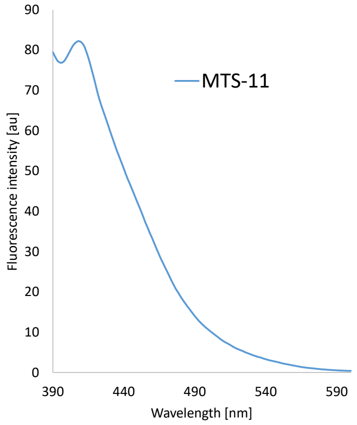

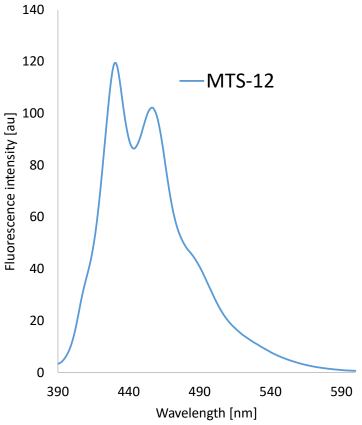

MTS-10

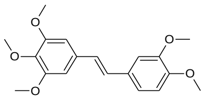

MTS-11

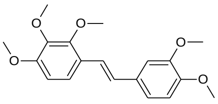

MTS-12

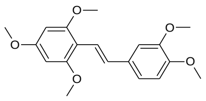

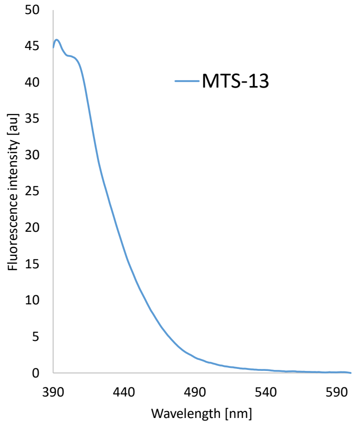

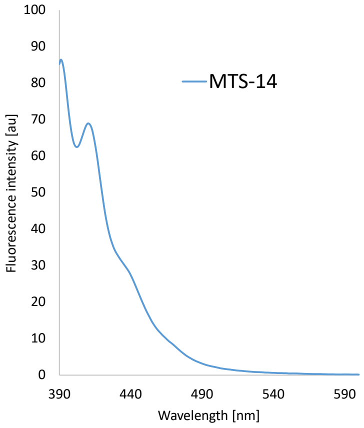

MTS-13

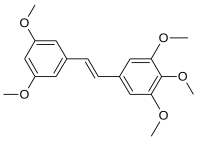

MTS-14

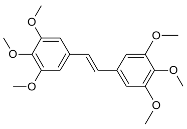

Figure S2 (cont.). Solid-state fluorescence emission spectra of studied compounds (excited by maximum absorption wavelength).

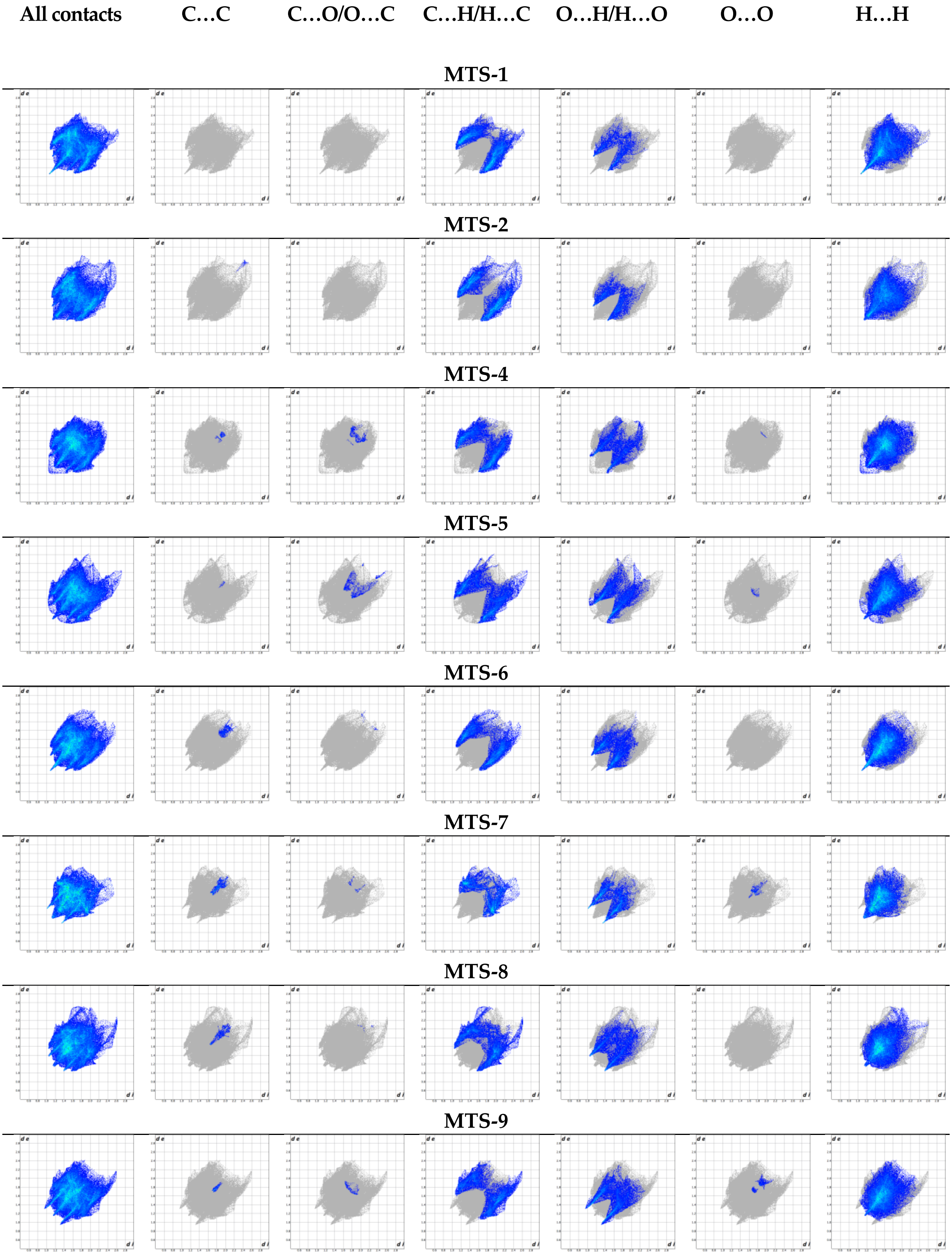

Figure S3. 2D fingerprint plots of particular interactions observed in the crystal structure of studied compounds.

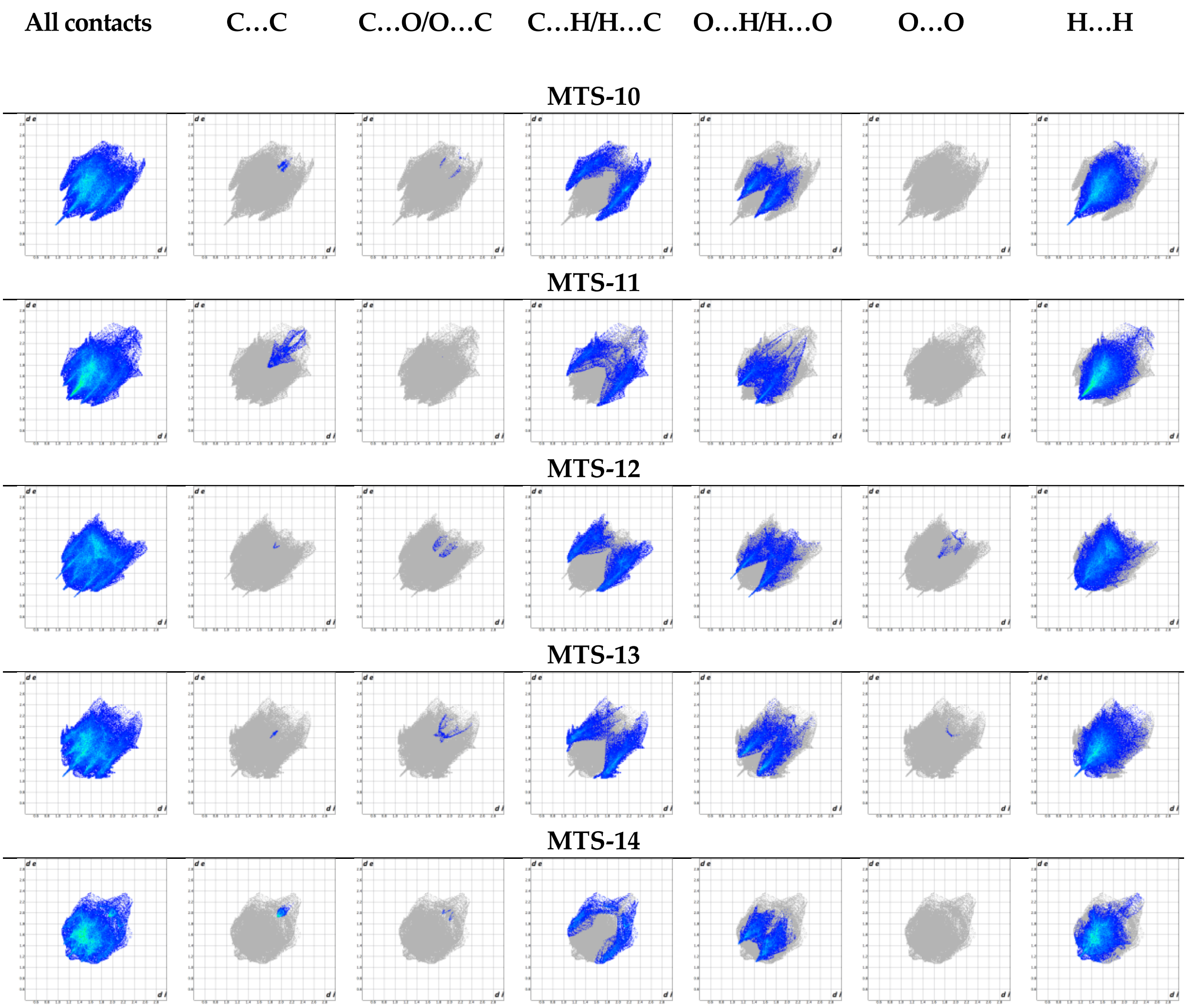

Figure S3 (cont.). 2D fingerprint plots of particular interactions observed in the crystal structure of studied compounds.

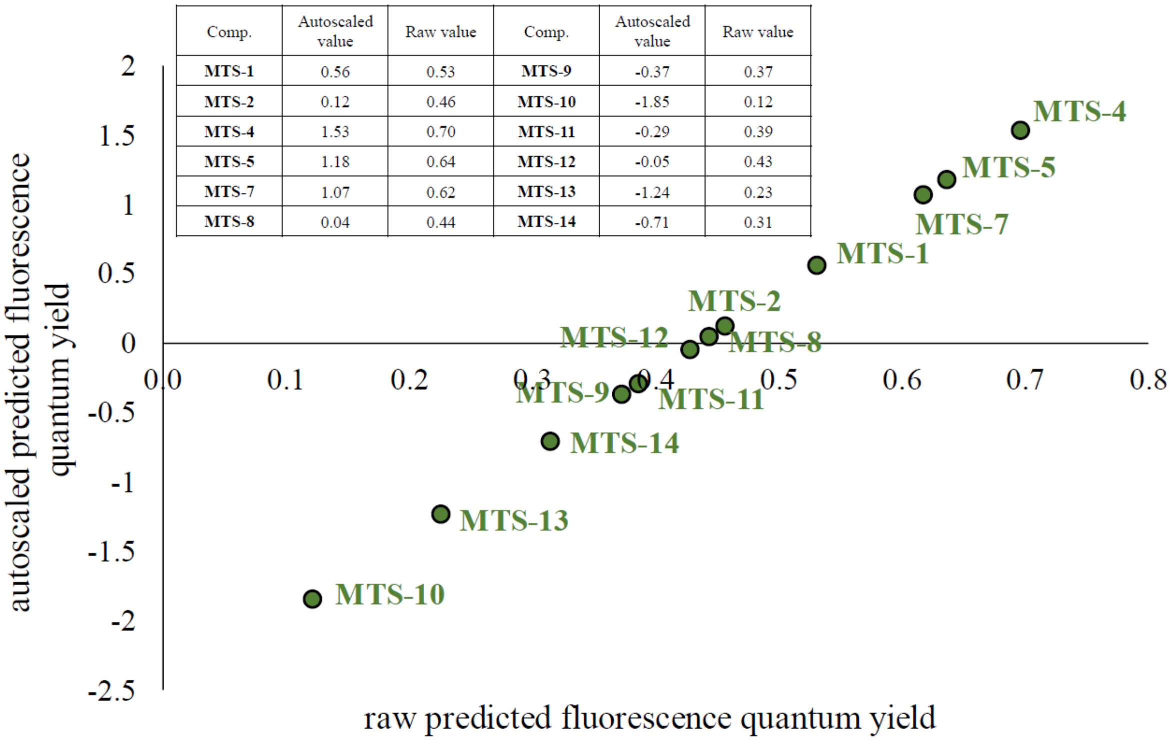

Comp.	Autoscaled value	Raw value	Comp.	Autoscaled value	Raw value
**MTS-1**	0.56	0.53	**MTS-9**	-0.37	0.37
**MTS-2**	0.12	0.46	**MTS-10**	-1.85	0.12
**MTS-4**	1.53	0.70	**MTS-11**	-0.29	0.39
**MTS-5**	1.18	0.64	**MTS-12**	-0.05	0.43
**MTS-7**	1.07	0.62	**MTS-13**	-1.24	0.23
**MTS-8**	0.04	0.44	**MTS-14**	-0.71	0.31

Figure S4. The relationship between raw and autoscaled values of predicted fluorescence quantum yield.

2. Supplementary Tables

Table S1. Details of fluorescence lifetime measurements.

Compound	Fluorescence lifetime ± SD [ns], (fractional amplitudes [%])		X^2
	τ_1	τ_2	
MTS-1	1.80 ± 0.002 (100%)	–	1.009
MTS-2	1.92 ± 0.002 (100%)	–	1.011
MTS-3	1.37 ± 0.002 (100%)	–	1.001
MTS-4	2.01 ± 0.003 (100%)	–	1.002
MTS-5	2.68 ± 0.060 (49%)	4.20 ± 0.078 (51%)	1.047
MTS-6	0.83 ± 0.003 (100%)	–	1.026
MTS-7	1.06 ± 0.016 (37%)	2.34 ± 0.016 (63%)	1.018
MTS-8	2.62 ± 0.003 (100%)	–	1.011
MTS-9	0.34 ± 0.003 (63%)	1.63 ± 0.008 (37%)	1.001
MTS-10	0.88 ± 0.006 (65%)	2.64 ± 0.027 (35%)	1.018
MTS-11	1.12 ± 0.015 (74%)	2.01 ± 0.057 (26%)	1.021
MTS-12	1.04 ± 0.021 (70%)	1.82 ± 0.070 (30%)	0.911
MTS-13	1.51 ± 0.003 (100%)	–	1.063
MTS-14	1.39 ± 0.003 (100%)	–	1.030

MTS-8

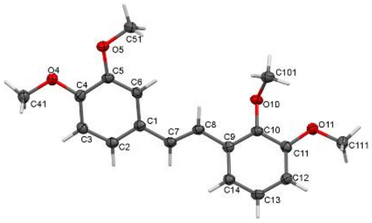

Table S2. Bond lengths [Å] for **MTS-8**.

Bond	Distance [Å]	Bond	Distance [Å]
O4-C4	1.3717(1)	C13-C14	1.3775(1)
O4-C41	1.4249(1)	C2-H2	0.9500
O5-C5	1.3723(1)	C3-H3	0.9500
O5-C51	1.4283(1)	C6-H6	0.9500
O10-C10	1.3826(1)	C7-H7	0.9500
O10-C101	1.4377(1)	C8-H8	0.9500
O11-C11	1.3667(1)	C12-H12	0.9500
O11-C111	1.4291(1)	C13-H13	0.9500
C1-C2	1.3825(1)	C14-H14	0.9500
C1-C6	1.4112(1)	C41-H4A	0.9800
C1-C7	1.4678(1)	C41-H4B	0.9800
C2-C3	1.4004(1)	C41-H4C	0.9800
C3-C4	1.3840(1)	C51-H5A	0.9800
C4-C5	1.4085(1)	C51-H5B	0.9800
C5-C6	1.3757(1)	C51-H5C	0.9800
C7-C8	1.3319(1)	C101-H10A	0.9800
C8-C9	1.4657(1)	C101-H10B	0.9800
C9-C10	1.3977(1)	C101-H10C	0.9800
C9-C14	1.4073(1)	C111-H11A	0.9800
C10-C11	1.4026(1)	C111-H11B	0.9800
C11-C12	1.3897(1)	C111-H11C	0.9800
C12-C13	1.3889(1)		

Table S3. Bond angles [°] for **MTS-8**.

**Bond angles**	**Degree**	**Bond angles**	**Degree**
C4-O4-C41	117.20(1)	C5-C6-H6	119.00
C5-O5-C51	116.64(1)	C1-C7-H7	117.00
C10-O10-C101	115.29(1)	C8-C7-H7	117.00
C11-O11-C111	117.27(1)	C7-C8-H8	117.00
C2-C1-C6	117.89(1)	C9-C8-H8	117.00
C2-C1-C7	119.71(1)	C11-C12-H12	120.00
C6-C1-C7	122.39(1)	C13-C12-H12	121.00
C1-C2-C3	121.77(1)	C12-C13-H13	119.00
C2-C3-C4	119.60(1)	C14-C13-H13	119.00
O4-C4-C3	124.86(1)	C9-C14-H14	120.00
O4-C4-C5	115.67(1)	C13-C14-H14	120.00
C3-C4-C5	119.47(1)	O4-C41-H4A	109.00
O5-C5-C4	114.66(1)	O4-C41-H4B	109.00
O5-C5-C6	125.16(1)	O4-C41-H4C	110.00
C4-C5-C6	120.15(1)	H4A-C41-H4B	109.00
C1-C6-C5	121.10(1)	H4A-C41-H4C	110.00
C1-C7-C8	126.76(1)	H4B-C41-H4C	109.00
C7-C8-C9	126.05(1)	O5-C51-H5A	109.00
C8-C9-C10	120.16(1)	O5-C51-H5B	109.00
C8-C9-C14	121.90(1)	O5-C51-H5C	109.00
C10-C9-C14	117.87(1)	H5A-C51-H5B	109.00
O10-C10-C9	118.42(1)	H5A-C51-H5C	110.00
O10-C10-C11	120.25(1)	H5B-C51-H5C	110.00
C9-C10-C11	121.18(1)	O10-C101-H10A	110.00
O11-C11-C10	115.71(1)	O10-C101-H10B	109.00
O11-C11-C12	124.38(1)	O10-C101-H10C	110.00
C10-C11-C12	119.90(1)	H10A-C101-H10B	109.00
C11-C12-C13	118.99(1)	H10A-C101-H10C	109.00
C12-C13-C14	121.48(1)	H10B-C101-H10C	109.00
C9-C14-C13	120.58(1)	O11-C111-H11A	109.00
C1-C2-H2	119.00	O11-C111-H11B	109.00
C3-C2-H2	119.00	O11-C111-H11C	109.00
C2-C3-H3	120.00	H11A-C111-H11B	110.00
C4-C3-H3	120.00	H11A-C111-H11C	110.00
C1-C6-H6	119.00	H11B-C111-H11C	109.00

Table S4. Torsion angles [°] for **MTS-8**.

Torsion angles	Degree	Torsion angles	Degree
C41-O4-C4-C3	1.26	C3-C4-C5-C6	-1.77
C41-O4-C4-C5	-178.62	O5-C5-C6-C1	178.76
C51-O5-C5-C4	-179.11	C4-C5-C6-C1	0.85
C51-O5-C5-C6	2.88	C1-C7-C8-C9	174.88
C101-O10-C10-C9	114.65	C7-C8-C9-C10	159.63
C101-O10-C10-C11	-69.86	C7-C8-C9-C14	-23.37
C111-O11-C11-C10	172.03	C8-C9-C10-O10	-7.38
C111-O11-C11-C12	-9.50	C8-C9-C10-C11	177.18
C6-C1-C2-C3	-1.53	C14-C9-C10-O10	175.50
C7-C1-C2-C3	177.39	C14-C9-C10-C11	0.06
C2-C1-C6-C5	0.79	C8-C9-C14-C13	-177.20
C7-C1-C6-C5	-178.10	C10-C9-C14-C13	-0.13
C2-C1-C7-C8	-177.41	O10-C10-C11-O11	3.31
C6-C1-C7-C8	1.46	O10-C10-C11-C12	-175.24
C1-C2-C3-C4	0.63	C9-C10-C11-O11	178.67
C2-C3-C4-O4	-178.84	C9-C10-C11-C12	0.12
C2-C3-C4-C5	1.03	O11-C11-C12-C13	-178.64
O4-C4-C5-O5	0.00	C10-C11-C12-C13	-0.23
O4-C4-C5-C6	178.12	C11-C12-C13-C14	0.15
C3-C4-C5-O5	-179.89	C12-C13-C14-C9	0.03

MTS-9

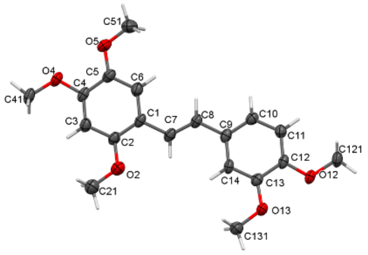

Table S5. Bond lengths [Å] for **MTS-9**.

Bond	Distance [Å]	Bond	Distance [Å]
O2 -C2	1.3459(1)	C13-C14	1.3844(1)
O2-C21	1.4075(1)	C3 -H3	0.9300
O4-C4	1.3755(1)	C6 -H6	0.9300
O4-C41	1.4264(1)	C7 -H7	0.9300
O5-C5	1.3610(1)	C8 -H8	0.9300
O5-C51	1.4116(1)	C10-H10	0.9300
O12-C12	1.3642(1)	C11-H11	0.9300
O12-C121	1.4367(1)	C14-H14	0.9300
O13-C13	1.3623(1)	C21-H2A	0.9600
O13-C131	1.4149(1)	C21-H2B	0.9600
C1-C2	1.3860(1)	C21-H2C	0.9600
C1-C6	1.3933(1)	C41-H41A	0.9600
C1-C7	1.4754(1)	C41-H41B	0.9600
C2-C3	1.4098(1)	C41-H41C	0.9600
C3-C4	1.3658(1)	C51-H5A	0.9600
C4-C5	1.3979(1)	C51-H5B	0.9600
C5-C6	1.3874(1)	C51-H5C	0.9600
C7-C8	1.3258(1)	C121-H12A	0.9600
C8-C9	1.4739(1)	C121-H12B	0.9600
C9-C10	1.3774(1)	C121-H12C	0.9600
C9-C14	1.4063(1)	C131-H13A	0.9600
C10-C11	1.3895(1)	C131-H13B	0.9600
C11-C12	1.3741(1)	C131-H13C	0.9600
C12-C13	1.4137(1)		

Table S6. Bond angles [°] for **MTS-9**.

Bond angles	Degree	Bond angles	Degree
C2-O2-C21	117.64(1)	C7-C8-H8	117.00
C4-O4-C41	117.55(1)	C9-C8-H8	117.00
C5-O5-C51	116.66(1)	C9-C10-H10	119.00
C12-O12-C121	116.52(1)	C11-C10-H10	119.00
C13-O13-C131	117.69(1)	C10-C11-H11	120.00
C2-C1-C6	118.41(1)	C12-C11-H11	120.00
C2-C1-C7	119.55(1)	C9-C14-H14	120.00
C6-C1-C7	122.05(1)	C13-C14-H14	120.00
O2-C2-C1	116.86(1)	O2-C21-H2A	109.00
O2-C2-C3	122.59(1)	O2-C21-H2B	110.00
C1-C2-C3	120.52(1)	O2-C21-H2C	110.00
C2-C3-C4	119.68(1)	H2A-C21-H2B	109.00
O4-C4-C3	124.35(1)	H2A-C21-H2C	109.00
O4-C4-C5	114.61(1)	H2B-C21-H2C	110.00
C3-C4-C5	121.03(1)	O4-C41-H41A	109.00
O5-C5-C4	116.41(1)	O4-C41-H41B	109.00
O5-C5-C6	125.07(1)	O4-C41-H41C	109.00
C4-C5-C6	118.52(1)	H41A-C41-H41B	109.00
C1-C6-C5	121.83(1)	H41A-C41-H41C	110.00
C1-C7-C8	126.95(1)	H41B-C41-H41C	109.00
C7-C8-C9	126.76(1)	O5-C51-H5A	109.00
C8-C9-C10	119.73(1)	O5-C51-H5B	110.00
C8-C9-C14	122.21(1)	O5-C51-H5C	109.00
C10-C9-C14	118.06(1)	H5A-C51-H5B	109.00
C9-C10-C11	122.03(1)	H5A-C51-H5C	109.00
C10-C11-C12	119.81(1)	H5B-C51-H5C	110.00
O12-C12-C11	125.28(1)	O12-C121-H12A	109.00
O12-C12-C13	115.09(1)	O12-C121-H12B	109.00
C11-C12-C13	119.63(1)	O12-C121-H12C	109.00
O13-C13-C12	115.20(1)	H12A-C121-H12B	109.00
O13-C13-C14	125.19(1)	H12A-C121-H12C	109.00
C12-C13-C14	119.61(1)	H12B-C121-H12C	109.00
C9-C14-C13	120.83(1)	O13-C131-H13A	109.00
C2-C3-H3	120.00	O13-C131-H13B	109.00
C4-C3-H3	120.00	O13-C131-H13C	109.00
C1-C6-H6	119.00	H13A-C131-H13B	110.00
C5-C6-H6	119.00	H13A-C131-H13C	110.00
C1-C7-H7	117.00	H13B-C131-H13C	109.00
C8-C7-H7	117.00		

Table S7. Torsion angles [°] for **MTS-9**.

Torsion angles	Degree	Torsion angles	Degree
C21-O2-C2-C1	-179.67(1)	O4-C4-C5-O5	1.38(1)
C21-O2-C2-C3	2.54(1)	O4-C4-C5-C6	-178.75(1)
C41-O4-C4-C3	8.18(1)	C3-C4-C5-O5	-179.44(1)
C41-O4-C4-C5	-172.67(1)	C3-C4-C5-C6	0.43(1)
C51-O5-C5-C4	171.48(1)	O5-C5-C6-C1	179.72(1)
C51-O5-C5-C6	-8.38(1)	C4-C5-C6-C1	-0.14(1)
C121-O12-C12-C11	0.35(1)	C1-C7-C8-C9	179.61(1)
C121-O12-C12-C13	-179.81(1)	C7-C8-C9-C10	167.79(1)
C131-O13-C13-C12	179.84(1)	C7-C8-C9-C14	-12.62(1)
C131-O13-C13-C14	0.58(1)	C8-C9-C10-C11	-178.11(1)
C6-C1-C2-O2	-177.50(1)	C14-C9-C10-C11	2.28(1)
C6-C1-C2-C3	0.35(1)	C8-C9-C14-C13	178.96(1)
C7-C1-C2-O2	2.11(1)	C10-C9-C14-C13	-1.44(1)
C7-C1-C2-C3	179.94(1)	C9-C10-C11-C12	-1.42(1)
C2-C1-C6-C5	-0.25(1)	C10-C11-C12-O12	179.52(1)
C7-C1-C6-C5	-179.84(1)	C10-C11-C12-C13	-0.32(1)
C2-C1-C7-C8	-173.92(1)	O12-C12-C13-O13	1.95(1)
C6-C1-C7-C8	5.67(1)	O12-C12-C13-C14	-178.74(1)
O2-C2-C3-C4	177.66(1)	C11-C12-C13-O13	-178.20(1)
C1-C2-C3-C4	-0.07(1)	C11-C12-C13-C14	1.11(1)
C2-C3-C4-O4	178.76(1)	O13-C13-C14-C9	179.02(1)
C2-C3-C4-C5	-0.34(1)	C12-C13-C14-C9	-0.21(1)

MTS-10

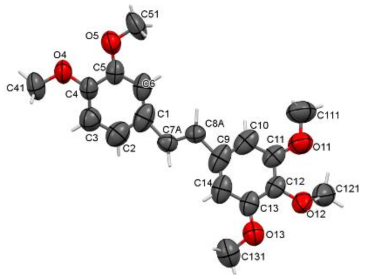

Table S8. Bond lengths [Å] for **MTS-10**.

Bond	Distance [Å]	Bond	Distance [Å]
O4-C4	1.3618(1)	C12-C13	1.3900(1)
O4-C41	1.4296(1)	C13-C14	1.3900(1)
O5-C5	1.3507(1)	C2-H2	0.9300
O5-C51	1.4087(1)	C3-H3	0.9300
O11-C11	1.3570(1)	C6-H6	0.9300
O11-C111	1.4099(1)	C7A-H7B	1.0200
O12-C12	1.3765(1)	C7B-H7A	1.0500
O12-C121	1.4150(1)	C8A-H7A	1.0700
O13-C13	1.3636(1)	C8B-H7B	0.9100
O13-C131	1.4205(1)	C10-H10	0.9300
C1-C2	1.3900(1)	C14-H14	0.9300
C1-C6	1.3900(1)	C41-H4C	0.9600
C1-C7A	1.5301(1)	C41-H4B	0.9600
C1-C7B	1.5463(1)	C41-H4A	0.9600
C2-C3	1.3900(1)	C51-H5B	0.9600
C3-C4	1.3900(1)	C51-H5A	0.9600
C4-C5	1.3900(1)	C51-H5C	0.9600
C5-C6	1.3900(1)	C111-H11B	0.9600
C7A-C7B	0.9619(1)	C111-H11A	0.9600
C8A-C8B	0.9527(1)	C111-H11C	0.9600
C8A-C9	1.5479(1)	C121-H12B	0.9600
C8B-C9	1.5555(1)	C121-H12C	0.9600
C9-C14	1.3900(1)	C121-H12A	0.9600
C9-C10	1.3901(1)	C131-H13C	0.9600
C10-C11	1.3900(1)	C131-H13A	0.9600
C11-C12	1.3899(1)	C131-H13B	0.9600

Table S9. Bond angles [°] for **MTS-10**.

Bond angles	Degree	Bond angles	Degree
C4-O4-C41	118.00(1)	C1-C6-H6	120.00
C5-O5-C51	117.32(1)	C5-C6-H6	120.00
C11-O11-C111	118.56(1)	C1-C7A-H7B	142.00
C12-O12-C121	116.34(1)	C7B-C7A-H7B	145.00
C13-O13-C131	118.17(1)	C1-C7B-H7A	132.00
C2-C1-C6	120.00(1)	C7A-C7B-H7A	157.00
C2-C1-C7A	101.79(1)	C8B-C8A-H7A	155.00
C2-C1-C7B	137.33(1)	C9-C8A-H7A	132.00
C6-C1-C7A	137.92(1)	C8A-C8B-H7B	157.00
C6-C1-C7B	102.57(1)	C9-C8B-H7B	131.00
C7A-C1-C7B	36.43(1)	C11-C10-H10	120.00
C1-C2-C3	120.00(1)	C9-C10-H10	120.00
C2-C3-C4	120.01(1)	C9-C14-H14	120.00
O4-C4-C3	124.96(1)	C13-C14-H14	120.00
O4-C4-C5	115.04(1)	H4A-C41-H4B	109.00
C3-C4-C5	120.00(1)	H4A-C41-H4C	109.00
O5-C5-C4	115.26(1)	O4-C41-H4A	109.00
O5-C5-C6	124.73(1)	O4-C41-H4B	109.00
C4-C5-C6	120.00(1)	H4B-C41-H4C	109.00
C1-C6-C5	120.01(1)	O4-C41-H4C	109.00
C1-C7A-C7B	72.70(1)	O5-C51-H5A	109.00
C1-C7B-C7A	70.86(1)	O5-C51-H5B	109.00
C8B-C8A-C9	72.56(1)	O5-C51-H5C	109.00
C8A-C8B-C9	71.69(1)	H5A-C51-H5B	109.00
C8A-C9-C10	101.76(1)	H5A-C51-H5C	109.00
C8A-C9-C8B	35.75(1)	H5B-C51-H5C	109.00
C8B-C9-C14	102.79(1)	O11-C111-H11B	109.00
C10-C9-C14	119.99(1)	O11-C111-H11C	109.00
C8A-C9-C14	138.24(1)	O11-C111-H11A	109.00
C8B-C9-C10	137.06(1)	H11B-C111-H11C	109.00
C9-C10-C11	120.00(1)	H11A-C111-H11B	109.00
C10-C11-C12	120.00(1)	H11A-C111-H11C	109.00
O11-C11-C10	125.35(1)	O12-C121-H12B	109.00
O11-C11-C12	114.30(1)	O12-C121-H12C	109.00
C11-C12-C13	120.01(1)	H12A-C121-H12B	109.00
O12-C12-C11	122.17(1)	H12A-C121-H12C	109.00
O12-C12-C13	117.70(1)	O12-C121-H12A	109.00
O13-C13-C12	115.39(1)	H12B-C121-H12C	109.00
O13-C13-C14	124.61(1)	H13A-C131-H13C	109.00
C12-C13-C14	119.99(1)	H13B-C131-H13C	109.00
C9-C14-C13	120.01(1)	H13A-C131-H13B	109.00
C1-C2-H2	120.00	O13-C131-H13A	109.00
C3-C2-H2	120.00	O13-C131-H13B	109.00
C2-C3-H3	120.00	O13-C131-H13C	109.00
C4-C3-H3	120.00		

Table S10. Torsion angles [°] for **MTS-10**.

Torsion angles	Degree	Torsion angles	Degree
C41-O4-C4-C3	-7.55	C8B-C8A-C9-C14	-9.35
C41-O4-C4-C5	171.92	C8A-C8B-C9-C10	-11.27
C51-O5-C5-C4	172.07	C8A-C8B-C9-C14	173.63
C51-O5-C5-C6	-6.43	C8A-C9-C10-C11	178.83
C111-O11-C11-C10	7.86	C8B-C9-C10-C11	-174.47
C111-O11-C11-C12	-178.92	C14-C9-C10-C11	0.02
C121-O12-C12-C11	64.94	C8A-C9-C14-C13	-178.28
C121-O12-C12-C13	-119.03	C8B-C9-C14-C13	176.14
C131-O13-C13-C12	-163.72	C10-C9-C14-C13	-0.02
C131-O13-C13-C14	17.53	C9-C10-C11-O11	172.87
C6-C1-C2-C3	0.00	C9-C10-C11-C12	0.00
C2-C1-C6-C5	0.02	O11-C11-C12-O12	2.32
C1-C2-C3-C4	0.00	O11-C11-C12-C13	-173.62
C2-C3-C4-O4	179.44	C10-C11-C12-O12	175.94
C2-C3-C4-C5	0.00	C10-C11-C12-C13	0.00
O4-C4-C5-O5	1.93	O12-C12-C13-O13	5.07
O4-C4-C5-C6	-179.49	O12-C12-C13-C14	-176.12
C3-C4-C5-O5	-178.57	C11-C12-C13-O13	-178.82
C3-C4-C5-C6	0.00	C11-C12-C13-C14	0.00
O5-C5-C6-C1	178.43	O13-C13-C14-C9	178.70
C4-C5-C6-C1	-0.03	C12-C13-C14-C9	0.00
C8B-C8A-C9-C10	172.18		

MTS-13

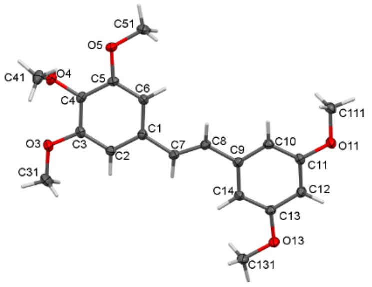

Table S11. Bond lengths [Å] for **MTS-13**.

Bond	Distance [Å]	Bond	Distance [Å]
O3-C3	1.3675(1)	C13-C14	1.3897(1)
O3-C31	1.4262(1)	C2-H2	0.9300
O4-C4	1.3740(1)	C6-H6	0.9300
O4-C41	1.4256(1)	C7-H7	0.9300
O5-C5	1.3641(1)	C8-H8	0.9300
O5-C51	1.4279(1)	C10-H10	0.9300
O11-C11	1.3710(1)	C12-H12	0.9300
O11-C111	1.4277(1)	C14-H14	0.9300
O13-C13	1.3683(1)	C31-H3A	0.9600
O13-C131	1.4280(1)	C31-H3B	0.9600
C1-C2	1.4003(1)	C31-H3C	0.9600
C1-C6	1.3979(1)	C41-H4A	0.9600
C1-C7	1.4673(1)	C41-H4B	0.9600
C2-C3	1.3934(1)	C41-H4C	0.9600
C3-C4	1.3965(1)	C51-H5A	0.9600
C4-C5	1.4005(1)	C51-H5B	0.9600
C5-C6	1.3869(1)	C51-H5C	0.9600
C7-C8	1.3343(1)	C111-H11A	0.9600
C8-C9	1.4688(1)	C111-H11B	0.9600
C9-C10	1.3983(1)	C111-H11C	0.9600
C9-C14	1.3982(1)	C131-H13A	0.9600
C10-C11	1.3931(1)	C131-H13B	0.9600
C11-C12	1.3874(1)	C131-H13C	0.9600
C12-C13	1.3929(1)		

Table S12. Bond angles [°] for **MTS-13**.

Bond angles	Degree	Bond angles	Degree
C3-O3-C31	116.83(1)	C7-C8-H8	117.00
C4-O4-C41	112.69(1)	C9-C8-H8	117.00
C5-O5-C51	116.76(1)	C9-C10-H10	120.00
C11-O11-C111	116.95(1)	C11-C10-H10	120.00
C13-O13-C131	116.22(1)	C11-C12-H12	120.00
C2-C1-C6	120.00(1)	C13-C12-H12	120.00
C2-C1-C7	118.74(1)	C9-C14-H14	120.00
C6-C1-C7	121.23(1)	C13-C14-H14	120.00
C1-C2-C3	119.84(1)	O3-C31-H3A	109.00
O3-C3-C2	124.38(1)	O3-C31-H3B	109.00
O3-C3-C4	115.33(1)	O3-C31-H3C	109.00
C2-C3-C4	120.29(1)	H3A-C31-H3B	109.00
O4-C4-C3	120.75(1)	H3A-C31-H3C	109.00
O4-C4-C5	119.73(1)	H3B-C31-H3C	109.00
C3-C4-C5	119.46(1)	O4-C41-H4A	109.00
O5-C5-C4	114.93(1)	O4-C41-H4B	109.00
O5-C5-C6	124.50(1)	O4-C41-H4C	109.00
C4-C5-C6	120.56(1)	H4A-C41-H4B	109.00
C1-C6-C5	119.84(1)	H4A-C41-H4C	109.00
C1-C7-C8	125.43(1)	H4B-C41-H4C	109.00
C7-C8-C9	126.53(1)	O5-C51-H5A	109.00
C8-C9-C10	117.70(1)	O5-C51-H5B	109.00
C8-C9-C14	122.21(1)	O5-C51-H5C	109.00
C10-C9-C14	120.01(1)	H5A-C51-H5B	109.00
C9-C10-C11	119.47(1)	H5A-C51-H5C	109.00
O11-C11-C10	123.59(1)	H5B-C51-H5C	109.00
O11-C11-C12	115.49(1)	O11-C111-H11A	109.00
C10-C11-C12	120.92(1)	O11-C111-H11B	109.00
C11-C12-C13	119.18(1)	O11-C111-H11C	109.00
O13-C13-C12	115.67(1)	H11A-C111-H11B	109.00
O13-C13-C14	123.43(1)	H11A-C111-H11C	109.00
C12-C13-C14	120.90(1)	H11B-C111-H11C	109.00
C9-C14-C13	119.52(1)	O13-C131-H13A	109.00
C1-C2-H2	120.00	O13-C131-H13B	109.00
C3-C2-H2	120.00	O13-C131-H13C	109.00
C1-C6-H6	120.00	H13A-C131-H13B	109.00
C5-C6-H6	120.00	H13A-C131-H13C	109.00
C1-C7-H7	117.00	H13B-C131-H13C	109.00
C8-C7-H7	117.00		

Table S13. Torsion angles [°] for **MTS-13**.

Torsion angles	Degree	Torsion angles	Degree
C31-O3-C3-C2	-5.15(1)	O4-C4-C5-O5	1.33(1)
C31-O3-C3-C4	175.02(1)	O4-C4-C5-C6	-177.67(1)
C41-O4-C4-C3	93.83(1)	C3-C4-C5-O5	178.70(1)
C41-O4-C4-C5	-88.84(1)	C3-C4-C5-C6	-0.30(1)
C51-O5-C5-C4	-177.22(1)	O5-C5-C6-C1	-179.50(1)
C51-O5-C5-C6	1.73(1)	C4-C5-C6-C1	-0.60(1)
C111-O11-C11-C10	-3.74(1)	C1-C7-C8-C9	-172.21(1)
C111-O11-C11-C12	175.50(1)	C7-C8-C9-C10	165.66(1)
C131-O13-C13-C12	-171.76(1)	C7-C8-C9-C14	-11.08(1)
C131-O13-C13-C14	7.70(1)	C8-C9-C10-C11	-175.71(1)
C6-C1-C2-C3	-1.36(1)	C14-C9-C10-C11	1.11(1)
C7-C1-C2-C3	-179.45(1)	C8-C9-C14-C13	175.44(1)
C2-C1-C6-C5	1.43(1)	C10-C9-C14-C13	-1.23(1)
C7-C1-C6-C5	179.48(1)	C9-C10-C11-O11	178.39(1)
C2-C1-C7-C8	-169.11(1)	C9-C10-C11-C12	-0.80(1)
C6-C1-C7-C8	12.83(1)	O11-C11-C12-C13	-178.65(1)
C1-C2-C3-O3	-179.37(1)	C10-C11-C12-C13	0.61(1)
C1-C2-C3-C4	0.45(1)	C11-C12-C13-O13	178.74(1)
O3-C3-C4-O4	-2.45(1)	C11-C12-C13-C14	-0.73(1)
O3-C3-C4-C5	-179.79(1)	O13-C13-C14-C9	-178.38(1)
C2-C3-C4-O4	177.72(1)	C12-C13-C14-C9	1.04(1)
C2-C3-C4-C5	0.37(1)		

MTS-14

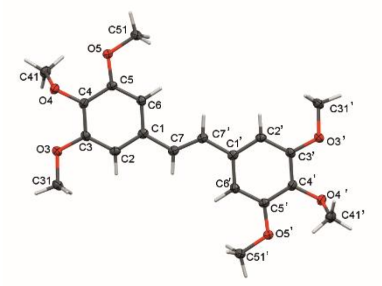

Table S14. Bond lengths [Å] for **MTS-14**.

Bond	Distance [Å]	Bond	Distance [Å]
O3-C3	1.3747(15)	C7-C7_a	1.3386(17)
O3-C31	1.4254(15)	C2-H2	0.9500
O4-C4	1.3765(14)	C6-H6	0.9500
O4-C41	1.4351(16)	C7-H7	0.9500
O5-C5	1.3678(15)	C31-H3A	0.9800
O5-C51	1.4322(15)	C31-H3B	0.9800
C1-C2	1.3980(17)	C31-H3C	0.9800
C1-C6	1.4037(17)	C41-H4A	0.9800
C1-C7	1.4635(17)	C41-H4B	0.9800
C2-C3	1.3906(17)	C41-H4C	0.9800
C3-C4	1.3941(17)	C51-H5A	0.9800
C4-C5	1.4042(17)	C51-H5B	0.9800
C5-C6	1.3846(17)	C51-H5C	0.9800

Table S15. Bond angles [°] for **MTS-14**.

Bond angles	Degree	Bond angles	Degree
C3-O3-C31	116.32(9)	C5-C6-H6	120.00
C4-O4-C41	113.28(9)	C1-C7-H7	117.00
C5-O5-C51	116.48(9)	C7_a-C7-H7	117.00
C2-C1-C6	119.53(11)	O3-C31-H3A	109.00
C2-C1-C7	118.14(11)	O3-C31-H3B	109.00
C6-C1-C7	122.33(11)	O3-C31-H3C	109.00
C1-C2-C3	120.21(11)	H3A-C31-H3B	109.00
O3-C3-C2	124.09(11)	H3A-C31-H3C	109.00
O3-C3-C4	115.37(10)	H3B-C31-H3C	109.00
C2-C3-C4	120.52(11)	O4-C41-H4A	109.00
O4-C4-C3	120.10(11)	O4-C41-H4B	109.00
O4-C4-C5	120.78(11)	O4-C41-H4C	109.00
C3-C4-C5	119.08(11)	H4A-C41-H4B	109.00
O5-C5-C4	115.09(10)	H4A-C41-H4C	109.00
O5-C5-C6	124.17(11)	H4B-C41-H4C	109.00
C4-C5-C6	120.74(11)	O5-C51-H5A	109.00
C1-C6-C5	119.90(11)	O5-C51-H5B	109.00
C1-C7-C7_a	126.71(11)	O5-C51-H5C	109.00
C1-C2-H2	120.00	H5A-C51-H5B	109.00
C3-C2-H2	120.00	H5A-C51-H5C	109.00
C1-C6-H6	120.00	H5B-C51-H5C	109.00

Table S16. Torsion angles [°] for **MTS-14**.

Torsion angles	Degree	Torsion angles	Degree
C31-O3-C3-C2	13.02(18)	C1-C2-C3-C4	-0.8(2)
C31-O3-C3-C4	-168.32(11)	O3-C3-C4-O4	4.77(18)
C41-O4-C4-C3	-102.99(14)	O3-C3-C4-C5	-177.41(11)
C41-O4-C4-C5	79.23(14)	C2-C3-C4-O4	-176.52(12)
C51-O5-C5-C4	-177.08(11)	C2-C3-C4-C5	1.3(2)
C51-O5-C5-C6	3.73(18)	O4-C4-C5-O5	-2.16(18)
C6-C1-C2-C3	-0.3(2)	O4-C4-C5-C6	177.06(12)
C7-C1-C2-C3	179.62(12)	C3-C4-C5-O5	-179.98(10)
C2-C1-C6-C5	0.8(2)	C3-C4-C5-C6	-0.8(2)
C7-C1-C6-C5	-179.07(12)	O5-C5-C6-C1	178.85(12)
C2-C1-C7-C7_a	-179.34(13)	C4-C5-C6-C1	-0.3(2)
C6-C1-C7-C7_a	0.5(2)	C1-C7-C7_a-C1_a	180.00(13)
C1-C2-C3-O3	177.78(12)		

Table S17. Crystallographic data, experimental and refinement details for studied compounds.

Comp.	**MTS-1**	**MTS-2**	**MTS-4**	**MTS-5**	**MTS-6**	**MTS-7**	**MTS-8**
ID CCDC	FIGDOS [1]	CUWZOL [2]	CUXBAA [2]	IXOYEA [3]	REBWAX [4]	QASVOW [5]	2179049
Chemical formula	$C_{16}H_{16}O_2$	$C_{16}H_{16}O_2$	$C_{18}H_{20}O_4$	$C_{18}H_{20}O_4$	$C_{18}H_{20}O_4$	$C_{18}H_{20}O_4$	$C_{18}H_{20}O_4$
Formula weight [g/mol]	240.297	240.297	300.349	300.349	300.349	300.349	300.349
Crystal system	monoclinic	orthorhombic	monoclinic	orthorhombic	orthorhombic	monoclinic	orthorhombic
Space group	P 2_1/c	A ba2	P 2_1/n	P 2_1 2_1 2_1	P na2_1	P 2_1/c	P bca
a, b,c [Å]	18.4720(10)	17.2254(13)	16.0340(3)	5.2431(2)	14.027(2)	8.933(2)	5.2197(2)
	5.6689(2)	24.9655(18)	5.04530(10)	11.9840(7)	13.722(2)	6.7089(15)	23.4997(10)
	13.3386(5)	6.1462(4)	19.5669(4)	25.6315(11)	8.1088(12)	12.416(2)	25.0186(13)
α. β, γ [°]	90.00	90.00	90.00	90.00	90.00	90.00	90.00
	110.403(5)	90.00	95.835(2)	90.00	90.00	92.673(16)	90.0
	90.00	90.00	90.00	90.00	90.00	90.00	90.00
V [Å^3]	1309.14	2643.12	1574.69	1610.51	1560.77	743.289	3068.82
Z	4	8	4	4	4	2	8
R_1	4.19	3.53	3.14	3.87	3.75	3.98	4.30

Table S17 (cont.). Crystallographic data, experimental and refinement details for studied compounds.

Comp.	**MTS-9**	**MTS-10**	**MTS-11**	**MTS-12**	**MTS-13**	**MTS-14**
ID CCDC	2179050	2179054	CUXBEE [2]	CUXBII [2]	2179056	2179055
Chemical formula	$C_{19}H_{22}O_5$	$C_{19}H_{22}O_5$	$C_{19}H_{22}O_5$	$C_{19}H_{22}O_5$	$C_{19}H_{22}O_5$	$C_{20}H_{24}O_6$
Formula weight [g/mol]	330.375	330.375	330.375	330.375	330.375	360.401
Crystal system	monoclinic	orthorhombic	monoclinic	monoclinic	monoclinic	tetragonal
Space group	P 2_1/c	P ca2_1	P 2_1/c	P 2_1/c	P 2_1/c	P 4_2/n
a, b,c [Å]	10.5321(6)	14.3780(5)	26.3492	7.7187(4)	11.2909(4)	19.7692(5)
	19.5400(13)	8.0758(4)	8.1624(3)	16.2257(6)	10.8019(3)	19.7692(5)
	8.3901(6)	15.0617(7)	8.1890(2)	13.7254(6)	14.4398(4)	4.5558(2)
α. β, γ [°]	90.00	90.00	90.00	90.00	90.00	90.00
	103.754(7)	90.00	97.079(2)	93.205(4)	108.017(3)	90.00
	90.00	90.00	90.00	90.00	90.00	90.00
V [Å^3]	1677.15	1748.87	1747.8	1716.3	1674.77	1780.5
Z	4	4	4	4	4	4
R_1	8.01	5.41	4.20	5.57	3.93	3.41

Table S18. Dihedral angle [°] between two planes for studied compounds.

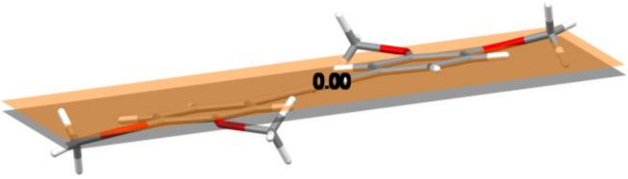

Compound	Dihedral angle [°]
MTS-14	0.00
MTS-7	0.00
MTS-5	2.47
MTS-12	4.94
MTS-4	5.42
MTS-2	6.92
MTS-1	6.99
MTS-9	7.06
MTS-13	10.69
MTS-6	12.21
MTS-10	20.85
MTS-8	24.02
MTS-11	53.53

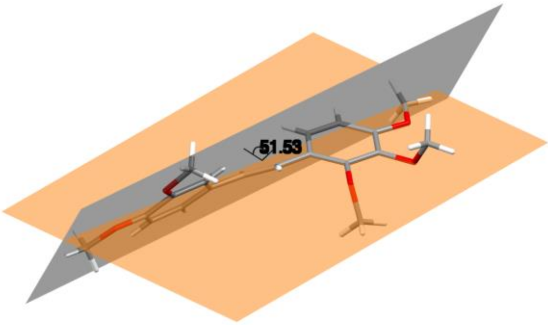

Table S19. Selected torsion angles [°] for compounds with out-of-plane substituent arrangement.

Reason of out-of-plane arrangement of methoxy substituent	Compound	Torsion angle [°]	
**Presence of three substituents at adjacent carbons**	MTS-6	C7-O1-C3-C4	-168.51
		C8-O2-C4-C5	**88.01**
		C9-O3-C5-C4	-176.69
	MTS-10	C131-O13-C13-C12	-163.69
		C121-O12-C12-C11	**64.96**
		C111-O11-C11-C12	-178.90
	MTS-13	C31-O3-C3-O4	175.02
		C41-O4-C4-C5	**-88.84**
		C51-O5-C5-C4	-177.22
	MTS-14	C31-O3-C3-O4	168.30
		C41-O4-C4-C5	**-79.22**
		C51-O5-C5-C4	177.11
	MTS-11	C12-O11-C2-C3	**-75.29**
		C10-O9-C3-C4	**109.64**
**Presence of substituents in both *orto* and *meta* position**		C8-O7-C4-C3	176.30
	MTS-8	C101-O10-C10-C11	**-69.86**
		C111-O11-C11-C10	172.03

3. Chemicals, instruments, and synthesis procedure

All reagents and solvents were purchased from commercial sources (Sigma-Aldrich). Commercial grade reagents were used without further purification. All reactions were performed in oven dried glassware (140°C, 1h) under nitrogen. Described reaction temperatures were measured for external bath. Melting points were determined in an open glass capillary with a Stuart scientific SMP3 apparatus and are uncorrected Reactions were monitored using thin layer chromatography (TLC) plates coated with silica gel (Merck, 60 F254 plates) and visualized with UV (λ_{max} 254 or 365 nm). ESI mass spectrometry analysis was performed on an Agilent 1200 (Agilent, Santa Clara, CA, USA) with an ESI-MS/MS 6410 B Triple Quad detector or NMR experiments were recorded using a Bruker 200, 300, 400 and 500 spectrometers. Chemical shifts (δ) are quoted in parts per million (ppm) and are referred to a residual solvent peak. Coupling constants (*J*) are quoted in Hertz (Hz) to the nearest 0.5 Hz. The abbreviation s, d, t, m, o, and h mean singlet, doublet, triplet, multiplet, overlay and hidden.

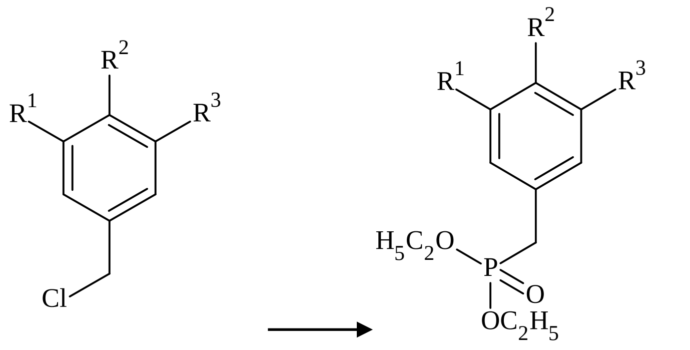

1a: R^1=R^2=OCH_3, R^3=H
1b: R^2=OCH_3, R^1=R^3=H
1c: R^1=R^2= R^3=OCH_3

2a: R^1=R^2=OCH_3, R^3=H
2b: R^2=OCH_3, R^1=R^3=H
2c: R^1=R^2= R^3=OCH_3

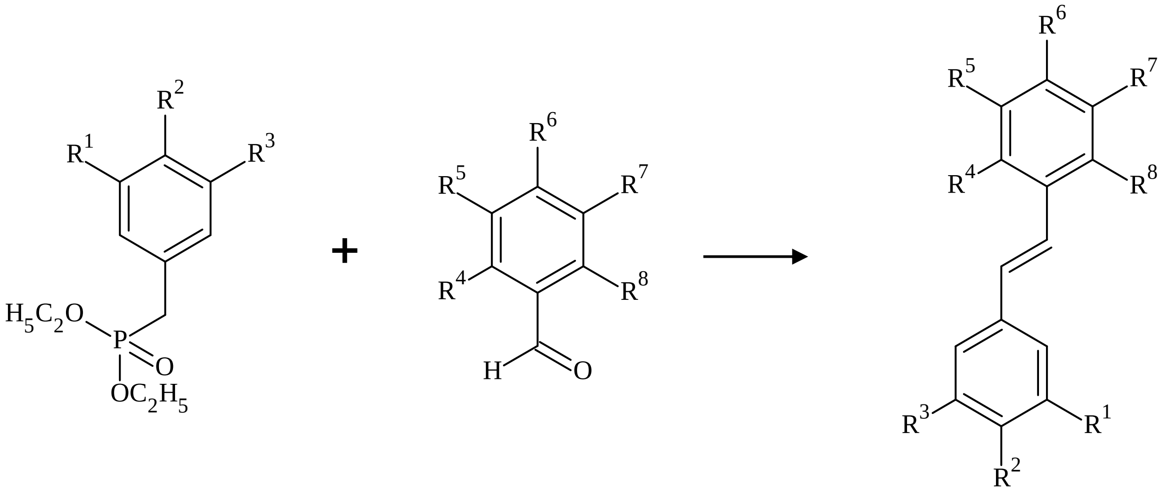

2a: R^1=R^2=OCH_3, R^3=H
2b: R^2=OCH_3, R^1=R^3=H
2c: R^1=R^2= R^3=OCH_3

R^4-R^8 =OCH_3 or H

MTS-1–MTS-14:
R^1-R^8 =OCH_3 or H

Simplified synthetic route for the synthesis of compounds **MTS-1 – MTS-14**.

Synthesis of diethyl (3,4-dimethoxybenzyl)phosphonate (**2a**)

In a round-flask with a $CaCl_2$ tube 3,4-dimethoxybenzyl chloride (**1a**) (3.68 g, 20.0 mmol) and triethyl phosphite (3.81 mL, 21.9 mmol) were placed and heated at 140°C for 24 h. Resulting product (**2a**) diethyl (3,4-dimethoxybenzyl)phosphonate was isolated from reaction mixture by distillation at reduced pressure to yield pale, viscous oil of compound **2a** (3.15 g, 55%). The procedure was repeated four times. ^{1}H NMR (400 MHz, DMSO-d_6) δ [ppm]: 6.88-6.87 (m, 2H), 6.80-6.78 (m, 1H), 3.98-3.90 (m, 4H), 3.72 (s, 6H), 3.13 ($J_{H,P}$ = 22.0 Hz), 1.17 (t, J = 7.0 Hz). ^{13}C NMR (100 MHz, DMSO-d_6) δ 148.8 ($J_{C,P}$ = 3.0 Hz), 148.0 ($J_{C,P}$ = 4.0 Hz), 124.8 ($J_{C,P}$ = 9.0 Hz), 122.3 ($J_{C,P}$ = 7.0 Hz), 114.0 ($J_{C,P}$ = 6.0 Hz), 112.2 ($J_{C,P}$ = 3.0 Hz), 66.7 ($J_{C,P}$ = 8.0 Hz), 55.9, 55.9, 32.1 ($J_{C,P}$ = 135.0 Hz), 16.7 ($J_{C,P}$ = 5.0 Hz). MS(ESI) m/z 288 [M]+.

Synthesis of diethyl 4-methoxybenzylphosphonate (**2b**)

In a round-flask with a $CaCl_2$ tube 4-methoxybenzyl chloride (**1b**) (1.56 g, 10.0 mmol) and triethyl phosphite (1.90 mL, 11.0 mmol) were placed and heated at 140°C for 24 h. Resulting product diethyl (4-methoxyobenzyl)phosphonate (**2b**) was isolated from reaction mixture by distillation at reduced pressure to yield colorless, viscous oil of compound **2b** (2.06 g, 80%). ^{1}H NMR ($CDCl_3$): δ 7.23–7.18 (m, 2H), 6.84 (d, J = 8.6 Hz, 2H), 4.15–3.93 (m, 4H), 3.78 (s, 3H), 3.09 (d, $J_{H,P}$ = 21.0 Hz, 2H), 1.24 (t, J = 7.1 Hz, 3H); ^{13}C NMR ($CDCl_3$): δ 158.5 ($J_{C,P}$ = 3.4 Hz), 130.6 ($J_{C,P}$ = 6.5 Hz), 123.2 ($J_{C,P}$ = 9.6 Hz), 113.9 ($J_{C,P}$ = 2.7 Hz), 62.1 ($J_{C,P}$ = 6.9 Hz), 55.1, 32.6 ($J_{C,P}$ = 139.1 Hz), 16.2 ($J_{C,P}$ = 6.1 Hz). MS(ESI) m/z 258 [M]+.

Synthesis of diethyl 3,4,5-trimethoxybenzylphosphonate (**2c**)

In a round-flask with a $CaCl_2$ tube 3,4,5-trimethoxybenzyl chloride (**1c**) (2.17 g, 10.0 mmol) and triethyl phosphite (1.90 mL, 11.0 mmol) were placed and heated at 140°C for 24 h. Resulting product diethyl (3,4,5-trimethoxyobenzyl)phosphonate (**2c**) was isolated from reaction mixture by distillation at reduced pressure to yield yellow, viscous oil of compound **2c** (2.80 g, 88%). ^{1}H NMR ($CDCl_3$): δ 6.54 (d, $J_{H,P}$ = 2.5 Hz, 2H), 4.11–3.97 (m, 4H), 3.85 (s, 6H), 3.82 (s, 3H), 3.09 (d, $J_{H,P}$ = 21.5 Hz, 2H), 1.27 (t, J = 7.1 Hz, 6H); ^{13}C NMR ($CDCl_3$): 153.0 ($J_{C,P}$ = 3.0 Hz), 126.9 ($J_{C,P}$ = 9.2 Hz), 106.7 ($J C,P$ = 6.5 Hz), 62.1 ($J_{C,P}$ = 6.5 Hz), 60.8, 55.9, 33.8 ($J_{C,P}$ = 138.7 Hz), 16.3 ($J_{C,P}$ = 6.1 Hz); MS(ESI) m/z 318 [M]+.

Synthesis of 2,4′-dimethoxy-*trans*-stilbene (**MTS-1**)

Diethyl 4-methoxybenzylphosphonate **2b** (0.50 g, 1.94 mmol) and 10 mL of dry DMF were placed in round-flask and cooled to 0°C in ambient atmosphere (under nitrogen). Next sodium methoxide (0.21 g, 5.81 mmol) and 2-methoxybenzaldehyde (0.26 g, 1.94 mmol) was added and resulting reaction mixture was strongly stirred under nitrogen for 1 h in room temperature and next 1.5 h in 100°C. After cooling to room temperature reaction mixture was poured into 100 mL of water with crushed ice and left overnight. Resulting precipitate was filtered off,

washed with distilled water (3×15 mL) and purified by crystallization from 95% ethanol. It gave 2,4′-dimethoxy-*trans*-stilbene (**MTS-1**) as white crystalline solid (0.35 g, 75%). M.p. = 91–93°C. ^{1}H NMR (500 MHz, DMSO-d_6) δ 7.62 (dd, *J* = 7.5, 1.0 Hz, 1H), 7.49 (d, *J* = 8.5 Hz, 2H), 7.27 (d, *J* = 16.5 Hz, 1H), 7.25–7.20 (m, 1H), 7.15 (d, *J* = 16.5 Hz, 1H), 7.02 (d, *J* = 8.0 Hz, 1H), 6.96 (to, *J* = 7.5 Hz, 1H), 6.94 (do, *J* = 8.5 Hz, 2H), 3.84 (s, 3H), 3.77 (s, 3H). ^{13}C NMR (125 MHz, DMSO-d_6) δ 158.9, 156.3, 130.1, 128.4, 128.4, 127.6, 125.9, 125.8, 120.6, 120.6, 114.2, 111.3, 55.4, 55.1. MS(ESI) m/z 240 [M]+.

Synthesis of 3,4-dimethoxy-*trans*-stilbene (**MTS-2**)

Diethyl (3,4-dimethoxybenzyl)phosphonate **2a** (1.01 g, 3.5 mmol) and 15 mL of dry DMF were placed in round-flask and cooled to 0°C in ambient atmosphere (under nitrogen). Next sodium methoxide (0.38 g, 7.0 mmol) and benzaldehyde (0.37 g, 3.5 mmol) was added and resulting reaction mixture was strongly stirred under nitrogen for 1h in room temperature and 90 minutes in temperature 100°C. Reaction was quenched by pouring into 100 ml water with ice and left overnight. Resulting precipitate was filtered off, washed with distilled water (2×20 mL) and recrystalized with ethanol which gave white solid of 3,4-dimethoxy-*trans*-stilbene (**MTS-2**) (0.52 g, 62%). M.p. = 108-109°C. ^{1}H NMR (400 MHz, DMSO-d_6) δ 7.57 (d, *J* = 7.5 Hz, 2H), 7.36 (t, *J* = 7.6 Hz, 2H), 7.25 (so, 1H), 7.24 (to, *J* = 7.5 Hz, 1H), 7.19 (d, *J* = 16.5 Hz, 1H), 7.13 (do, *J* = 16.5 Hz, 1H), 7.10 (ddo, *J* = 8.3 Hz, *J* = 1.7 Hz, 1H), 6.95 (d, *J* = 8.3 Hz, 1H), 3.83 (s, 3H), 3.77 (s, 3H). ^{13}C NMR (100 MHz, DMSO-d_6) δ 148.9, 148.8, 137.4, 130.0, 128.6, 128.4, 127.1, 126.2, 126.1, 119.9, 111.8, 109.2, 55.5, 55.5. MS(ESI) m/z 240 [M]+.

Synthesis of 3,4,2',5'-tetramethoxy-*trans*-stilbene (**MTS-3**)

Diethyl (3,4-dimethoxybenzyl)phosphonate **2a** (1.44 g, 5.0 mmol) and 15 mL of dry DMF were placed in round-flask and cooled to 0°C in ambient atmosphere (under nitrogen). Next sodium methoxide (0.54 g, 10.0 mmol) and 2,5-dimethoxybenzaldehyde (0.83g, 5.0 mmol) was added and resulting reaction mixture was strongly stirred under nitrogen for 1h in room temperature and 90 minutes in temperature 100°C. Reaction was quenched by pouring into 100 ml water with ice and left overnight. Resulting precipitate was filtered off, washed with distilled water (2×20 mL) and recrystalized with ethanol which gave yellow solid of 3,4,2',5'-tetramethoxy-*trans*-stilbene (**MTS-3**) (0.46 g, 33%). M.p. = 86-88°C. ^{1}H NMR (400 MHz, DMSO-d_6) δ 7.26 (d, *J* = 16.5 Hz, 1H), 7.21 (so, 1H), 7.19 (do, *J* = 16.9 Hz, 3H), 7.17 (so, 1H), 7.10 (d, *J* = 8.3 Hz, 1H), 6.95 (do, *J* = 9.1 Hz, 1H), 6.95h (do, *J* = 9.1 Hz, 1H) 6.82 (dd, *J* = 8.9, 2.7 Hz, 1H), 3.82 (s, 3H), 3.80 (s, 3H), 3.78 (s, 3H), 3.76 (s, 3H). ^{13}C NMR (100 MHz, DMSO-d_6) δ 153.8, 151.2, 149.4, 149.2, 130.8, 129.7, 127.0, 121.1, 120.0, 114.1, 113.0, 112.3, 111.6, 109.9, 56.5, 56.0, 55.9, 55.8. MS(ESI) m/z 300 [M]+.

Synthesis of 3,4,2',6'-tetramethoxy-*trans*-stilbene (**MTS-4**)

Diethyl (3,4-dimethoxybenzyl)phosphonate **2a** (1.74 g, 6.0 mmol) and 15 mL of dry DMF were placed in round-flask and cooled to 0°C in ambient atmosphere (under nitrogen). Next sodium methoxide (0.65 g, 12.0 mmol) and 2,6-dimethoxybenzaldehyde (1.00 g 6.0 mmol) was added and resulting reaction mixture was strongly stirred under nitrogen for 1h in room temperature and 90 minutes in temperature 100°C. Reaction was quenched by pouring into 100 ml water with ice and left overnight. Resulting precipitate was filtered off, washed with distilled water (2×20 mL) and recrystalized with ethanol which gave white solid of 3,4,2',6'-tetramethoxy-*trans*-stilbene (**MTS-4**) (1.24 g, 69%). M.p. = 111-112°C. ^{1}H NMR (400 MHz, DMSO-d_6) δ 7.43 (d, *J* = 16.7 Hz, 1H), 7.22 (do, *J* = 16.6 Hz, 1H), 7.18 (to, *J* = 8.3 Hz, 1H), 7.07 (d, *J* = 1.2 Hz, 1H), 7.03 (d, *J* = 8.3 Hz, 1H), 6.93 (d, *J* = 8.3 Hz, 1H), 6.69 (d, *J* = 8.4 Hz, 2H), 3.84 (s, 6H), 3.81 (s, 3H), 3.76 (s, 3H). ^{13}C NMR (100 MHz, DMSO-d_6) δ 158.0, 148.9, 148.4, 131.7, 131.6, 128.1, 118.7, 117.6, 113.8, 111.9, 109.4, 104.2, 55.7, 55.7h, 55.5, 55.4. MS(ESI) m/z 300 [M]+.

Synthesis of 3,4,3',5'-tetramethoxy-*trans*-stilbene (**MTS-5**)

Diethyl (3,4-dimethoxybenzyl)phosphonate **2a** (1.16 g, 4.0 mmol) and 15 mL of dry DMF were placed in round-flask and cooled to 0°C in ambient atmosphere (under nitrogen). Next sodium methoxide (0.45 g, 8.0 mmol) and 3,5-dimethoxybenzaldehyde (0.67 g 4.0 mmol) was added and resulting reaction mixture was strongly stirred under nitrogen for 1h in room temperature and 90 minutes in temperature 100°C. Reaction was quenched by pouring into 100 ml water with ice and left overnight. Resulting precipitate was filtered off, washed with distilled water (2×20 mL) and recrystalized with ethanol which gave white solid of 3,4,2',6'-tetramethoxy-*trans*-stilbene (**MTS-5**) (0.71 g, 59%). M.p. = 67-69°C. ^{1}H NMR ($CDCl_3$): δ 7.08–7.00 (m, 3H), 6.93–6.83 (m, 2H), 6.66 (d, *J* = 2.2Hz, 2H), 6.39–6.37 (m, 1H), 3.94 (s, 3H), 3.90 (s, 3H), 3.82 (s, 6H). ^{13}C NMR ($CDCl_3$): δ 160.9, 149.0, 148.9, 139.5, 130.1, 128.9, 126.7, 119.9, 111.1, 108.7, 104.2, 99.6, 55.9, 55.8, 55.3. MS(ESI) m/z 300 [M]+.

Synthesis of 3,4,5,4'-tetramethoxy-*trans*-stilbene (**MTS-6**)

Diethyl (4-methoxybenzyl)phosphonate **2b** (0.77 g, 3.0 mmol) and 15 mL of dry DMF were placed in round-flask and cooled to 0°C in ambient atmosphere (under nitrogen). Next sodium methoxide (0.34 g, 6.0 mmol) and 3,4,5-trimethoxybenzaldehyde (0.59 g 3.0 mmol) was added and resulting reaction mixture was strongly stirred under nitrogen for 1h in room temperature and 90 minutes in temperature 100°C. Reaction was quenched by pouring into 100 ml water with ice and left overnight. Resulting precipitate was filtered off, washed with distilled water (2×20 mL) and recrystalized with ethanol which gave white solid of 3,4,5,4'-tetramethoxy-*trans*-stilbene (**MTS-6**) (0.48 g, 53%). M.p. = 156-158°C. ^{1}H NMR ($CDCl_3$): δ 7.44 (d, *J* = 8.8Hz, 2H), 6.98 (d, *J* = 16.3Hz, 1H), 6.91–6.83 (m, 3H), 6.71 (s, 2H), 3.90 (s, 6H), 3.86 (s, 3H), 3.82 (s, 3H). ^{13}C NMR ($CDCl_3$): δ 159.2, 153.3, 137.7, 133.3, 129.9, 127.6, 127.5, 126.4, 114.1, 103.2, 60.9,56.0, 55.2. MS(ESI) m/z 300 [M]+.

Synthesis of 3,4,3′,4′-tetramethoxy-*trans*-stilbene (**MTS-7**)

Diethyl (3,4-dimethoxybenzyl)phosphonate **2a** (1.16 g, 4.0 mmol) and 15 mL of dry DMF were placed in round-flask and cooled to 0°C in ambient atmosphere (under nitrogen). Next sodium methoxide (0.45 g, 8.0 mmol) and 3,4-dimethoxybenzaldehyde (0.67 g 4.0 mmol) was added and resulting reaction mixture was strongly stirred under nitrogen for 1h in room temperature and 90 minutes in temperature 100°C. Reaction was quenched by pouring into 100 ml water with ice and left overnight. Resulting precipitate was filtered off, washed with distilled water (2×20 mL) and recrystalized with ethanol which gave white solid of 3,4,3′,4′-tetramethoxy-*trans*-stilbene (**MTS-7**) (0.77 g, 64%). M.p. = 151-152°C. ^{1}H NMR (400 MHz, DMSO-d_6): δ 7.22 (d, J = 1.7 Hz, 2H), 7.15 – 7.01 (m, 4H), 6.94 (d, J = 8.3 Hz, 2H), 3.83 (s, 6H), 3.77 (s, 6H). ^{13}C NMR (101 MHz, DMSO-d_6) δ 149.41, 148.86, 130.83, 126.77, 119.88, 112.28, 109.46, 55.94, 55.90. MS(ESI) m/z 300 [M]+.

Synthesis of 3,4,2',3'-tetramethoxy-*trans*-stilbene (**MTS-8**)

Diethyl (3,4-dimethoxybenzyl)phosphonate **2a** (0.87 g, 3.0 mmol) and 15 mL of dry DMF were placed in round-flask and cooled to 0°C in ambient atmosphere (under nitrogen). Next sodium methoxide (0.34 g, 6.0 mmol) and 2,3-dimethoxybenzaldehyde (0.50 g 3.0 mmol) was added and resulting reaction mixture was strongly stirred under nitrogen for 1h in room temperature and 90 minutes in temperature 100°C. Reaction was quenched by pouring into 100 ml water with ice and left overnight. Resulting precipitate was filtered off, washed with distilled water (2×20 mL) and recrystalized with methanol which gave white solid of 3,4,2',3'-tetramethoxy-*trans*-stilbene (**MTS-8**) (0.47 g, 52%). M.p. = 111-112°C. ^{1}H NMR (500 MHz, DMSO-d_6) δ 7.27 (ddo, *J* =7.7, 0.9 Hz, 1H), 7.24 (d, *J* = 16.2 Hz, 1H), 7.19 (sh, 1H), 7.17 (d, *J* = 16.6 Hz, 1H), 7.12 (dd, *J* = 8.3, 1.8 Hz, 1H), 7.06 (t, *J* = 8.0 Hz, 1H), 6.96 (do, *J* = 8.4 Hz, 1H), 6.94 (ddo, *J* = 8.3, 1.0 Hz, 1H), 3.82 (s, 3H), 3.81 (s, 3H), 3.77 (s, 3H), 3.75 (s, 3H). ^{13}C NMR (125 MHz, DMSO-d_6) δ 153.3, 149.4, 149.3, 146.6, 131.4, 130.6, 130.2, 124.6, 120.7, 120.1, 117.9, 112.3, 112.0, 110.1, 60.9, 56.1, 56.0, 56.0. MS(ESI) m/z 300 [M]+.

Synthesis of 3,4,2',4',5'-pentamethoxy-*trans*-stilbene (**MTS-9**)

Diethyl (3,4-dimethoxybenzyl)phosphonate **2a** (0.72 g, 2.5 mmol) and 15 mL of dry DMF were placed in round-flask and cooled to 0°C in ambient atmosphere (under nitrogen). Next sodium methoxide (0.27 g, 5.0 mmol) and 2,4,5-trimethoxybenzaldehyde (0.49 g 2.5 mmol) was added and resulting reaction mixture was strongly stirred under nitrogen for 1h in room temperature and 90 minutes in temperature 100°C. Reaction was quenched by pouring into 100 ml water with ice and left overnight. Resulting precipitate was filtered off, washed with distilled water (2×20 mL) and recrystalized with ethanol which gave yellow solid of 3,4,2',4',5'-pentamethoxy-*trans*-stilbene (**MTS-9**) (0.43 g, 53%). M.p. = 122-124°C. ^{1}H NMR (400 MHz, DMSO-d_6) δ 7.23 (do, *J* = 16.4 Hz, 1H), 7.23 (so, 1H), 7.13 (s, 1H), 7.08 (do, *J* = 17.7 Hz, 1H), 7.07 (do, *J* = 6.5 Hz, 1H), 6.94 (d, *J* = 8.3 Hz, 1H), 6.71 (s, 1H), 3.85 (s, 3H), 3.81 (s, 3H), 3.81 (sh, 3H), 3.79 (s, 3H), 3.77

(s, 3H). ^{13}C NMR (100 MHz, DMSO-d_6) δ 151.6, 149.8, 149.4, 148.74, 143.5, 131.3, 127.0, 121.0, 119.4, 117.8, 112.37, 110.4, 109.7, 98.7, 56.8, 56. 7, 56.2, 55.9, 55.9. MS(ESI) m/z 330 [M]+.

Synthesis of 3,3′,4,4',5-pentamethoxy-*trans*-stilbene (**MTS-10**)

Diethyl (3,4-dimethoxybenzyl)phosphonate (**2a**) (1.16 g, 4.0 mmol), sodium methoxide (0.45 g, 8.0 mmol) and 15 mL of dry DMF were placed in round-flask and cooled to 0°C in ambient atmosphere (under nitrogen), then 3,4,5-trimethoxybenzaldehyde (0.78 g 4.0 mmol) was added. Within an hour, the reaction mixture was stirred and allowed to reach room temperature. Next reaction mixture was heated by 90 minutes in temperature 100°C. Reaction was quenched by pouring into 100 ml water with ice. Resulting white precipitate was filtered off and recrystalized with ethanol which gave white solid of 3,3′,4,4',5-pentamethoxy-*trans*-stilbene (**MTS-10**) (0.58 g, 44%). M.p. = 140-141°C. ^{1}H NMR (400 MHz, DMSO- d_6) δ 7.26 (d, *J* = 22.2 Hz, 1H), 7.16 (d, *J* = 16.3 Hz, 1H), 7.11 – 7.04 (m, 2H), 6.95 (d, *J* = 8.2 Hz, 1H), 6.90 (s, 2H), 3.83 (s, 9H), 3.77 (s, 3H), 3.68 (s, 3H). ^{13}C NMR (101 MHz, DMSO-d_6) δ 153.04, 148.95, 148.61, 136.94, 133.13, 130.07, 127.77, 126.40, 119.68, 111.79, 109.00, 103.48, 60.04, 55.80, 55.46, 55.41. 56.3, 55.9. MS(ESI) m/z 330 [M]+.

Synthesis of 3,4,2',3',4'-pentamethoxy-*trans*-stilbene (**MTS-11**)

Diethyl (3,4-dimethoxybenzyl)phosphonate **2a** (0.72 g, 2.5 mmol) and 15 mL of dry DMF were placed in round-flask and cooled to 0°C in ambient atmosphere (under nitrogen). Next sodium methoxide (0.27 g, 5.0 mmol) and 2,3,4-trimethoxybenzaldehyde (0.49 g 2.5 mmol) was added and resulting reaction mixture was strongly stirred under nitrogen for 1h in room temperature and 90 minutes in temperature 100°C. Reaction was quenched by pouring into 100 ml water with ice and left overnight. Resulting precipitate was filtered off, washed with distilled water (2×20 mL) and recrystalized with ethanol which gave white solid of 3,4,2',3',4'-pentamethoxy-*trans*-stilbene (**MTS-11**) (0.33 g, 40%). M.p. = 105-106°C. ^{1}H NMR (400 MHz, DMSO-d_6) δ 7.36 (d, *J* = 8.8 Hz, 1H), 7.17 (so, 1H), 7.16 (do, *J* = 16.4 Hz, 2H), 7.08 (do, *J* = 5.9 Hz, 2H), 7.07 (do, *J* = 16.7 Hz, 1H), 6.94 (d, *J* = 8.3 Hz, 1H), 6.83 (d, *J* = 8.8 Hz, 1H), 3.83 (so, 3H), 3.82 (so, 6H), 3.77 (s, 6H). ^{13}C NMR (100 MHz, DMSO-d_6) δ 153.2, 151.5, 149.4, 149.0, 142.4, 131.0, 128.2, 124.4, 121.0, 121.0, 119.7, 112.3, 109.9, 108.9, 61.5, 60.8. MS(ESI) m/z 330 [M]+.

Synthesis of 3,4,2',4′,6'-pentamethoxy-*trans*-stilbene (**MTS-12**)

Diethyl (3,4-dimethoxybenzyl)phosphonate **2a** (1.44 g, 5.0 mmol) and 15 mL of dry DMF were placed in round-flask and cooled to 0°C in ambient atmosphere (under nitrogen). Next sodium methoxide (0.54 g, 10.0 mmol) and 2,4,6-trimethoxybenzaldehyde (0.98 g, 5.0 mmol) was added and resulting reaction mixture was strongly stirred under nitrogen for 1h in room temperature and 90 minutes in temperature 100°C. Reaction was quenched by pouring into 100 ml water with ice and left overnight. Resulting precipitate was filtered off, washed with distilled water (2×20 mL) and recrystalized with methanol which gave white solid of 3,4,2',4′,6'-

pentamethoxy-*trans*-stilbene (**MTS-12**) (1.44 g, 51%). M.p. = 128-131°C. ^{1}H NMR (400 MHz, DMSO-d_6) δ 7.32 (d, *J* = 16.6 Hz, 1H), 7.16 (d, *J* = 16.6 Hz, 1H), 7.03 (d, *J* = 1.5 Hz, 1H), 6.99 (dd, *J* = 8.3, 1.5 Hz, 1H), 6.91 (d, *J* = 8.3 Hz, 1H), 6.29 (s, 2H), 3.85 (s, 6H), 3.81 (s, 3H), 3.80 (s, 3H), 3.75 (s, 3H). ^{13}C NMR (101 MHz, DMSO-d_6) δ 159.9, 158.9, 148.9, 148.0, 132.1, 129.1, 118.3, 117.7, 112.0, 109.2, 107.0, 91.0, 55.7, 55.5, 55.4, 55.2. MS(ESI) m/z 330 [M]+.

Synthesis of 3,5,3',4′,5'-pentamethoxy-*trans*-stilbene (**MTS-13**)

Diethyl (3,4,5-trimethoxybenzyl)phosphonate **2c** (0.95 g, 3.0 mmol) and 15 mL of dry DMF were placed in round-flask and cooled to 0°C in ambient atmosphere (under nitrogen). Next sodium methoxide (0.34 g, 6.0 mmol) and 3,5-dimethoxybenzaldehyde (0.50 g 3.0 mmol) was added and resulting reaction mixture was strongly stirred under nitrogen for 1h in room temperature and 90 minutes in temperature 100°C. Reaction was quenched by pouring into 100 ml water with ice and left overnight. Resulting precipitate was filtered off, washed with distilled water (2×20 mL) and recrystalized with methanol which gave white solid of 3,5,3',4′,5'-pentamethoxy-*trans*-stilbene (**MTS-13**) (0.56 g, 57%). M.p. = 136-137°C. ^{1}H NMR ($CDCl_3$) δ 7.02 (d, *J* = 16.1Hz, 1H), 6.91 (d, *J* = 16.1Hz, 1H), 6.73 (s, 2H), 6.66 (d, *J* = 2.2Hz, 2H), 6.40–6.38 (m, 1H), 3.91 (s, 6H), 3.87 (s, 3H), 3.82 (s, 6H). ^{13}C NMR ($CDCl_3$) δ 160.9, 153.3, 139.1, 137.9, 132.7, 129.0, 128.0, 104.4, 103.5, 99.8, 60.9, 56.0, 55.2. MS(ESI) m/z 330 [M]+. MS(ESI) m/z 330 [M]+.

Synthesis of 3,4,5,3',4′,5'-hexamethoxy-*trans*-stilbene (**MTS-14**)

Diethyl (3,4,5-trimethoxybenzyl)phosphonate **2c** (0.95 g, 3.0 mmol) and 15 mL of dry DMF were placed in round-flask and cooled to 0°C in ambient atmosphere (under nitrogen). Next sodium methoxide (0.34 g, 6.0 mmol) and 3,4,5-trimethoxybenzaldehyde (0.59 g 3.0 mmol) was added and resulting reaction mixture was strongly stirred under nitrogen for 1h in room temperature and 90 minutes in temperature 100°C. Reaction was quenched by pouring into 100 ml water with ice and left overnight. Resulting precipitate was filtered off, washed with distilled water (2×20 mL) and recrystalized with methanol which gave white solid of 3,4,5,3',4′,5'-hexamethoxy-*trans*-stilbene (**MTS-14**) (0.49 g, 45%). M.p. = 213-215°C. ^{1}H NMR ($CDCl_3$) δ 6.94 (s, 2H), 6.74 (s, 4H), 3.92 (s, 12H), 3.87 (s, 6H). ^{13}C NMR ($CDCl_3$) δ 153.3, 137.8, 132.8, 128.0, 103.3, 60.9, 56.0. MS(ESI) m/z 360 [M]+.

4. NMR data

NMR data for **MTS-7**

^{1}H NMR

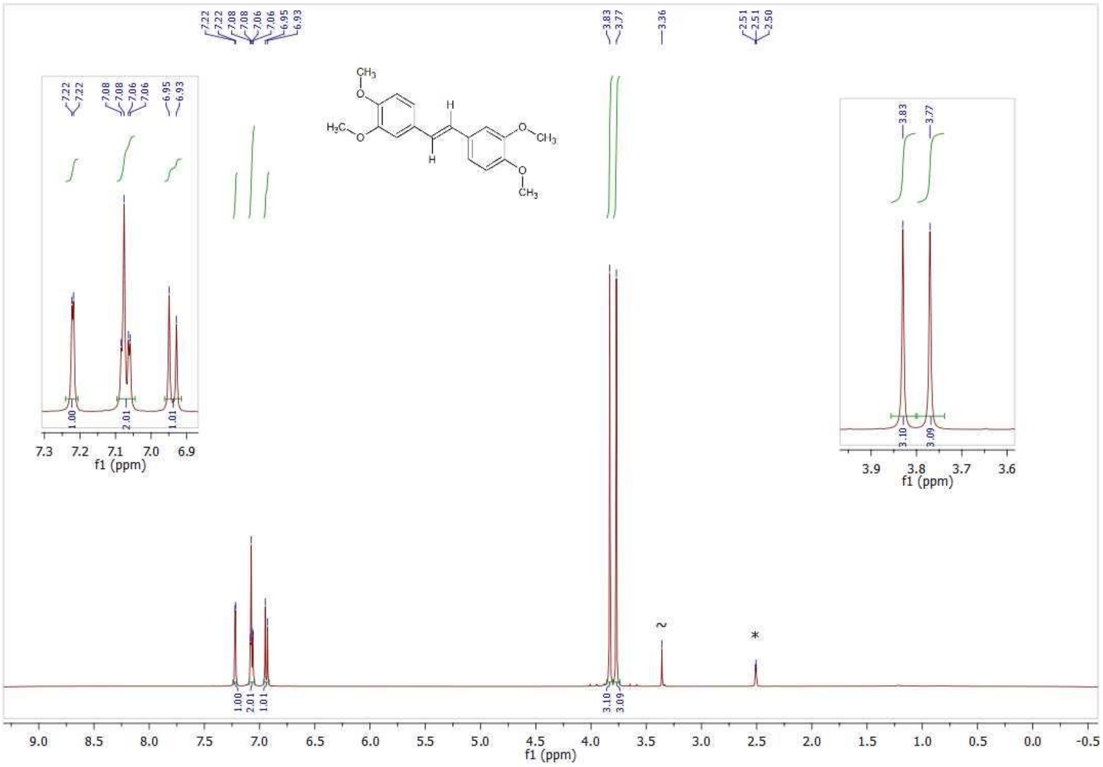

^{13}C NMR

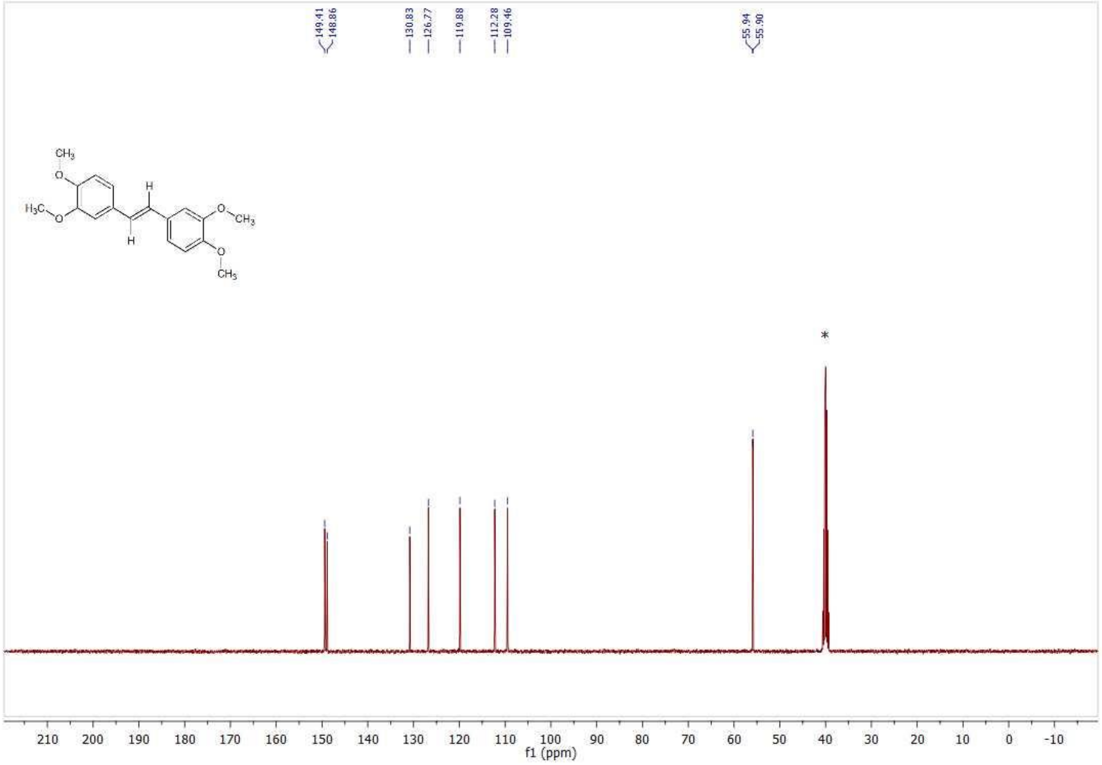

^1H-^1H COSY

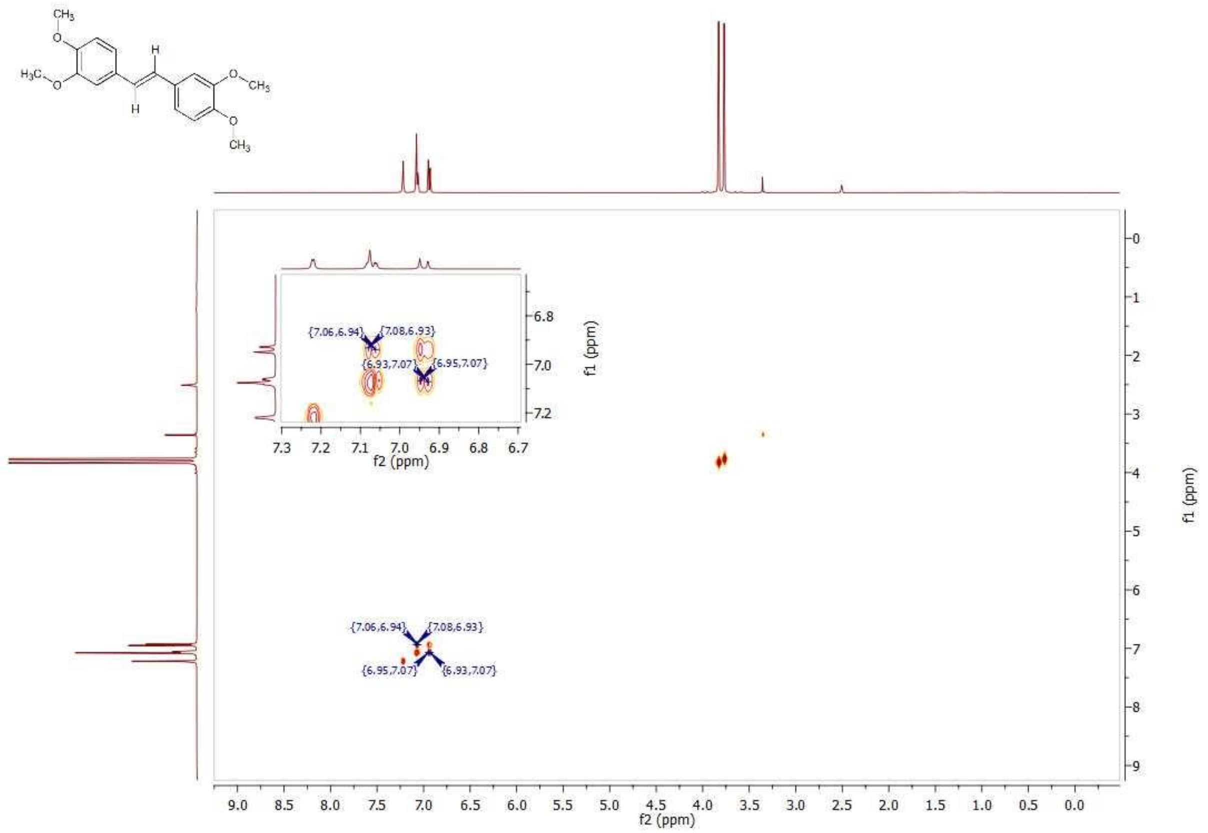

^1H-^{13}C HSQC

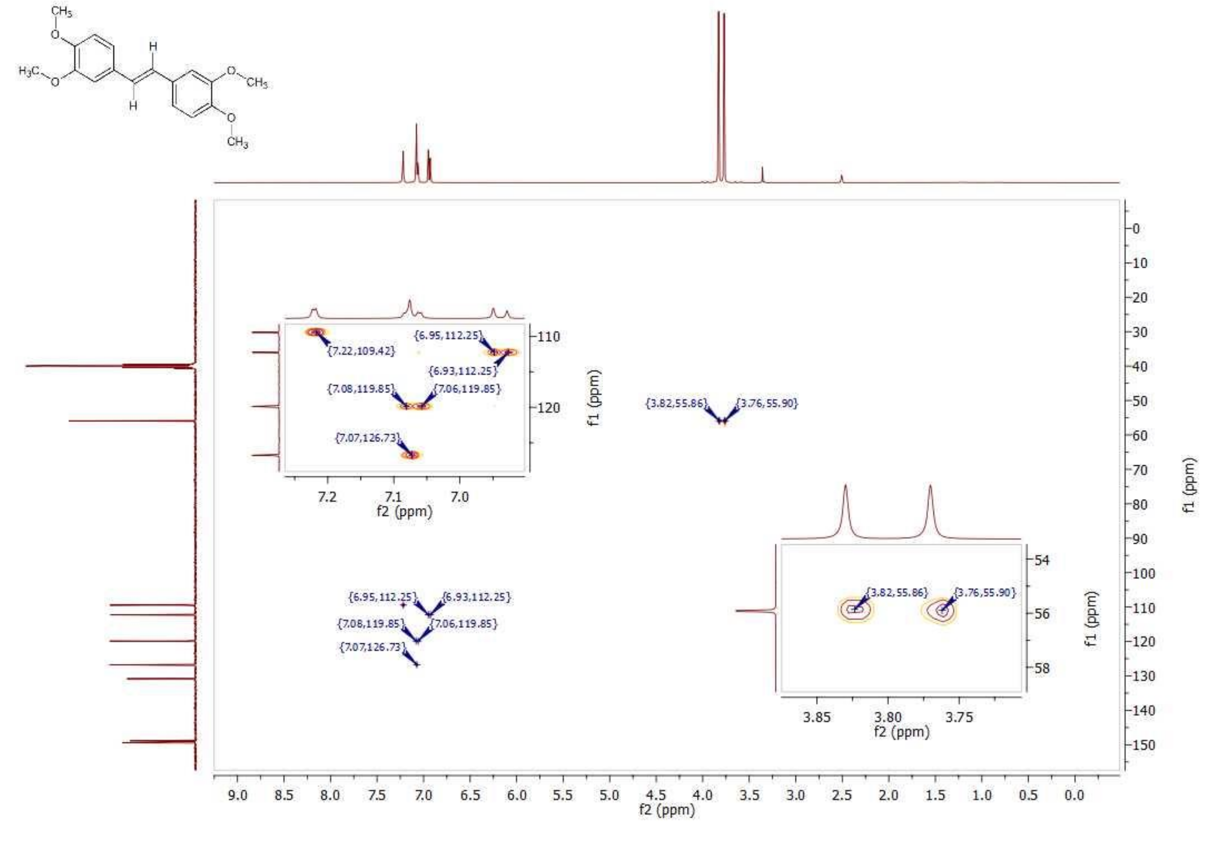

^{1}H-^{13}C HMBC

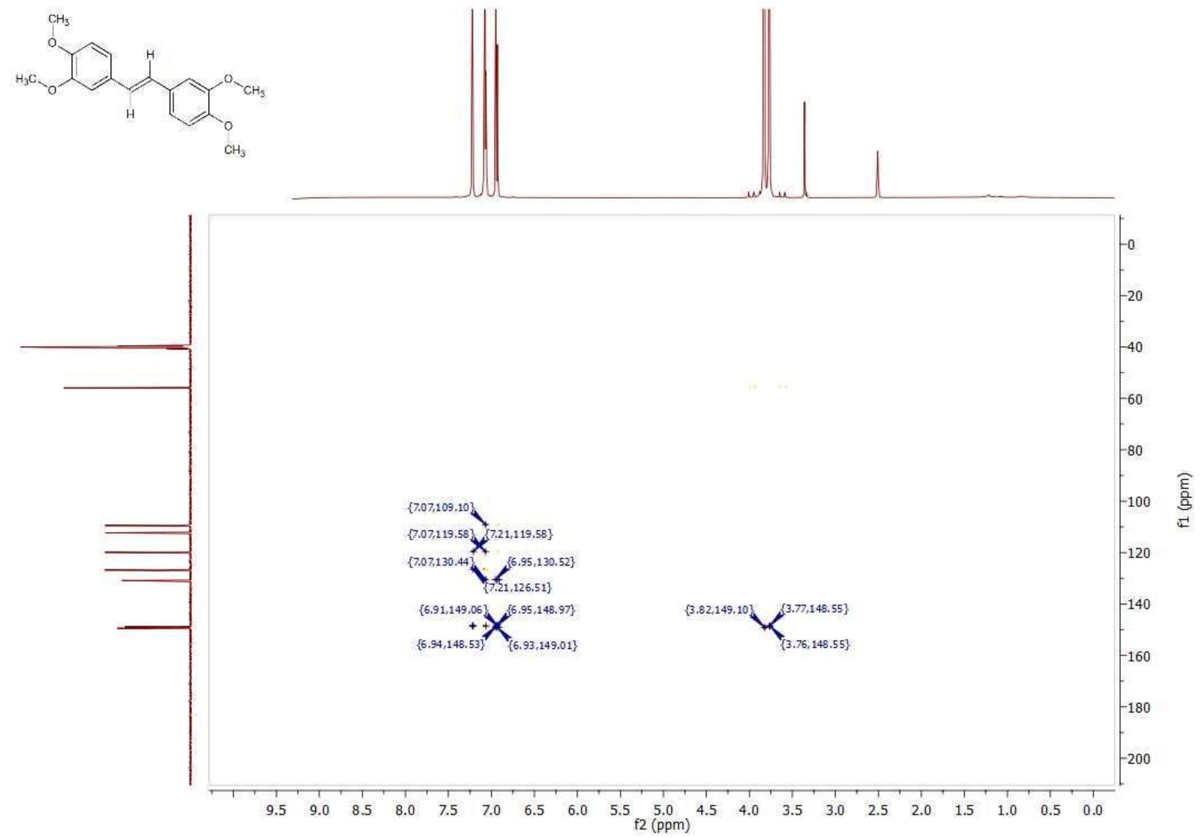
{7.07,109.10}
{7.07,119.58}
{7.21,119.58}
{7.07,130.44}
{6.95,130.52}
{7.21,126.51}
{6.91,149.06}
{6.95,148.97}
{6.94,148.53}
{6.93,149.01}
{3.82,149.10}
{3.77,148.55}
{3.76,148.55}
f2 (ppm)
f1 (ppm)

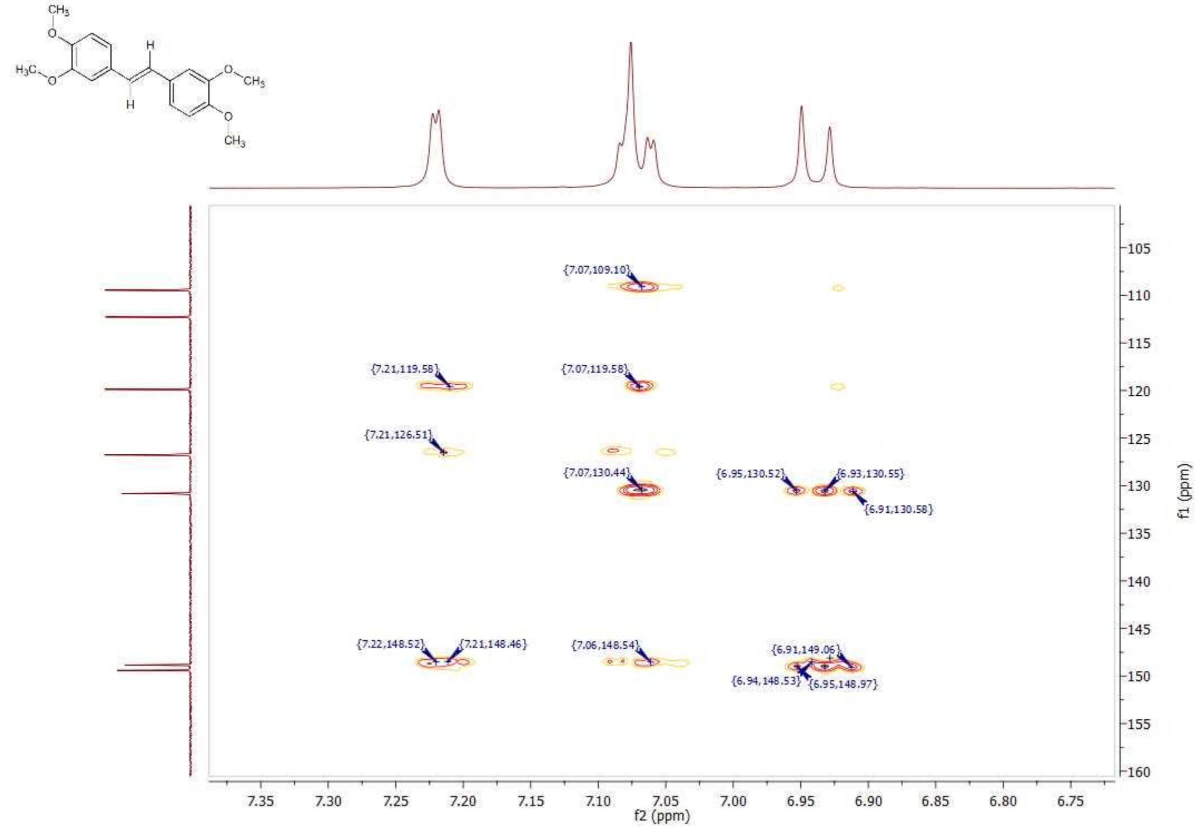
{7.07,109.10}
{7.21,119.58}
{7.07,119.58}
{7.21,126.51}
{7.07,130.44}
{6.95,130.52}
{6.93,130.55}
{6.91,130.58}
{7.22,148.52}
{7.21,148.46}
{7.06,148.54}
{6.91,149.06}
{6.94,148.53}
{6.95,148.97}
f2 (ppm)
f1 (ppm)

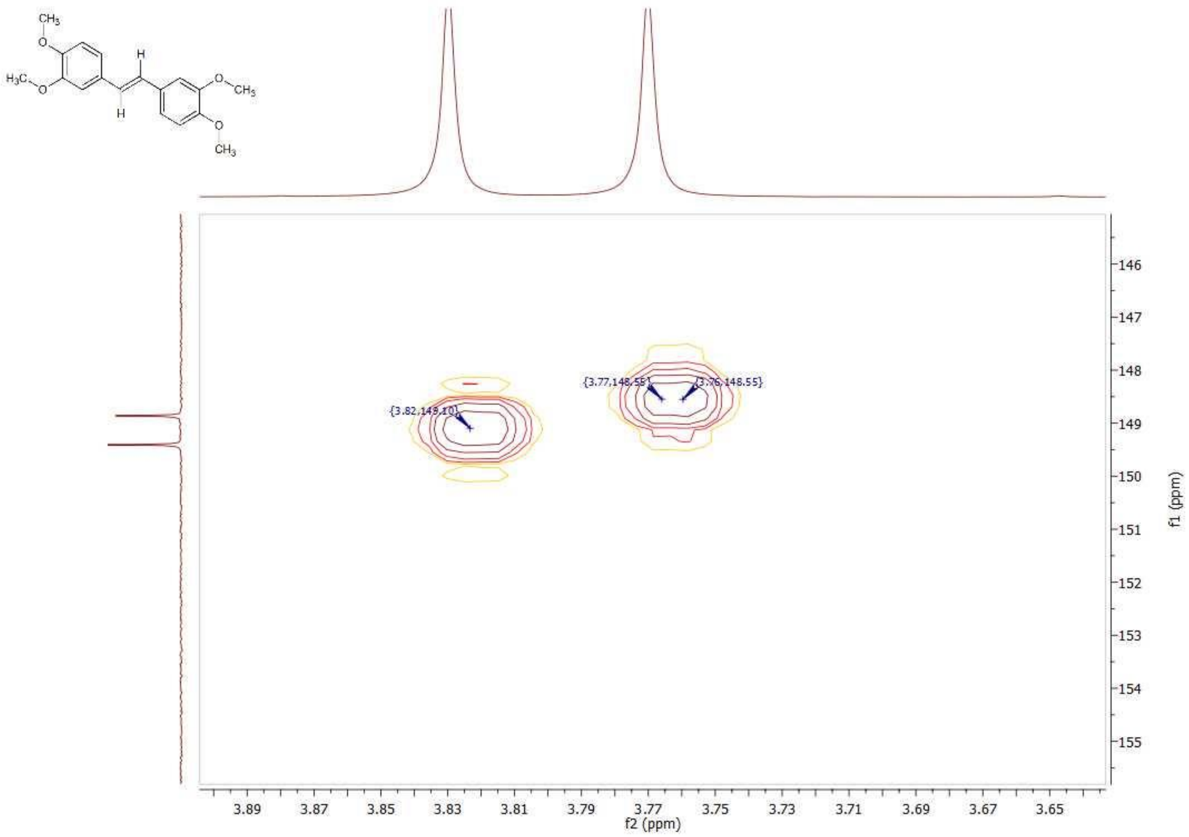

NMR data for **MTS-10**

^{1}H NMR

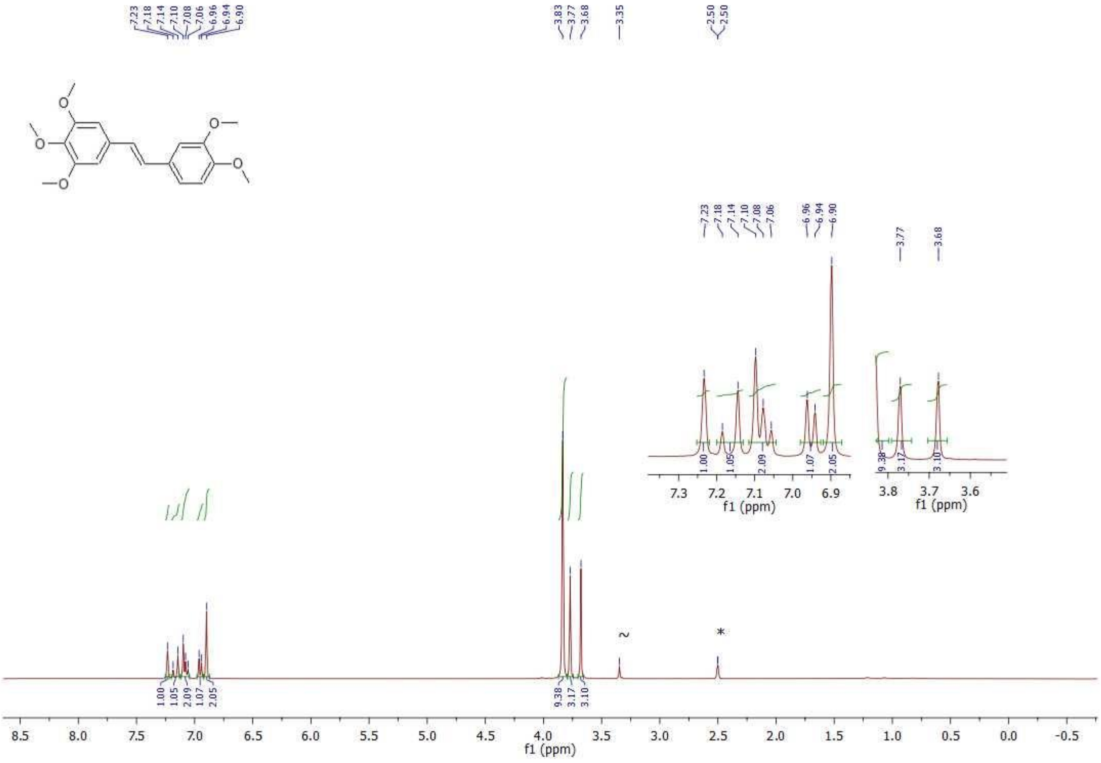

^{13}C NMR

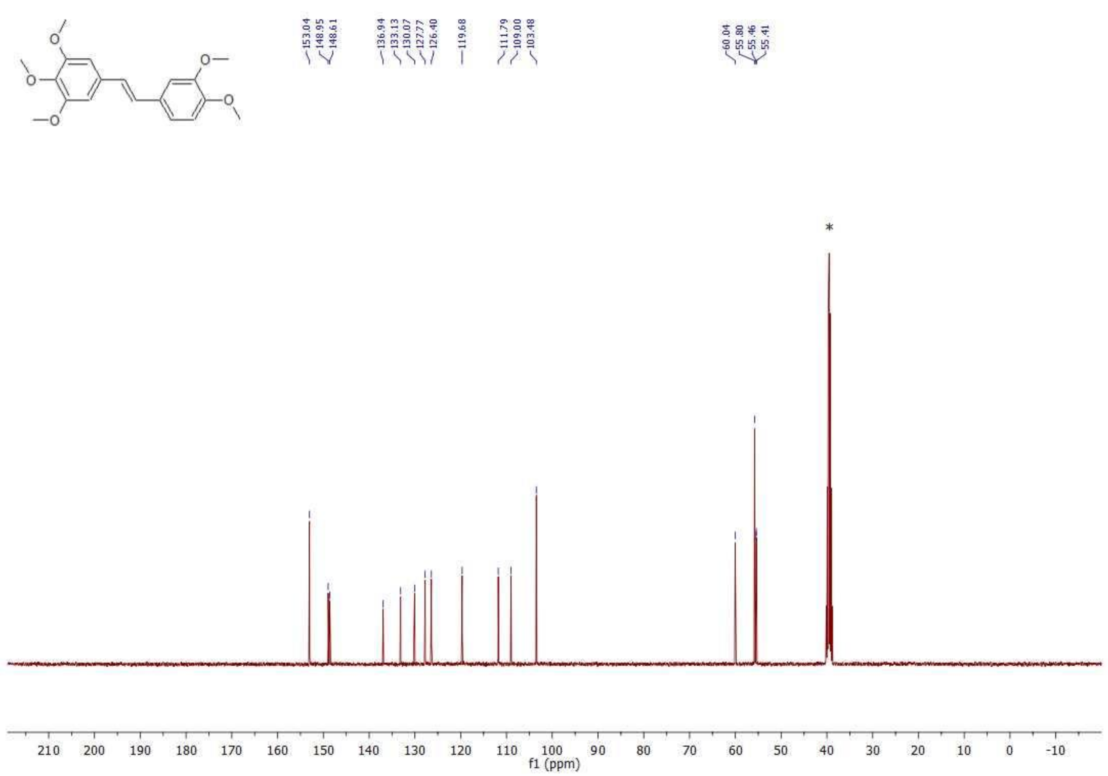

^1H-^1H COSY

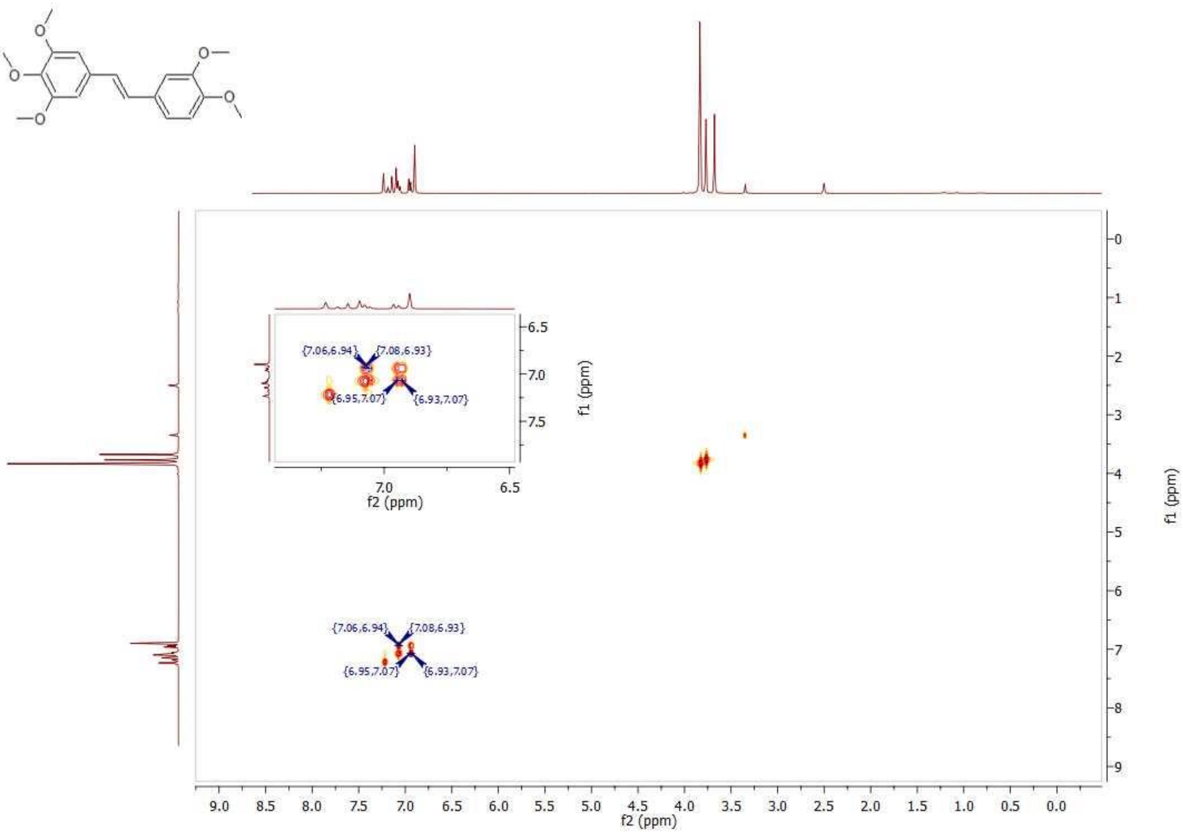

^{1}H-^{13}C HSQC

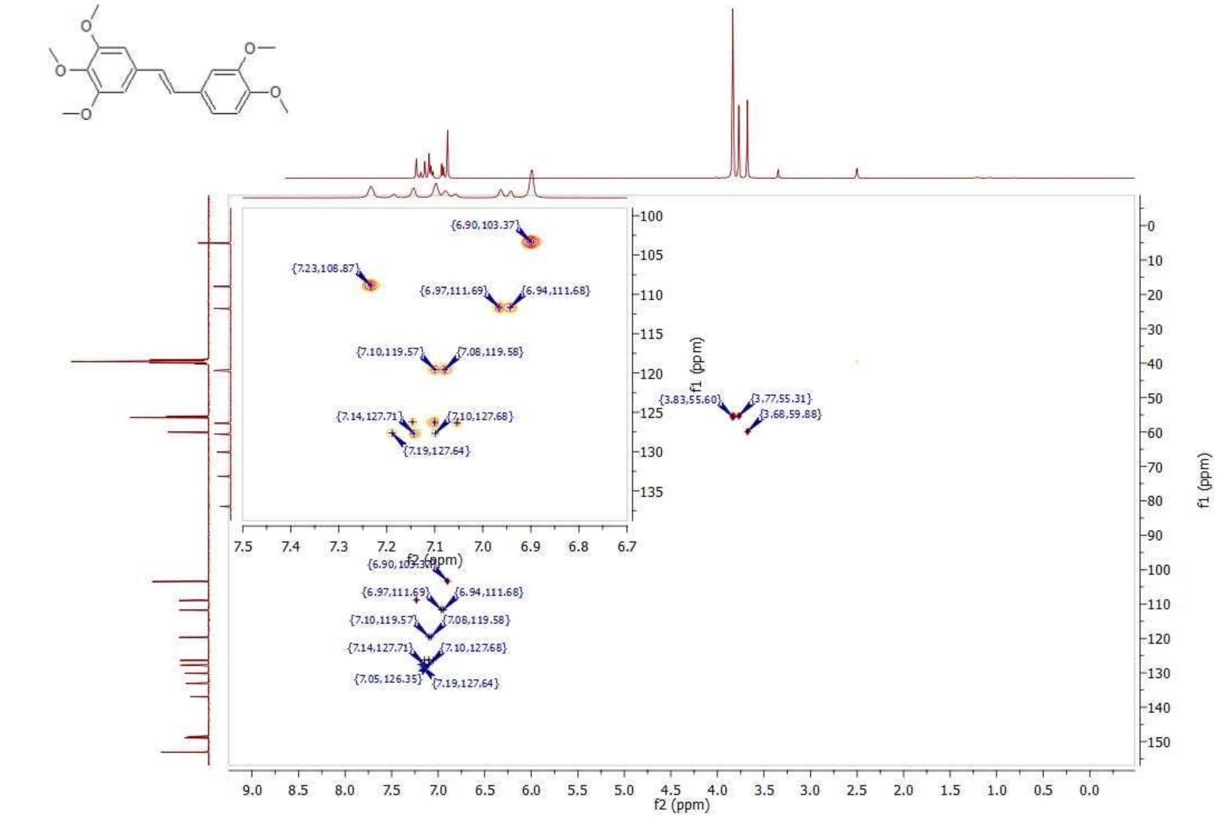

^{1}H-^{13}C HMBC

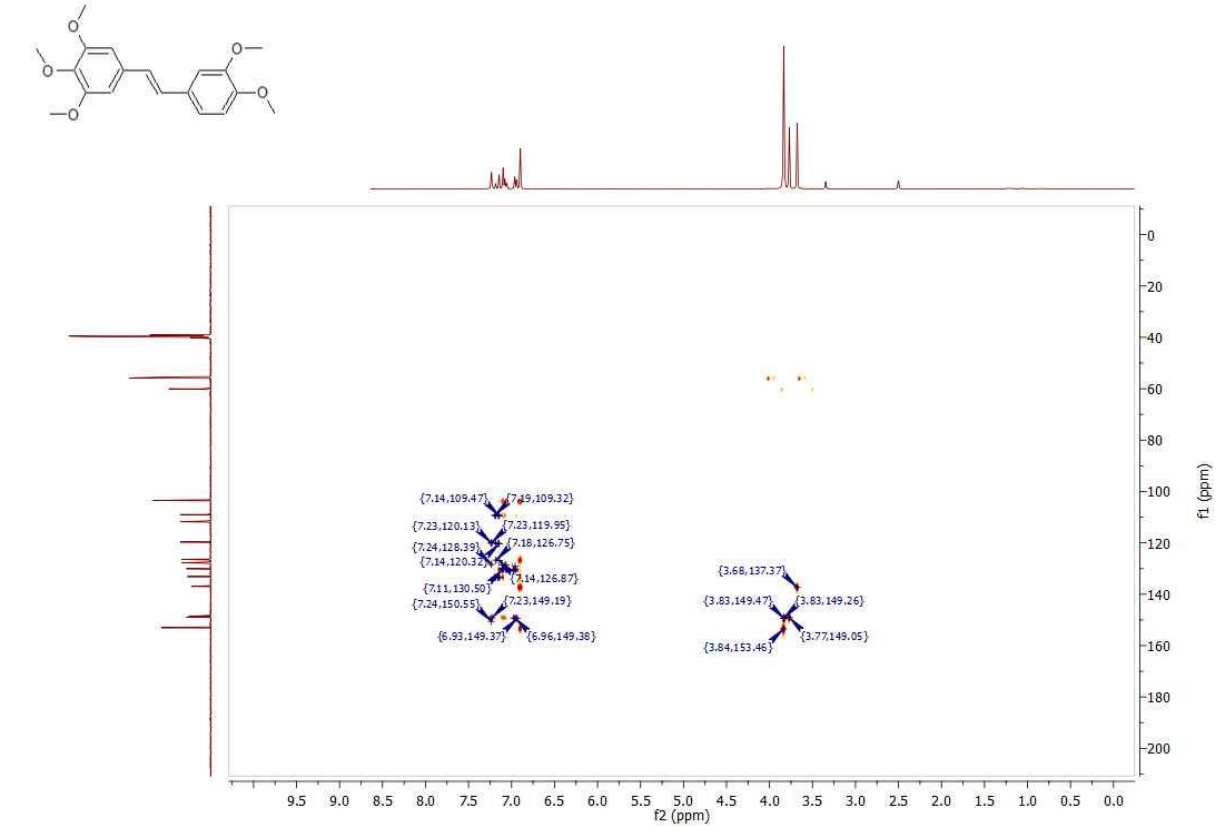

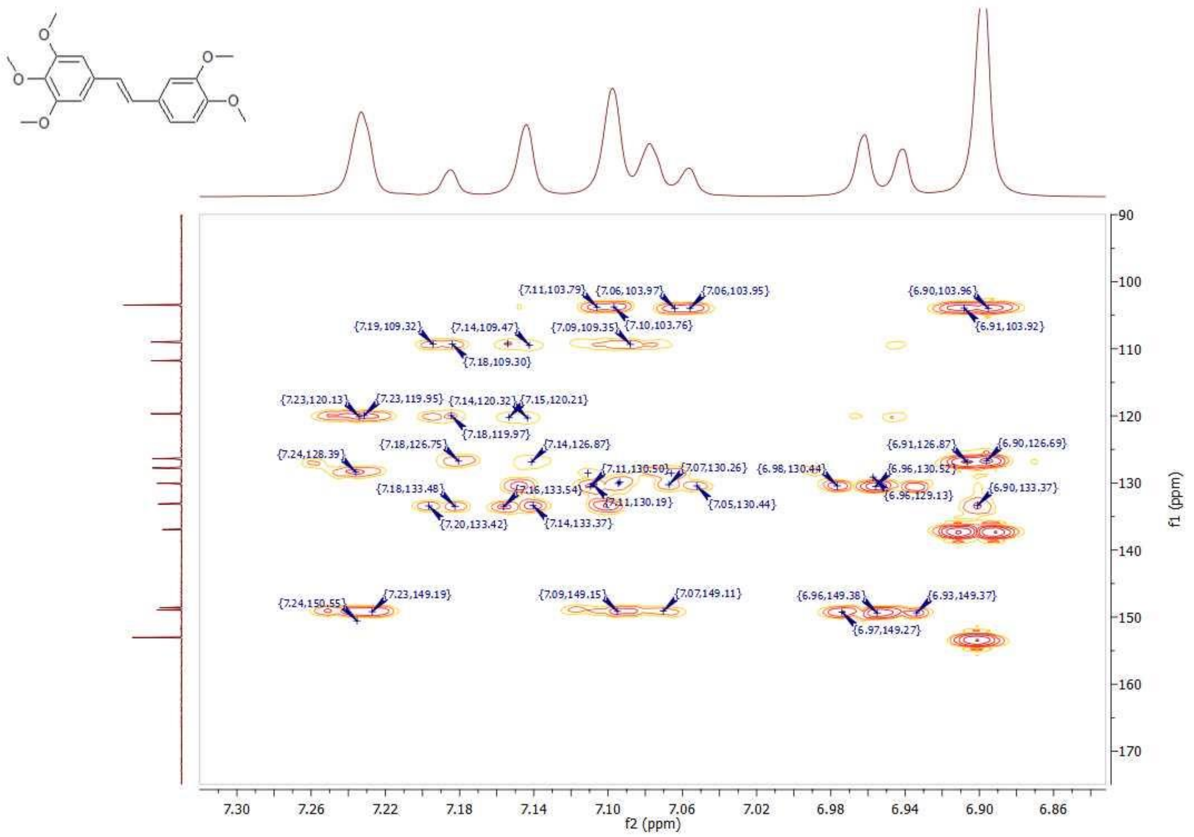
{7.11,103.79}
{7.06,103.97}
{7.06,103.95}
{7.10,103.76}
{6.90,103.96}
{6.91,103.92}
{7.19,109.32}
{7.14,109.47}
{7.09,109.35}
{7.18,109.30}
{7.23,120.13}
{7.23,119.95}
{7.14,120.32}
{7.15,120.21}
{7.18,119.97}
{7.18,126.75}
{7.14,126.87}
{7.24,128.39}
{7.11,130.50}
{7.07,130.26}
{6.98,130.44}
{6.96,130.52}
{6.91,126.87}
{6.90,126.69}
{7.18,133.48}
{7.16,133.54}
{7.11,130.19}
{7.05,130.44}
{6.96,129.13}
{6.90,133.37}
{7.20,133.42}
{7.14,133.37}
{7.24,150.55}
{7.23,149.19}
{7.09,149.15}
{7.07,149.11}
{6.96,149.38}
{6.93,149.37}
{6.97,149.27}
f2 (ppm)
f1 (ppm)

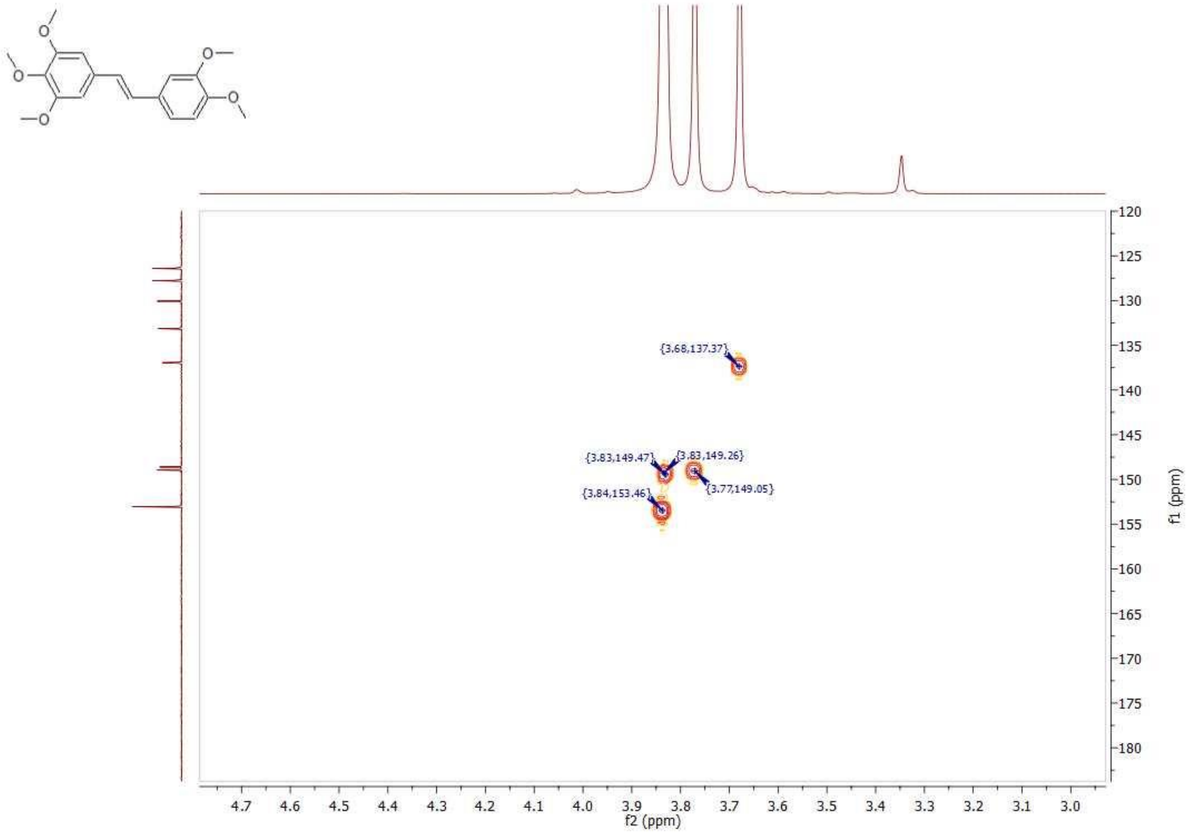
{3.68,137.37}
{3.83,149.47}
{3.83,149.26}
{3.77,149.05}
{3.84,153.46}
f2 (ppm)
f1 (ppm)

5. References

[1] Wierzchowski M, Dutkiewicz Z, Gielara-Korzańska A, Korzański A, Teubert A, Teżyk A, et al. Synthesis, biological evaluation and docking studies of trans-stilbene methylthio derivatives as cytochromes P450 family 1 inhibitors. Chem Biol Drug Des 2017;90:1226–36. https://doi.org/https://doi.org/10.1111/cbdd.13042.

[2] Mikstacka R, Wierzchowski M, Dutkiewicz Z, Gielara-Korzańska A, Korzański A, Teubert A, et al. 3,4,2′-Trimethoxy-trans-stilbene – a potent CYP1B1 inhibitor. Med Chem Commun 2014;5:496–501. https://doi.org/10.1039/C3MD00317E.

[3] Yan RA, Li XX, Li GQ. Trans-3,3′,4,5′-Tetramethoxystilbene. Acta Crystallogr Sect E Struct Rep Online 2011;67:o1960–o1960. https://doi.org/10.1107/S160053681102575X/FF2018ISUP3.CML.

[4] Cross GG, Eisnor CR, Gossage RA, Jenkins HA. Oxazoline chemistry. Part 12: A metal-mediated synthesis of DMU-212; X-ray diffraction studies of an important anti-cancer agent. Tetrahedron Lett 2006;47:2245–7. https://doi.org/10.1016/J.TETLET.2006.01.089.

[5] Vedernikov AI, Basok SS, Gromov SP, Kuz'mina LG, Avakyan VG, Lobova NA, et al. Synthesis and Structure of Bis-crown-Containing Stilbenes. vol. 41. 2005.
